# Inactivation of Competitive Decay Channels Leads to Enhanced Coumarin Photochemistry

**DOI:** 10.1002/chem.202200647

**Published:** 2022-05-12

**Authors:** Robin Klimek, Marvin Asido, Volker Hermanns, Stephan Junek, Josef Wachtveitl, Alexander Heckel

**Affiliations:** ^1^ Institute for Organic Chemistry and Chemical Biology Goethe University Frankfurt Max-von-Laue Str. 9 60438 Frankfurt Germany; ^2^ Institute of Physical and Theoretical Chemistry Goethe University Frankfurt Max-von-Laue Str. 9 60438 Frankfurt Germany; ^3^ Max Planck Institute for Brain Research Max-von-Laue Str. 4 60438 Frankfurt Germany

**Keywords:** ATTO 390, coumarin, fluorophore, photocage, TICT

## Abstract

In the development of photolabile protecting groups, it is of high interest to selectively modify photochemical properties with structural changes as simple as possible. In this work, knowledge of fluorophore optimization was adopted and used to design new coumarin‐ based photocages. Photolysis efficiency was selectively modulated by inactivating competitive decay channels, such as twisted intramolecular charge transfer (TICT) or hydrogen‐bonding, and the photolytic release of the neurotransmitter serotonin was demonstrated. Structural modifications inspired by the fluorophore ATTO 390 led to a significant increase in the uncaging cross section that can be further improved by the simple addition of a double bond. Ultrafast transient absorption spectroscopy gave insights into the underlying solvent‐dependent photophysical dynamics. The chromophores presented here are excellently suited as new photocages in the visible wavelength range due to their simple synthesis and their superior photochemical properties.

## Graphical abstract

**To date**, there is only little information on how to rationally optimize the uncaging efficiency of photocages. However, there are many studies that successfully optimized the performance of fluorophores. This study demonstrates that structural modifications inspired by the fluorophore ATTO 390 can lead to a one order of magnitude increase in the uncaging cross section of new coumarin photocages.

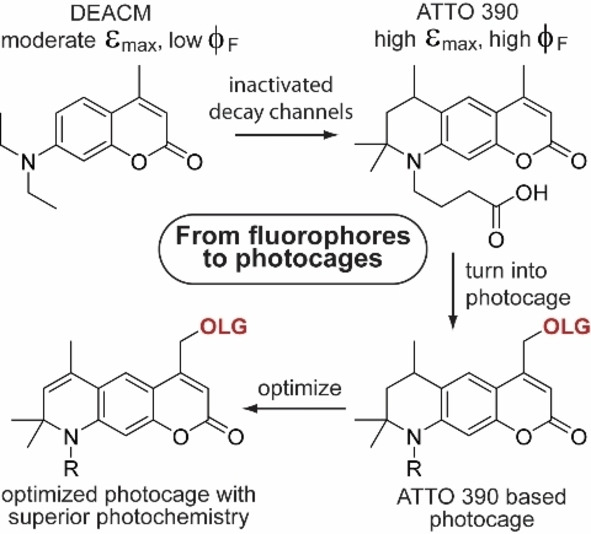

## Introduction

In recent years, the use of light has gained enormous interest in the life sciences. An increasing number of research groups focusses on the use of photolabile protective groups (so‐called “photocages”) to regulate biological processes.[^1^, ^2^, ^3^, ^4^, ^5^] Aside from the application, however, also the development of new photocages is of high interest.[^6^, ^7^] In order to be able to use light for targeted regulation, the photo‐physical and ‐chemical properties of protecting groups have to match the requirements of the biological context. Here, especially two parameters are of crucial importance. The first is the excitation wavelength, preferably in the red range of the visible light spectrum and the second is the uncaging efficiency of the respective photocage. Regarding the first parameter, many studies have been published that focused on shifting the extinction maximum to longer wavelengths.[^8^, ^9^]

**Figure 1:**
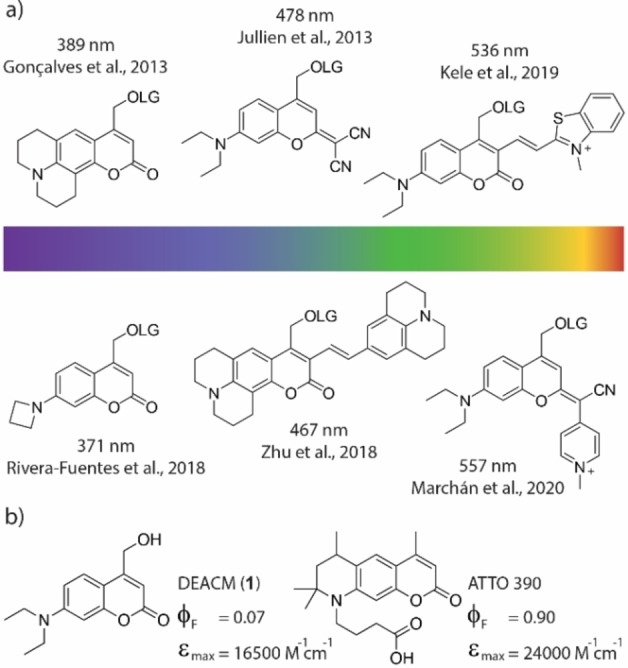
a) Selection of prominent coumarin based photocages in recent years and their reported absorption maxima. LG=leaving group. b) Structures and photophysical properties of DEACM **1** and ATTO 390.^[29]^

Taking the coumarin photocage as an example, popular methods were the extension of the π‐system, or the addition of donor and acceptor substituents (see Figure 1a).[^10^, ^11^, ^12^, ^13^] Also with other photolabile protecting groups, for example BODIPY,[^14^, ^15^] fluorenol,[^16^, ^17^] or nitrobenzyl[^18^, ^19^] a considerable red‐shift was achieved with similar approaches. Nevertheless, the improvement of the second parameter, the uncaging efficiency, has not been studied as systematically in the recent literature. Still, this is highly important, because if the applied light can be used more efficiently, side effects such as cell damage can be reduced.

**Figure 2:**
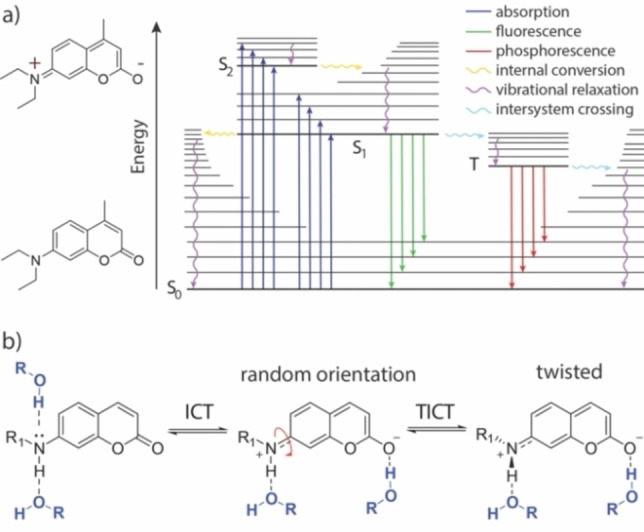
a) Jablonski diagram showing relevant transitions of coumarin chromophores when excited with light. S_0_: ground state, S_1_: first electronically excited singlet state, S_2_: second electronically excited singlet state, T: triplet state. b) General model for twisted intramolecular charge transfer (TICT) in coumarin dyes. In addition, possible hydrogen‐bonding sites are shown, that can affect non‐radiative decay.

Efficient photolysis means that the chromophore must have both a high extinction coefficient (ϵ) and a good uncaging quantum yield (ϕ_u_). To increase the uncaging efficiency of a photocage, it is important to understand what other processes compete with the photolytic cleavage of a leaving group. A molecule in the excited state can release its energy through a variety of pathways (see Jablonski diagram, Figure 2a). In the fast range, there are rotations, vibrations, and isomerization around single atoms or bonds. Also, internal conversion (heat loss to the solvent)^[20]^ or hydrogen‐bonding^[21]^ can lead to relaxation to the ground state.^[22]^ If the excited state lives long enough (e. g. nanoseconds) and does not decay in any of the faster channels, the molecule can also release the energy via luminescence,^[23]^ or a directed bond cleavage.

To date, there is only little information on how to rationally optimize the uncaging quantum yield of photocages. However, there are many advanced studies that have successfully optimized the performance of fluorophores.[^24^, ^25^, ^26^] Extinction coefficients and absorbance maxima have been modified. Unwanted photoreactions (e. g. photobleaching) and the amount of deactivation processes that happen earlier than fluorescence have been reduced. In many cases also the water solubility has been improved. Therefore, a possible strategy to develop new photocages with improved photolysis can be to use an optimized fluorophore with one major decay channel as a guide – an analogy that has recently also been followed by *Rivera‐Fuentes* et al.^[27]^ – and divert the excited state population into an uncaging reaction pathway.

In this study, we explored this idea using ATTO 390[^28^, ^29^] as an example, which has a fluorescence quantum yield of more than 90 % in aqueous solution. Here, we show how to improve the uncaging cross section (ϵϕ_u_) of new ATTO 390‐based photocages by up to one order of magnitude compared to literature known DEACM (**1**) (Figure 1b), applying principles from fluorophore optimization. Using ultrafast transient absorption spectroscopy, we gain insight into the fundamental photophysical processes that are critical both for fluorescence as well as for photolytic cleavage of a leaving group. Overall, we demonstrate that understanding excited state decay channels in fluorophores can be helpful for developing new photocages.

## Results and Discussion

In coumarin fluorophores substituted like the one shown in Figure 2b, it is known that the molecule can adopt an intramolecular charge transfer (ICT) state.[^30^, ^31^, ^32^] After excitation, a rotation around the C−N bond at the donor moiety can occur. Thereby, repulsive interactions are minimized and charge separation is stabilized. The resulting twisted intramolecular charge transfer (TICT) state can release its energy to the medium non‐radiatively.[^33^, ^34^] In addition, as indicated in Figure 2b, it is possible that coumarins release their energy to the environment via hydrogen bonding.[^21^, ^35^, ^36^, ^37^] However, a restricted rotation around the C−N bond is known to supress the TICT and therefore increase fluorescence.^[38]^ This can be observed, for example, with ATTO 390, in which the exocyclic nitrogen atom is fixed by a six‐membered ring. Therefore, ATTO 390 shows a high fluorescence quantum yield in comparison to freely rotating coumarins like DEACM (**1**).

**Figure 3:**
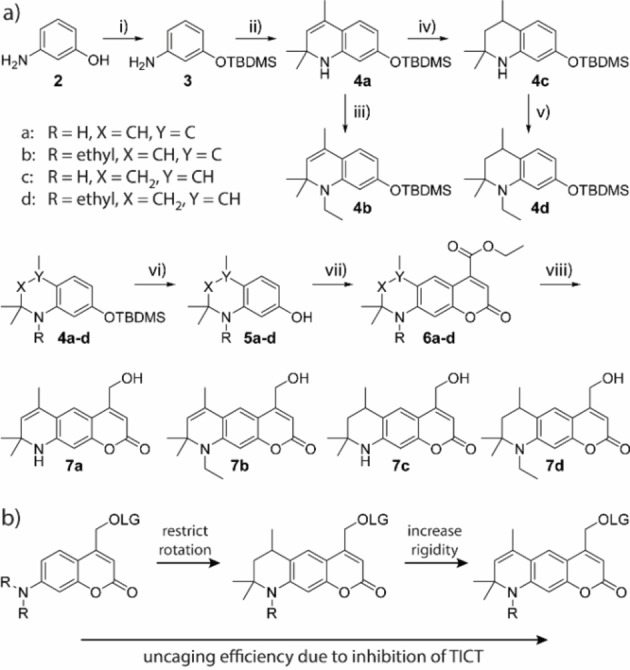
a) Synthesis of compounds **7 a**–**d**. i) TBDMS−Cl, imidazole, DCM, quant. ii) Yb(OTf)_3_, acetone, 77 %. iii) iodoethane, Cs_2_CO_3_, MeCN, 78 %. iv) Pd/C, H_2_, MeOH, 98 %. v) iodoethane, Cs_2_CO_3_, MeCN, 83 %. vi) TBAF, AcOH, THF, 89–94 %. vii) sodium diethyl oxalacetate, EtOH, 31–47 %,. viii) NaBH_4_, MeOH, 37–62 %. TBDMS=tert‐butyldimethylsilyl. b) Evolution of new coumarin‐photocages with improved uncaging efficiency due to inhibition if intramolecular twisting. LG: leaving group, R: H/ethyl, TICT: twisted intramolecular charge transfer.

Guided by this principle, we synthesized four new coumarin photocages **7 a**–**d** (see Figure 3) which have a structure similar to ATTO 390. In all four compounds, the rotation around the C−N bond is restricted by a six‐membered ring. Compounds **7 a** and **7 b** were additionally equipped with a double bond at the 6‐position, which should make the framework even more rigid and the rotation more confined (see Figure 3b). By using this double bond, we hoped to limit non‐radiative energy release on short time scales. The coumarin photocage should be even less capable of forming a TICT. However, derivatives **7 b** and **7 d** also featured an additional ethyl group on the nitrogen atom at the 7‐position. This exhibits an electron donating character compared to the proton in **7 a** and **7 c** and should thus enhance the charge transfer character formation originating from the nitrogen that is necessary for uncaging. The absence of a hydrogen‐atom can also prevent possible non‐radiative decay upon hydrogen‐bonding with protic solvents[^34^, ^35^, ^36^, ^37^, ^39^] and eliminates a potentially photoacidic site.[^40^, ^41^]

The synthesis of compounds **7 a**–**d** started from commercially available *m*‐aminophenol **2** (see Figure 3a). In the first step, the hydroxy group of **2** was protected with *tert*‐butyldimethylsilyl (TBDMS) in a quantitative yield. The resulting compound **3** was reacted with acetone under Yb^3+^ catalysis to quinoline **4 a** in a Doebner‐Miller type reaction.^[42]^ Starting from **4 a**, compound **4 b** was obtained via alkylation with iodoethane and Cs_2_CO_3_ under microwave irradiation and **4 c** was isolated after selective hydration with H_2_, Pd/C under atmospheric pressure in excellent yields. Hydration needed to be performed before the silyl protecting group was removed. Otherwise, we observed cleavage of the C−N bond at 7‐position with H_2_, Pd even at very short reaction times. The reduced compound **4 c** was also alkylated with iodoeth ane to get **4 d**. The TBDMS‐groups of **4 a**–**d** were removed with tetrabutylammonium fluoride (TBAF) and glacial acetic acid in 89–94 % yields to give alcohols **5 a**–**d**. Pechmann‐condensation based on a procedure of Begoyan et al.^[43]^ resulted in coumarin esters **6 a**–**d**. During the reaction, several side products occurred that were hard to separate from the main product by column chromatography. Recrystallization further removed impurities but also resulted in poorer yields. Therefore, we decided to use compounds **6 a**–**d** for further reactions after single column chromatography with minor impurities. After the reduction of the respective esters with NaBH_4,_ the alcohols **7 a**–**d** were isolated in good yields and purities.

**Figure 4:**
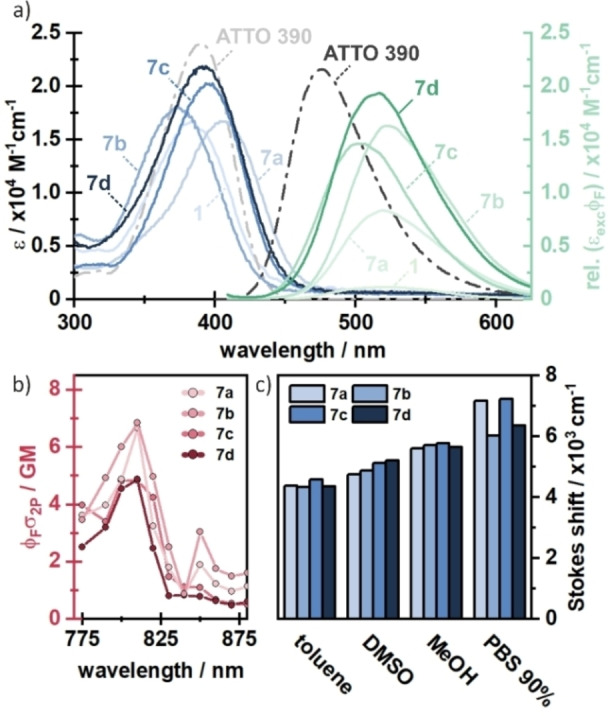
a) Steady‐state absorption and fluorescence spectra of compounds **7 a**–**d** in PBS. Spectra of ATTO 390 (taken from ATTO‐tec)^[29]^ and compound **1** are added for comparison. b) Two‐photon absorption spectra of compounds **7 a**–**d**. c) Stokes shifts in different solvents with different polarity.

To evaluate the photophysical properties of compounds **7 a**–**d**, one‐photon (1P) absorption and fluorescence spectra were recorded in different solvents (see Figure 4 and Table 1). We compared the data to the most commonly used coumarin photocage DEACM (**1**) and ATTO 390 as reference compounds. In PBS (phosphate‐buffered saline), compounds **7 a**–**d** showed higher extinction coefficients in their absorption maxima (ϵ_max_) than DEACM (**1**). Compared to ATTO 390 the ϵ_max_ of **7 a**–**d** were slightly smaller. Compound **7 d**, which has the most similar structure to ATTO 390 has also the lowest difference in the extinction coefficient. In general, an increased extinction coefficient compared to DEACM (**1**) is already a promising start towards the desired increase of the uncaging cross section ϵϕ_u_. The absorption maxima themselves (λ_max_) were located between 373 nm and 405 nm. Thus, they were in the same range as the absorption maxima of DEACM and ATTO 390. The strongest shift was observed for compound **7 b**, which had a 20 nm bathochromic wavelength shift relative to DEACM. Nevertheless, a minimal blue shift of 13 nm could also be observed for compound **7 c**. In comparison, the data indicated that the compounds with the additional ethyl group showed a more red‐shifted absorption spectrum. Compound **7 d** (λ_max_=394 nm) absorbs most similarly to ATTO 390 (λ_max_=390 nm), what again can be rationalized with their very similar structure. The additional double bond in compound **7 b** (λ_max_=405 nm) further increases the absorption wavelength. This red‐shifting effect of the double bond can also be observed when comparing compounds **7 c** (λ_max_=373 nm) and **7 a** (λ_max_=383 nm).

**Table 1:** Photophysical properties of compounds **1**, ATTO 390^[29]^ and **7 a**–**d**.

	ϵ_max_ (PBS) [M^− 1^ cm^−1^]	λ_abs_ (PBS) [nm]	λ_em_ (PBS) [nm]	ϕ_F_ (PBS)	ϕ_F_ (DMSO)
**1**	16500	385	520	0.07	0.74
**ATTO 390**	24000	390	476	0.90	–
**7 a**	16700	383	528	0.50	0.51
**7 b**	18100	405	536	0.90	0.51
**7 c**	20300	373	511	0.72	0.89
**7 d**	21500	394	526	0.95	0.92

To evaluate whether our new coumarin compounds can also be excited with NIR light, we measured two photon absorption spectra of compounds **7 a**–**d**. Here, we observe a two‐photon absorption of 5–7 GM at 800–810 nm for all measured compounds (Figure 4b), which is in the same range as ATTO 390 (13 GM).^[44]^ This 2P‐transition is energetically equivalent to the main absorption maxima found at around 380–410 nm (Figure 4a), indicating that the proposed compounds can be one‐ or two‐photon activated. This measurement shows that excitation is also possible in the so‐called phototherapeutic window (650–950 nm), in which light has a particularly high penetration depth into biological tissue.^[45]^

To get a first impression of how the newly synthesized chromophores release their excitation energy, we measured fluorescence quantum yields (see Table 1). Since fluorescence and uncaging occur on approximately the same time scale, this parameter may provide a first indication of successful inactivation of faster decay channels. Additionally, comparing polar solvents (such as PBS) to less polar ones (such as DMSO) may indicate whether TICT is involved. For medium polar solvents like DMSO, it is known that coumarin charge separation is only stabilized to a limited extent, resulting in a high ϕ_F_, independent of their flexibility.^[46]^ It is also possible that rotation around the C−N bond is hindered in solvents with relatively high viscosity, such as DMSO.^[47]^

In aqueous solution, however, the amine substituent can rotate freely if it is not fixed by a six‐membered ring, since water is less viscous. This is in agreement with the measured high ϕ_F_ for compounds **7 a**–**d** and DEACM (**1**) in DMSO, ranging between 51 % and 95 %. When DMSO was replaced by PBS, the ϕ_F_ of DEACM (**1**) was reduced from 74 % to 7 % whereas fluorescence of **7 a**–**d** remained on a high level. This reduction in fluorescence suggests that rotation around the C−N bond in DEACM is possible in less viscous PBS, but it is inhibited in all newly synthesized derivatives **7 a**–**d**. In turn, this points towards a TICT state in DEACM, which is hindered in compounds **7 a**–**d**. The fluorescence quantum yields further show that the two compounds with the ethyl group **7 b** and **7 d** emit best in PBS. This can be explained by the fact that the ethyl group can stabilize the charge separation more effectively over a longer period of time. A similar quantum yield in PBS (90 %) is shown by ATTO 390 itself, which also carries an alkyl group at the nitrogen atom.

To investigate the effect of stabilization of charge separation in more detail, we also studied the Stokes shifts in different polar solvents (see Figure 4c). Again, the polarity of the solvent is expected to have a strong influence on the emission wavelength. We observed that all compounds **7 a**–**d** showed already large Stokes shifts in unpolar solvents (4334–4583 cm^−1^ in toluene). When the polarity of the solvent was increased, an overall rise in the Stokes shifts was observed. The largest Stokes shifts were measured in PBS buffer (6035–7240 cm^−1^), supporting that there is charge transfer in all molecules which is stabilized by the solvent to different degrees.

**Figure 5:**
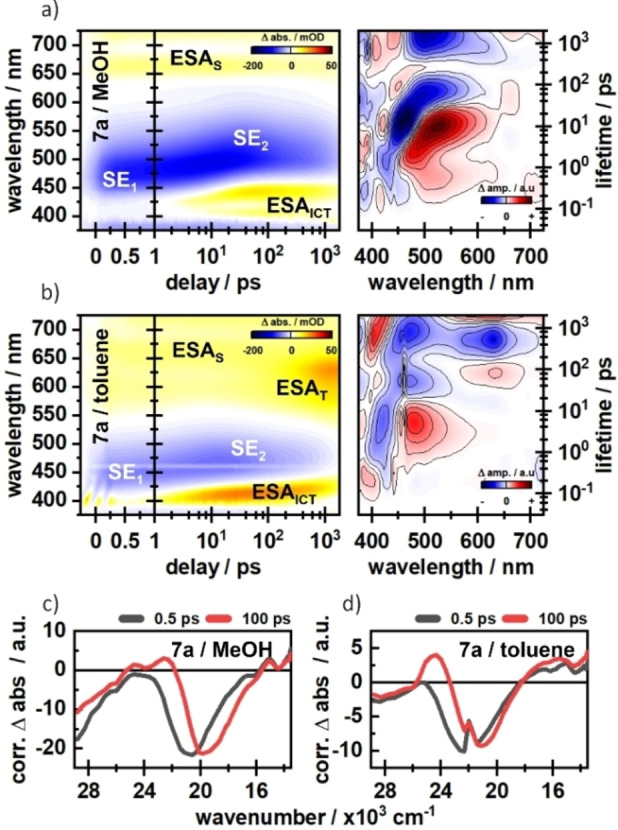
Transient absorption spectra of compound **7 a** in MeOH (a) and toluene (b). Time slices at 0.5 ps and 100 ps underline the dynamic stokes shifts in c) MeOH and d) toluene.

To gain an even deeper insight into the photophysical dynamics of the newly synthesized compounds, we employed ultrafast UV/vis transient absorption spectroscopy in different solvents. Here, the general spectral features of these compounds shall be explained exemplarily in the case of compound **7 a** in MeOH (see Figure 5a). The photoexcitation from the ground state leads to a broad excited state absorption (ESA_S_) signature, which spans from 620 nm up to more than 730 nm. Simultaneously, the stimulated emission (SE_1_) from S_1_ to S_0_ can be found in the range of 430–550 nm, which is consistent with the steady state fluorescence spectra. After about 300–500 fs, an additional absorption signal (ESA_ICT_) appears at approximately 400 nm and 430 nm. The amplitude of the 430 nm component rises further on the 1–50 ps timescale, which is accompanied by a decrease of the ESA_S_ band and a strong red‐shift of the stimulated emission by 40 nm forming SE_2_ (Figure 5a and 5c). The latter effect is related to the excited state intramolecular charge transfer (ICT) from the nitrogen of the amine group to the oxygen of the carbonyl group, which is further stabilised by the solvent response on the ps timescale.

In contrast to compound **7 a**, it can be observed that in compound **7 b** (Figure S1b, Supporting Information) the blue component of the ESA_ICT_ feature at around 400 nm is mainly populated, which means that the excited state is more stabilized in **7 b** relative to **7 a**. The less pronounced stabilization of the excited state in **7 a** most likely results from a hydrogen bond at the nitrogen, which is formed in addition to the stabilizing hydrogen bond at the carbonyl group.^[27]^ In toluene, compound **7 a** shows a miniscule dynamic Stokes‐shift of the SE band (Figure 5b and 5d). However, the ESA_ICT_ is still formed, indicating a population of an ICT state, which is less stabilised by the surrounding solvent. Instead, an additional absorption (ESA_T_) around 600 nm is formed on the 100 ps timescale, which we tentatively assign to triplet formation. A similar ESA_T_ signature is found for **7 a** in DMSO (Figure S1c, Supporting Information), which is also aprotic but significantly more polar. Therefore, the excited state dynamics are not just strongly influenced by the solvent polarity, but also by the solvent proticity and the general accessibility of hydrogen bonding interactions. Besides the obvious stabilization of the excited state in polar solvents, the effect of solvent proticity is more complicated, since there are different sites for hydrogen bonding. Whereas hydrogen bonds with the carbonyl oxygen or the nitrogen‐bound proton are reported to have a stabilising effect on the excited state, a hydrogen bond with the lone‐pair of nitrogen has the opposite effect.[^35^, ^36^, ^37^] The reduction of energy in the ICT state due to these effects leads to a favourable ICT‐population. In contrast, the population of the triplet state is seemingly facilitated in the case of missing hydrogen bonding.[^48^, ^49^, ^50^] Further details on the solvent effects as well as cross‐comparisons between the compounds are given in the Supporting Information of this article. The transient absorption measurements demonstrated that all four new compounds form an ICT state. The ethyl group in compound **7 b** seems to further stabilize the charge separation in comparison to compound **7 a** as can be seen in the significantly increased ESA_ICT_ amplitudes (Figure S1, Supporting Information). However, this effect is not seen in the direct comparison of compounds **7 d** and **7 c** (see Figure S2, Supporting Information), which lack the additional double bond.

**Figure 6:**
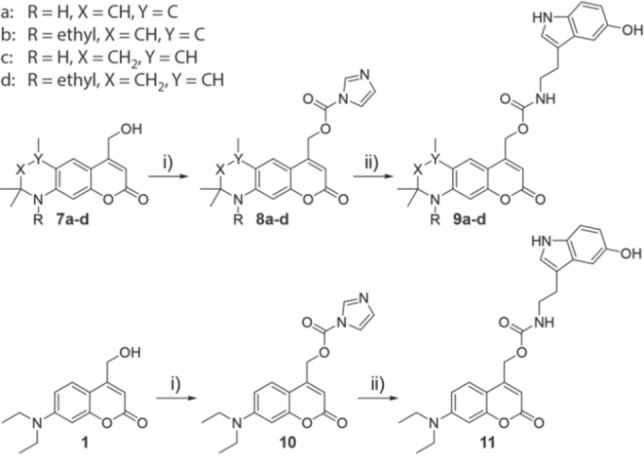
Synthesis of compounds **9 a**–**d** and **11**. i) CDI, DCM, 79–89 %. ii) 5‐Hydroxytryptamine hydrochloride, Et_3_N, DMF, 56–77 %.

But even more interesting to us was, whether the added rigidity of the new compounds would also lead to improved photolysis. Therefore, we attached serotonin as a biologically active leaving group (see Figure 6). Serotonin is an important neurotransmitter that plays a crucial role in signal transduction in the central nervous system.^[51]^ The synthesis started by reacting compounds **7 a**–**d** with carbonyldiimidazole (CDI) under microwave irradiation resulting in **8 a**–**d**. To generate the final photolabile protected serotonin‐derivatives **9 a**–**d**, compounds **8 a**–**d** were reacted with 5‐hydroxytryptamine hydrochloride. The same procedure was used to synthesize DEACM‐protected serotonin **11**, starting from DEACM (**1**) as a reference.

After preparation we performed first photolysis tests in PBS/DMSO (9 : 1). We added this small amount of DMSO because the photocages were only moderately soluble in pure PBS. To make the photolysis results comparable, the quantum yields for compounds **9 a**–**d** and **11** were determined at 405 nm using a fulgide derivative as actinometer.^[52]^ The decrease of starting materials was monitored by HPLC with the addition of an internal standard. After the experiments, we were surprised that the uncaging quantum yields for compounds **9 a**–**d** and **11** differed only minimally from each other. This contradicted all assumptions from previous literature and the earlier determined fluorescence behaviour of the compounds. The fluorescence experiments indicated that competitive relaxation pathways in compounds **7 a**–**d** were blocked which in turn leads to an increased fluorescence quantum yield compared to DEACM. Since fluorescence and uncaging occur on the same time scale, the uncaging quantum yield of compounds **9 a**–**d** should also be enhanced in direct comparison to reference compound **11**.

To investigate this unexpected outcome in more detail, we performed ultrafast transient absorption spectroscopy in PBS/DMSO (9 : 1). Interestingly, in this solvent, the amplitudes of the difference signals are significantly reduced (Figure S1b and f, S2b and f). This becomes most obvious in the ESA_ICT_ band, which is the dominant feature in all measurements. In contrast to the other tested solvents, the ESA_ICT_ does not show a major rise in amplitude on the 10–100 ps timescale in PBS/DMSO (Figure S3), implying a less pronounced population of the CT state. However, it was shown in recent work^[53]^ that the charge separation is crucial in order to facilitate elongation of the C−C bond, which is cleaved in the subsequent uncaging process. Additionally, the solvent response due to the ultrafast change in dipole moment is somewhat more complicated in PBS/DMSO mixtures. It is reported that DMSO and H_2_O form strong hydrogen bond clusters even at small amounts of DMSO.[^54^, ^55^] For simple coumarin‐based compounds this means that the solvent reorganization in the excited state is mostly driven by DMSO, which “drags” H_2_O along through their H‐bond network.^[56]^ It is therefore possible that DMSO at the photochemically relevant site suppresses photocleavage in a PBS/DMSO mixture due to the less pronounced CT character and a possible shielding from H_2_O. The latter aspect is especially relevant since water molecules (or other nucleophiles) are needed to capture the carbo‐cation which is formed during uncaging.^[57]^

**Figure 7:**
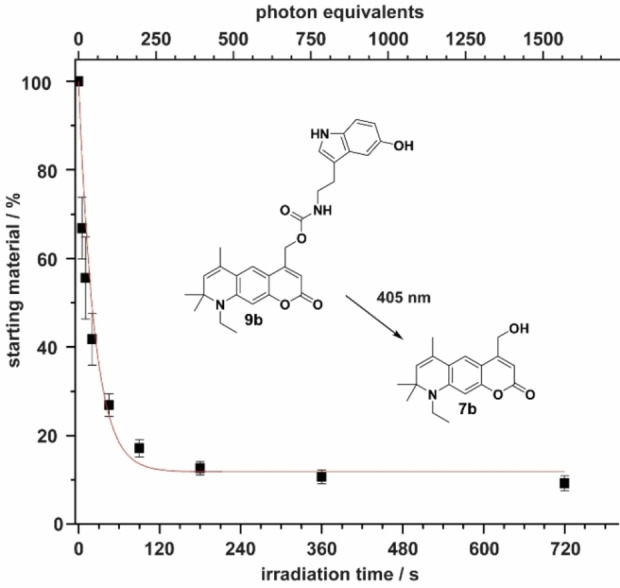
Exemplary photolysis curve of compound **9 b** in PBS/MeOH (1 : 1). Photolysis product **7 b** was identified via mass spectrometry.

Therefore, we substituted PBS/DMSO with PBS/MeOH (1 : 1) in further experiments, because MeOH and H_2_O do not interact as strongly, and MeOH itself can serve as a nucleophile to trap the exocyclic carbocation in the coumarin scaffold formed during uncaging. After changing the solvent, we were now able to see clear differences in uncaging quantum yield between the newly synthesized compounds **9 a**–**d** and the reference DEACM derivative **11**. The photolysis results are shown in Table 2. An exemplary photolysis curve of compound **9 b** is shown in Figure 7, the remaining curves are shown in Figures S6–S10. Our experiments show, that the uncaging quantum yield (ϕ_u_) was already significantly increased by the ring closure in **9 c** and **9 d**. While **11** releases serotonin with a ϕ_u_ of 0.56 %, it is increased by a factor of 2–3 in compounds **9 c** (0.96 %) and **9 d** (1.63 %). This already shows the positive effect of the restricted rotation. If an additional double bond is now introduced, a further increase in the uncaging quantum yield can be observed. Compound **9 a** releases serotonin with a quantum yield of 2.56 % and **9 b** even with 2.97 %. As expected, this increase in quantum yield clearly indicates that the additional restricted rotation suppresses competitive decay channels. Therefore, a more than fivefold increase in uncaging quantum yield was observed for **9 b** compared to **11**. The improvement of the photochemical properties becomes even more obvious if the extinction coefficient ϵ at the excitation wavelength is included in order to study the uncaging cross section (ϕ_u_ϵ). It can be seen in Table 2, that solely adding a six‐membered ring leads to an increase by a factor of 3–4 compared to DEACM. When the double bond is additionally introduced, the uncaging cross section at 405 nm raises by one order of magnitude. While **11** photolyzes with 50 L mol^−1−^ cm^−1^ at 405 nm, the best compound **9 b** shows 617 L mol^−1^ cm^−1^. Compound **9 d**, on the other hand, which has a similar structure as **9 b** only without a double bond, has a cross section of 184 L mol^−1−^ cm^−1^. At this point, it is clear that the simple introduction of a double bond can lead to a more than threefold increase in photolysis efficiency.

**Table 2:** Photophysical and photochemical properties of compounds **9 a**–**d** and reference DEACM **11** at 405 nm used for uncaging experiments in PBS/MeOH (1 : 1).

	ϕ_u_ (405 nm) (PBS/MeOH)	ϵ (405 nm) (PBS/MeOH) [M^−1^ cm^−1^]	ϕ_u_ϵ (405 nm) (PBS/MeOH) [M^−1^ cm^−1^]
**11**	0.0056	9097	50
**9 a**	0.0256	19666	504
**9 b**	0.0297	20788	617
**9 c**	0.0163	10606	173
**9 d**	0.0096	19268	184

**Figure 8:**
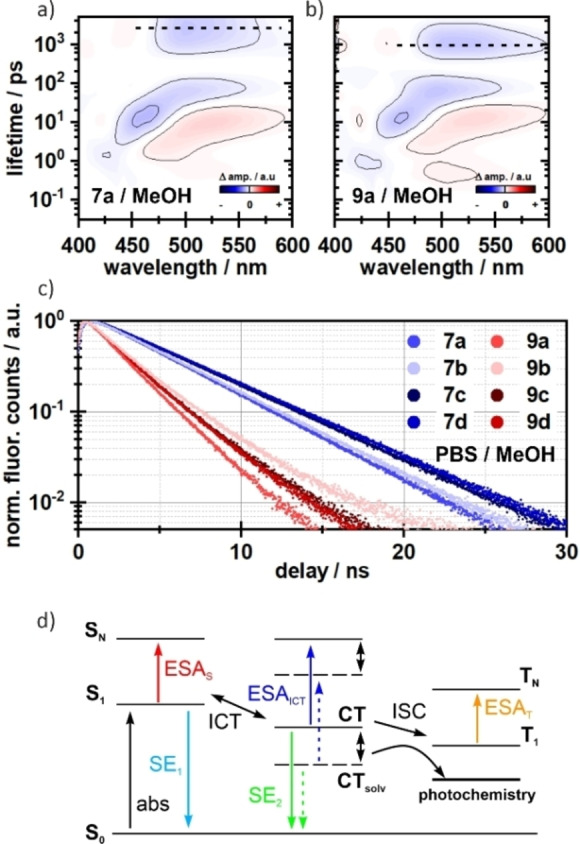
Lifetime density analysis of **7 a** (a) and **9 a** (b) measured in MeOH. The ES decay is faster in compound **9 a** (indicated as dashed lines), whereas the dynamics on the fs‐ to ps‐timescale do not differ. A similar pattern is seen in the TCSPC measurements (c). The fluorescence lifetimes of compounds **9 a**–**d** are roughly 2‐fold faster. The corresponding lifetime components are given in the Supporting Information. d) Schematic representation of possible energy pathways. After excitation, an equilibrium between the locally excited state S_1_ and the CT state is formed. The CT state is further stabilized by the solvent (indicated by double‐sided arrows), which increases the population of the CT state. Without this stabilization an additional channel for triplet formation becomes relevant, which then competes with the actual photochemistry.

In the last step, we wanted to investigate whether uncaging as an additional excitation pathway can also be observed using ultrafast UV/vis pump‐probe transient absorption spectroscopy. For this purpose, we sought to compare the derivatives with leaving group (**9 a**–**d**) with those previously measured without leaving group (**7 a**–**d**, see Figure 5). The measurements of **9 a**–**d** revealed very similar photophysical signatures compared to **7 a**–**d**. All compounds show the characteristic solvent‐ and time‐dependent ESA and Stokes shifts on the picosecond timescale. More interestingly, we observed that the excited state (ES) decay of **9 a**–**d** is shifted towards earlier times compared to **7 a**–**d**, which can be seen in the lifetime density distribution of the respective measurements (Figures 8a and 8b, and Figure S5). To validate this temporal shift we performed time correlated single photon counting (TCSPC) measurements in PBS/MeOH (1 : 1) (Figure 8c). These experiments showed a very clear difference in the fluorescence lifetimes of **7 a**–**d** and **9 a**–**d**. While the fluorescence lifetimes of **7 a**–**d** are found to be between 4.6 and 5.4 ns, the fluorescent states of **9 a**–**d** are depopulated between 2 and 2.6 ns (Table S1), being consistent with the lifetimes found in the TA‐experiments (Figure S5). This more than 2‐fold reduction of radiative decay lifetimes is most likely due to added possibility to undergo photocleavage, which is competing with fluorescence. An overview of the proposed energy pathways is given in Figure 8d.

## Conclusion

In this work, we show a systematic investigation of uncaging efficiency of coumarin photolabile protecting groups. Guided by the principles of fluorophore optimization, we wanted to reduce competitive decay channels on new ATTO 390‐based photocages. To improve the photolysis, in a first step, the rotation around the electron donor on the coumarin scaffold was prevented using a simple six‐membered ring (compounds **7 c** and **7 d**). This already increased the uncaging efficiency by a factor of 3–4 compared to DEACM (**1**). However, the simple introduction of an additional double bond on that six‐membered ring had an even stronger effect. Again, the efficiency of light‐induced release of serotonin was increased by a factor of 2–3 (compounds **6 a** and **6 b**). This is the first time that such a minimal change to the coumarin backbone has resulted in a significant improvement in photolysis efficiency. We used ultrafast transient absorption spectroscopy to investigate the underlying photophysical dynamics. The newly synthesized photocages showed strong solvent dependencies in their fluorescence and uncaging properties. It became clear that the charge‐transfer state in coumarin chromophores is not just well stabilized by polar solvents, but also essential for photolysis. The new photocages described here are easy to synthesize and, due to their photophysical properties, can be used for both one‐photon applications using blue light and in the phototherapeutic window (700–900 nm) using two‐photon excitation. In summary, we think that the development of photolabile protecting groups can indeed very well profit from what has already been learned in the optimization of fluorophores. This can be a good guiding principle for future development.

## Experimental Section

**Chemical synthesis**: All reactions were performed under an argon atmosphere unless otherwise specified. Solvents and reagents were purchased from commercial sources. DEACM **1** was synthesized according to literature.^[58]^ Synthetic procedures of new compounds and their characterization are provided in the Supporting Information. Microwave reactions were performed in a *Biotage Initiator* system. NMR spectra of new compounds were recorded on (250 MHz, 300 MHz, 400 MHz, 500 MHz and 600 MHz) *Bruker* instruments. MALDI HRMS spectra were recorded on a *ThermoScientific MALDI LTQ Orbitrap XL* instrument.

**Ultrafast TA‐spectroscopy**: The time‐resolved transient absorption measurements were performed using a home‐built pump‐probe setup. A Ti : Sa chirped pulse regenerative amplifier (MXR‐CPA‐iSeries, Clark‐MXR Inc., USA) with a central output wavelength of 775 nm, a 1 kHz repetition rate, and a pulse width of 150 fs was used as the fs‐laser source. The fundamental was split for pump and probe pulse generation. Pump pulses at a central wavelength of 388 nm were generated in a second harmonic generation (SHG). The temporal FWHM of the final pump pulses was determined to be ∼120–130 fs. The excitation energy was set to 90 nJ/pulse at the sample position. The supercontinuum for the probe pulses was generated by focusing the fundamental in a constantly moving CaF_2_ window of 5 mm thickness, leading to stable white light in the range of 375–740 nm. The white light was then split and guided through the sample and the reference arm of the detection setup. Each arm makes use of a spectrograph (Multimode, AMKO, Germany), which is equipped with two gratings (300 nm/500 nm blaze, 600/1200 grooves per mm), a photodiode array (S8865‐64, Hamamatsu Photonics, Japan) and a corresponding driver circuit (C9118, Hamamatsu Photonics, Japan). The signals were digitized by a 16 bits data acquisition card (NI‐PCI‐6110, National Instruments, USA). The pump and probe pulses were set to the magic angle configuration at 54.7° to account for anisotropic effects. The sample‐compartment was constantly moved to minimize sample degradation.

**Fluorescence lifetimes**: The fluorescence decays of the compounds **7 a**–**d** and **9 a**–**d** were determined by the time‐correlated single photon counting (TCSPC) technique. Our home‐built TCSPC setup is composed of a single‐photon detection photomultiplier tube (PMA‐C 182 M, PicoQuant, Germany) and a PCIe card (TimeHarp 260 PICO Single, PicoQuant) for sub‐ns data processing. Pulsed orthogonal excitation of the samples was achieved by a pulsed LED (PLS360, PicoQuant) with a peak wavelength of 360 nm and a FWHM <800 ps. Deconvolution with the IRF and multi‐exponential fitting of the temporal traces was performed with FluoFit Pro 4.6 (PicoQuant) based on the following equation^[59]^

$$I\left(t\right)=\int_{-\infty }^{t}IRF\left(t'\right)\sum_{i=1}^{n}A_{i}e^{-\frac{t-t_{i}}{\tau_{i}}}dt'$$

The samples were measured in 4×10 mm quartz glass cuvettes with an OD of ∼0.1 on 10 mm optical pathlength.

**Determination of quantum yields**: Three stock solutions of each compound **9 a**–**d** and **11** were prepared by diluting approx. 1.0 mg in MeOH/PBS (1 : 1). For photolysis 50 μL were taken and irradiated at nine different irradiation times (t=0 s, 5 s, 10 s, 20 s, 45 s, 90 s, 180 s, 360 s, 720 s) with a 405 nm LED (*Thorlabs*), resulting in 27 differently irradiated solutions for each compound. The photon flux for the setup was determined with a fulgide‐derivative – as described in literature.^[52]^ The photolysis was analyzed via RP‐HPLC (*Agilent 1200*) as the ratio of the peak areas of starting material and uridine as internal standard – as described in literature.^[16]^

## Conflict of interest

The authors declare no conflict of interest.

1

## Supporting information

As a service to our authors and readers, this journal provides supporting information supplied by the authors. Such materials are peer reviewed and may be re‐organized for online delivery, but are not copy‐edited or typeset. Technical support issues arising from supporting information (other than missing files) should be addressed to the authors.

Supporting Information

## Data Availability

The data that support the findings of this study are available in the supplementary material of this article.
