# Supplementary material for: Inactivation of Competitive Decay Channels Leads to Enhanced Coumarin Photochemistry
Source: Chemistry. 2022 May 12;28(35):e202200647. doi: 10.1002/chem.202200647 (PMC9320935; doi:10.1002/chem.202200647)
Supplement: Supplementary file 1 — Supporting Information [file CHEM-28-0-s001.pdf]

Chemistry–A European Journal

Supporting Information

Inactivation of Competitive Decay Channels Leads to Enhanced Coumarin Photochemistry

Robin Klimek, Marvin Asido, Volker Hermanns, Stephan Junek, Josef Wachtveitl,* and Alexander Heckel*

Table of content

Chemical Synthesis 2
Two-photon excitation fluorescence experiments 14
Time-correlated single photon counting experiments 14
Femtosecond UV/vis-pump-probe experiments 15
Kinetic analysis of the ultrafast spectroscopic data 15
Supplementary Data 16
Ultrafast Transient Absorption Data 16
Dynamic Stokes’ Shifts – Solvent Dependence 18
Lifetime density analysis of 7a-d and 9a-d 19
Lifetimes obtained by TCSPC 19
Determination of quantum yields 19
NMR spectra 23
Mass spectra of key compounds 47
References 51

Chemical Synthesis

Compound 3:

m-Aminophenol (6.0 g, 55.0 mmol, 1 eq), TBDMS-Cl (41.4 g, 275.0 mmol, 5 eq) and imidazole (26.2 g, 385.0 mmol, 7 eq) were suspended in 200 mL dry THF. The suspension was stirred for 72 h at room temperature. After removal of the solvent under reduced pressure, the residual oil was co-evaporated with EtOH three times. The crude product was purified *via* column chromatography (DCM). The solvent was removed under reduced pressure to give the desired product as light-brown oil.

Yield: 12.2 g (99%)

TLC: DCM, R_f = 0.40

^{1}H-NMR (400 MHz, DMSO-d_6): δ = 6.84 (td, 1H, J = 8.0, 1.9 Hz), 6.15 (d, 1H, J = 8.0 Hz), 6.08 (d, 1H, J = 1.8 Hz), 5.97 (d, 1H, J = 8.0 Hz), 4.99 (s, 2H, H7), 0.95-0.91 (m, 9H), 0.16-0.13 (m, 6H) ppm.

^{13}C-NMR (101 MHz, DMSO-d_6): δ = 155.85, 150.02, 129.45, 107.59, 105.38, 54.88, 25.57, -4.46 ppm.

MALDI HRMS (m/z): calc. for $C_{12}H_{21}NOSi$ $[M+H]^+$ 224.14652, found 224.14642 (Δm = 0.00010, error 0.45 ppm).

Compound 4a:

Compound **3** (10.0 g 44.8 mmol, 1 eq), was dissolved in 300 mL dry acetone. Under continuous stirring $Yb(OTf)_3$ (1.94 g, 3.13 mmol, 0.07 eq) was added and the mixture was reacted for 24 hours at room temperature. The solvent was removed under reduced pressure and the crude product was purified *via* column chromatography (DCM) to give **4a** as a light-brown solid.

Yield: 10.50 g (77%)

TLC: DCM, R_f = 0.79

^{1}H-NMR (400 MHz, DMSO-d_6): δ = 6.78 (d, 1H, J = 8.2 Hz), 5.99 (d, 1H, J = 2.4 Hz), 5.92 (dd, 1H, J = 8.2, 2.4 Hz), 5.77 (s, 1H), 5.14-5.11 (m, 1H), 1.84 (d, 3H, J = 1.3 Hz), 1.16 (s, 6H), 0.93 (s, 9H), 0.16 (s, 6H) ppm.

^{13}C-NMR (101 MHz, DMSO-d_6): δ = 155.46, 145.47, 127.15, 125.98, 124.08, 114.55, 106.77, 103.38, 51.11, 30.95, 25.57, 18.25, 17.90 ppm.

MALDI HRMS (m/z): calc. for $C_{18}H_{29}NOSi$ $[M+H]^+$ 304.20912, found 304.20920 (Δm = 0.00008, error 0.26 ppm).

Compound 4b:

Compound **4a** (2.06 g, 6.8 mmol, 1 eq) and Iodoethane (1.64 mL, 20.4 mmol, 3 eq) were dissolved in 15 mL dry MeCN in a microwave vessel. Cs_2CO_3 (5.31 g, 16.3 mmol, 2.4 eq) was added and the resulting suspension was reacted for 7 h at 100 °C in a microwave. After cooling to room temperature the mixture was flushed with 100 mL DCM und washed with 100 mL dest. water. The aqueous layer was extracted with DCM four times, the organic layers were

combined and the solvent was removed under reduced pressure. The crude product was purified *via* column chromatography (cyclohexane/EtOAc 50:1) to give **4b** as a brown oil.

Yield: 1.76 g (78%)

TLC: cyclohexane/EtOAc 50:1, R_f = 0.27

^{1}H-NMR (500 MHz, DMSO-d_6): δ = 6.82 (d, 1H, J = 8.2 Hz), 5.98 (dd, 1H, J = 8.1, 2.2 Hz), 5.89 (d, 1H, J = 2.2 Hz), 5.13-5.12 (m, 1H), 3.23 (q, 2H, J = 7.0 Hz), 1.84 (d, 3H, J = 1.2 Hz),1.24 (s, 6H), 1.08 (t, 3H, J = 7.0 Hz), 0.94 (s, 9H), 0.17 (s, 6H) ppm.

^{13}C-NMR (126 MHz, DMSO-d_6): δ = 155.86, 144.41, 126.96, 126.43, 124.33, 116.13, 105.97, 101.83, 56.54, 37.43, 28.44, 25.60, 18.30, 17.98, 13.88, -4.39 ppm.

MALDI HRMS (m/z): calc. for $C_{20}H_{33}NOSi$ $[M+H]^+$ 332.24042, found 332.24043 (Δm = 0.00001, error 0.03 ppm).

Compound 4c:

Compound **4a** (13.5 g, 44.5 mmol, 1 eq) was dissolved in 50 mL dry MeOH. Pd/C (w_{Pd} = 5%) (11.51 g, 5.4 mmol Pd, 0.12 eq) was added and the suspension was stirred overnight under a hydrogen atmosphere. The mixture was filtered over celite and the solvent was removed under reduced pressure. Purification *via* column chromatography (cyclohexanes/EtOAc 50:1) yielded in **4c** as a brown solid.

Yield: 13.32 g (98%)

TLC: cyclohexane/DCM 1:2, R_f = 0.64

^{1}H-NMR (250 MHz, DMSO-d_6): δ = 6.87 (d, 1H, J = 8.0 Hz), 5.97-5.92 (m, 2H), 5.43 (bs, 1H), 2.80-2.63 (m, 1H), 1.68-1.61 (m, 1H), 1.22-1.17 (m, 4H), 1.15 (s, 3H), 1.06 (s, 3H), 0.93 (s, 9H), 0.14 (s, 6H) ppm.

MALDI HRMS (m/z): calc. for $C_{18}H_{31}NOSi$ $[M+H]^+$ 306.22477, found 306.22381 (Δm = 0.00096 error 3.13 ppm).

Compound 4d:

Compound **4c** (3.22 g, 10.5 mmol, 1 eq) and Iodoethane (2.54 mL, 31.6 mmol, 3 eq) were dissolved in 10 mL dry MeCN in a microwave vessel. Cs_2CO_3 (8.31 g, 22.5 mmol, 2.4 eq) was added and the resulting suspension was reacted for 7 h at 100 °C in a microwave. After cooling to room temperature the mixture was flushed with 50 mL DCM und washed with 50 mL dest. water. The aqueous layer was extracted with DCM four times, the organic layers were combined and the solvent was removed under reduced pressure. The crude product was purified *via* column chromatography (cyclohexane/EtOAc 20:1) to give **4d** as a brown oil.

Yield: 2.93 g (83%)

TLC: cyclohexane/EtOAc 20:1, R_f = 0.61

^{1}H-NMR (400 MHz, DMSO-d_6): δ = 6.88 (dd, 1H, J = 8.1, 0.9 Hz), 6.00 (dd, 1H, J = 8.1, 2.3 Hz), 5.94 (d, 1H, J = 2.3 Hz), 3.43-3.34 (m, 1H), 3.11-2.95 (m, 1H), 2.77-2.67 (m, 1H), 1.70 (dd, 1H, J = 12.9, 4.7 Hz), 1.27 (s, 1H), 1.20 (d, 3H, J = 6.6 Hz), 1.13-1.06 (m, 7H), 0.93 (s, 9H), 0.15 (s, 6H) ppm.

^{13}C-NMR (101 MHz, DMSO-d_6): δ = 154.11, 145.20, 126.22, 120.23, 105.91, 102.40, 53.88, 46.50, 29.22, 26.16, 25.61, 24.53, 20.08, 17.92, 14.66, 4.41 ppm.

MALDI HRMS (m/z): calc. for $C_{20}H_{35}NOSi$ $[M+H]^+$ 334.25607, found 334.25588 (Δm = 0.00019, error 0.57 ppm).

Compound 5a:

Compound **4a** (5.28 g, 17.4 mmol, 1 eq) was dissolved in 50 mL dry THF. 26 mL TBAF (1 M in THF, 26.0 mmol, 1.5 eq) and glacial AcOH (1.57 g, 1.49 mL, 26.1 mmol, 1.5 eq) were added and the mixture was stirred at room temperature overnight. 200 mL sat. NH_4Cl solution was added and the resulting mixture was extracted with DCM five times. The solvent was removed under reduced pressure and the crude product was purified *via* column chromatography (DCM/acetone) to give **5a** as a brown solid.

Yield: 3.25 g (99%)

TLC: DCM/acetone 3:1, R_f = 0.83

^{1}H-NMR (400 MHz, DMSO-d_6): δ = 8.91 (s, 1H), 6.72 (d, 1H, J = 8.2 Hz), 5.91 (d, 1H, J = 2.4 Hz), 5.88 (dd, 1H, J = 8.2, 2.4 Hz), 5.65 (s, 1H), 5.05 (s, 1H), 1.81 (d, 3H, J = 1.3 Hz), 1.15 (s, 6H) ppm.

^{13}C-NMR (101 MHz, DMSO-d_6): δ = 157.80, 145.49, 127.37, 124.87, 124.12, 112.70, 102.82, 98.99, 51.05, 30.88, 18.33 ppm.

MALDI HRMS (m/z): calc. for $C_{12}H_{15}NO$ $[M+H]^+$ 190.12264, found 190.12265 (Δm = 0.00001, error 0.05 ppm).

Compound 5b:

Compound **4b** (1.76 g, 5.3 mmol, 1 eq) was dissolved in 20 mL dry THF. 8 mL TBAF (1 M in THF, 8.0 mmol, 1.51 eq) and glacial AcOH (450 µL, 7.9 mmol, 1.48 eq) were added and the mixture was stirred at room temperature overnight. 100 mL sat. NH_4Cl solution was added and the resulting mixture was extracted with DCM five times. The solvent was removed under reduced pressure and the crude product was purified *via* column chromatography (cyclohexane/EtOAc 5:1) to give **5b** as a brown solid.

Yield: 1.06 g (92%)

TLC: cyclohexane/EtOAc 5:1, R_f = 0.40

^{1}H-NMR (400 MHz, DMSO-d_6): δ = 8.99 (s, 1H), 6.76 (d, 1H, J = 8.7 Hz), 5.94-5.90 (m, 2H), 5.05 (d, 1H, J = 1.2 Hz), 3.22 (q, 2H, J = 7.0), 1.82 (d, 3H, J = 1.1 Hz), 1.23 (s, 6H), 1.09 (t, 3H, J = 7.1 Hz) ppm.

^{13}C-NMR (101 MHz, DMSO-d_6): δ = 158.19, 144.60, 126.71, 125.74, 124.37, 114.12, 101.73, 97.47, 56.36, 37.49, 28.35, 18.36, 14.11 ppm.

MALDI HRMS (m/z): calc. for $C_{14}H_{19}NO$ $[M+H]^+$ 218.15394, found 218.15403 (Δm = 0.00009, error 0.41 ppm).

Compound 5c:

Compound **4c** (5.01 g, 16.4 mmol, 1 eq) was dissolved in 30 mL dry THF. 24.6 mL TBAF (1 M in THF, 24.6 mmol, 1.5 eq) and glacial AcOH (1.4 mL, 24.5 mmol, 1.5 eq) were added and the mixture was stirred at room temperature for 20 minutes. 150 mL sat. NH_4Cl solution was added and the resulting mixture was extracted with DCM four times. The solvent was removed under reduced pressure and the crude product was purified *via* column chromatography (cyclohexane/EtOAc 4:1) to give **5c** as a brown solid.

Yield: 2.90 g (92%)

TLC: cyclohexane/EtOAc 3:1, R_f = 0.25

^{1}H-NMR (400 MHz, DMSO-d_6): δ = 8.61 (s, 1H), 6.80 (d, 1H, J = 8.2 Hz), 5.90 (dd, 1H, J = 8.1, 2.4 Hz), 5.87 (d, 1H, J = 2.4 Hz), 5.28 (bs, 1H), 2.74-2.65 (m, 1H), 1.63 (dd, 1H, J = 12.6, 5.3 Hz), 1.20-1.13 (m, 7H), 1.05 (s, 3H) ppm.

^{13}C-NMR (101 MHz, DMSO-d_6): δ = 155.95, 145.26, 126.97, 115.08, 103.04, 99.91, 48.33, 44.70, 30.86, 27.32, 26.54, 20.62 ppm.

MALDI HRMS (m/z): calc. for $C_{12}H_{17}NO$ $[M+H]^+$ 192.13829, found 192.13747 (Δm = 0.00082, error 4.27 ppm).

Compound 5d:

Compound **4d** (4.55 g, 13.6 mmol, 1 eq) was dissolved in 25 mL dry THF. 20.5 mL TBAF (1 M in THF, 20.5 mmol, 1.51 eq) and glacial AcOH (1.2 mL, 21.0 mmol, 1.5 eq) were added and the mixture was stirred at room temperature for 20 minutes. 100 mL sat. NH_4Cl solution was added and the resulting mixture was extracted with DCM four times. The solvent was removed under reduced pressure and the crude product was purified *via* column chromatography (cyclohexane/EtOAc 4:1) to give **5d** as a brown solid.

Yield: 2.66 g (89%)

TLC: cyclohexane/EtOAc 3:1, R_f = 0.41

^{1}H-NMR (400 MHz, DMSO-d_6): δ = 8.68 (s, 1H), 6.80 (dd, 1H, J = 8.3, 1.0 Hz), 5.96-5.92 (m, 2H), 3.41-3.32 (m, 1H), 3.08-2.98 (m, 1H), 2.73-2.65 (m, 1H), 1.68 (dd, 1H, J = 12.8, 4.8 Hz), 1.34 (t, 1H, J = 12.7 Hz), 1.26 (s, 3H), 1.20-1.17 (m, 4H), 1.11-1.07 (m, 6H) ppm.

^{13}C-NMR (101 MHz, DMSO-d_6): δ = 156.35, 145.29, 126.22, 117.71, 101.81, 98.06, 53.74, 46.80, 29.36, 26.09, 24.32, 20.34, 14.96 ppm.

MALDI HRMS (m/z): calc. for $C_{14}H_{21}NO$ $[M+H]^+$ 220.16959, found 220.16946 (Δm = 0.00013, error 0.59 ppm).

Compound 6a:

Compound **5a** (2.90 g, 15.3 mmol, 1 eq) and sodium diethyl oxalacetate (4.79 g, 22.8 mmol, 1.5 eq) were dissolved in 15 mL dry EtOH in a microwave vessel. The mixture was heated to 100 °C for 3 hours under microwave irradiation. After cooling to room temperature the dark red solution was flushed with 100 mL DCM. and washed with 100 mL sat. NH_4Cl. The aqueous layer was extracted with DCM eight times. The organic layers were combined. The solvent was removed under reduced pressure and the crude product was purified *via* flash-chromatography (DCM/MeOH) to give **6a** as a dark red solid.

Yield: 1.52 g (32%)

TLC: cyclohexane/EtOAc 3:1, R_f = 0.33

^{1}H-NMR (500 MHz, DMSO-d_6): δ = 7.58 (s, 1H), 7.25 (s, 1H), 6.31 (s, 1H), 6.28 (s, 1H), 5.45-5.44 (m, 1H), 4.37 (q, 2H, J = 7.1 Hz), 1.92 (d, 3H, J = 1.1 Hz), 1.34 (t, 3H, J = 7.1 Hz), 1.27 (s, 6H) ppm.

^{13}C-NMR (126 MHz, DMSO-d_6): δ = 164.36, 160.19, 156.26, 148.45, 143.42, 129.30, 125.91, 120.78, 117.49, 108.88, 104.22, 97.15, 61.98, 52.21, 31.72, 18.06, 13.89 ppm.

MALDI HRMS (m/z): calc. for $C_{18}H_{19}NO_4$ $[M+H]^+$ 314.13869, found 314.13850 (Δm = 0.00019, error 0.60 ppm).

Compound 6b:

Compound **5b** (1.06 g, 4.9 mmol, 1 eq) and sodium diethyl oxalacetate (1.54 g, 7.3 mmol, 1.5 eq) were dissolved in 15 mL dry EtOH in a microwave vessel. The mixture was heated to 100 °C for 3 hours under microwave irradiation. After cooling to room temperature the dark red solution was flushed with 50 mL DCM. and washed with 50 mL sat. NH_4Cl. The aqueous layer was extracted with DCM four times. The organic layers were combined. The solvent was removed under reduced pressure and the crude product was purified *via* flash-chromatography (DCM/MeOH) followed by recrystallization from cyclohexane to give **6b** as a dark red solid.

Yield: 783 mg (47%)

TLC: cyclohexane/EtOAc 3:1, R_f = 0.47

^{1}H-NMR (500 MHz, $CDCl_3$): δ = 7.81 (s, 1H), 6.50 (s, 1H), 6.33 (s, 1H), 5.27 (d, 1H, J = 1.2 Hz), 4.41 (q, 2H, J = 7.1 Hz), 3.37 (q, 2H, J = 7.1 Hz), 1.99 (d, 3H, J = 1.2 Hz), 1.41 (t, 3H, J = 7.1 Hz), 1.37 (s, 6H) 1.21 (t, 3H, J = 7.1 Hz) ppm.

^{13}C-NMR (126 MHz, $CDCl_3$): δ = 164.88, 161.76, 157.22, 147.60, 142.80, 129.81, 126.61, 121.17, 120.18, 111.11, 105.04, 96.90, 62.11, 58.13, 38.87, 29.51, 18.83, 14.25, 13.55 ppm.

MALDI HRMS (m/z): calc. for $C_{20}H_{23}NO_4$ $[M+H]^+$ 342.16999, found 342.17004 (Δm = 0.00005, error 0.15 ppm).

Compound 6c:

Compound **5c** (2.90 g, 15.2 mmol, 1 eq) and sodium diethyl oxalacetate (4.80 g, 22.8 mmol, 1.5 eq) were dissolved in 15 mL dry EtOH in a microwave vessel. The mixture was heated to 100 °C for 2 hours under microwave irradiation. After cooling to room temperature the solvent was removed under reduced pressure and the crude product was purified *via* column-chromatography (DCM/MeOH 9:1) to give **6c** as a dark red solid.

Yield: 1.48 g (31%)

TLC: cyclohexane/EtOAc 3:1, R_f = 0.42

^{1}H-NMR (400 MHz, DMSO-d_6): δ = 7.70 (s, 1H), 7.04 (s, 1H), 6.36 (s, 1H), 6.25 (s, 1H), 4.42-4.34 (m, 2H), 2.90-2.80 (m, 1H), 1.77 (dd, 1H, J = 12.8, 4.0 Hz), 1.34 (t, 3H, J = 7.1 Hz), 1.39-1.27 (m, 3H), 1.24 (s, 3H), 1.15 (s, 3H) ppm.

^{13}C-NMR (101 MHz, DMSO-d_6): δ = 164.46, 160.41, 154.77, 148.92, 143.61, 123.97, 122.25, 108.48, 103.89, 97.80, 61.93, 49.28, 42.93, 30.37, 28.35, 36.56, 19.56, 13.89 ppm.

MALDI HRMS (m/z): calc. for $C_{18}H_{21}NO_4$ $[M+H]^+$ 316.15434, found 316.15429 (Δm = 0.00005, error 0.16 ppm).

Compound 6d:

Compound **5d** (2.66 g, 12.1 mmol, 1 eq) and sodium diethyl oxalacetate (3.83 g, 18.2 mmol, 1.5 eq) were dissolved in 15 mL dry EtOH in a microwave vessel. The mixture was heated to 100 °C for 3 hours under microwave irradiation. After cooling to room temperature the dark red solution was flushed with 50 mL DCM. and washed with 50 mL sat. NH_4Cl. The aqueous layer was extracted with DCM four times. The organic layers were combined. The solvent was removed under reduced pressure. The crude product was purified *via* flash-chromatography (DCM/MeOH) and recrystallized from cyclohexane to give **6d** as red crystals.

Yield: 1.75 g (42%)

^{1}H-NMR (500 MHz, $CDCl_3$): δ = 7.90 (d, 1H, J = 1.4 Hz), 6.50 (s, 1H), 6.44 (s, 1H), 4.42 (q, 2H, J = 7.1 Hz), 3.52-3.45 (m, 1H), 3.31-3.24 (m, 1H), 2.91-2.84 (m, 1H), 1.77 (dd, 1H, J = 13.1, 4.5 Hz), 1.56 (t, 1H, J = 13.1 Hz), 1.42 (t, 3H, 7.1 Hz), 1.37-1.35 (m, 6H), 1.24-1.21 (m, 6H) ppm.

^{13}C-NMR (126 MHz, $CDCl_3$): δ = 165.02, 162.06, 155.80, 148.64, 143.00, 125.82, 123.37, 111.02, 104.86, 97.68, 62.11, 55.62, 46.23, 39.55, 29.63, 27.04, 25.91, 19.96 14.29, 14.15 ppm.

MALDI HRMS (m/z): calc. for $C_{20}H_{25}NO_4$ $[M+H]^+$ 344.18564, found 344.18553 (Δm = 0.00011, error 0.15 ppm).

Compound 7a:

Compound **6a** (2.60 g, 8.3 mmol, 1 eq) was dissolved in 100 mL dry MeOH. $NaBH_4$ (1.57 g, 41.5 mmol, 5 eq) was added in small portions over 2 hours at room temperature. After additional stirring at room temperature for 2 hours, 100 mL HCl (1 M) was added. MeOH was removed under reduced pressure and the aqueous phase was extracted with DCM six times. The organic layers were combined and the solvent was removed under reduced pressure. The crude product was purified *via* column chromatography (cyclohexane/EtOAc 1:1) to give **7a** as a light-yellow solid.

Yield: 1.40 g (62%)

TLC: cyclohexane/EtOAc 1:1, R_f = 0.32

^{1}H-NMR (500 MHz, DMSO-d_6): δ = 7.09 (s, 1H), 6.91-6.90 (m, 1H), 5.47 (t, 1H, J = 5.8 Hz), 5.40-5.38 (m, 1H), 4.67 (dd, 2H, J = 5.6, 1.2 Hz), 1.95 (d, 3H, J = 1.2 Hz), 1.24 (s, 6H) ppm.

^{13}C-NMR (126 MHz, DMSO-d_6): δ = 160.96, 157.07, 155.08, 147.83, 128.84, 126.35, 118.47, 117.13, 105.97, 103.40, 97.00, 59.07, 51.86, 31.58, 18.22 ppm.

MALDI HRMS (m/z): calc. for $C_{16}H_{17}NO_3$ $[M+H]^+$ 272.12812, found 272.12848 (Δm = 0.00036, error 1.32 ppm).

Compound 7b:

Compound **6b** (786 mg, 2.3 mmol, 1 eq) was dissolved in 13 mL dry MeOH. $NaBH_4$ (347 mg, 9.2 mmol, 4 eq) was added in small portions over 3 hours at room temperature. After additional stirring at room temperature for 1 hour, 20 mL HCl (1 M) was added. MeOH was removed

under reduced pressure and the aqueous phase was extracted with EtOAc four times. The organic layers were combined and the solvent was removed under reduced pressure. The crude product was purified *via* column chromatography (cyclohexane/EtOAc 1:1) to give **7b** as a light-yellow solid.

Yield: 370 mg (54%)

TLC: cyclohexane/EtOAc 1:1, R_f = 0.34

^{1}H-NMR (500 MHz, DMSO-d$_6$): δ = 7.09 (s, 1H), 6.34 (s, 1H), 6.05 (s, 1H), 5.49 (t, 1H, J = 5.5 Hz), 5.39 (s, 1H), 4.69 (d, 2H, J = 4.7 Hz), 3.40 (q, 2H, J = 6.9 Hz), 1.95-1.94 (m, 3H), 1.33 (s, 6H), 1.13 (t, 3H, J = 6.9 Hz) ppm.

^{13}C-NMR (126 MHz, DMSO-d$_6$): δ = 161.03, 156.89, 155.47, 146.59, 129.84, 125.62, 118.88, 118.26, 105.43, 103.82, 95.97, 59.03, 57.51, 37.96, 28.94, 18.27, 13.30 ppm.

MALDI HRMS (m/z): calc. for $C_{18}H_{21}NO_3$ $[M+H]^+$ 300.15942, found 300.15963 (Δm = 0.00021, error 0.70 ppm).

Compound 7c:

Compound **6c** (1.48 g, 4.7 mmol, 1 eq) was dissolved in 30 mL dry MeOH. $NaBH_4$ (630 mg, 16.7 mmol, 3.5 eq) was added in small portions over 3 hours at room temperature. After additional stirring at room temperature for 1 hour, 50 mL HCl (1 M) was added. MeOH was removed under reduced pressure and the aqueous phase was extracted with EtOAc 5 times. The organic layers were combined and the solvent was removed under reduced pressure. The crude product was purified *via* column chromatography (cyclohexane/EtOAc 3:1) to give **7c** as a light-yellow solid.

Yield: 480 mg (37%)

TLC: cyclohexane/EtOAc 1:1, R_f = 0.25

^{1}H-NMR (500 MHz, DMSO-d$_6$): δ = 7.27 (s, 1H), 6.70 (s, 1H), 6.33 (s, 1H), 6.01 (t, 1H, J = 1.2 Hz), 5.46 (t, 1H, J = 5.6 Hz), 4.71-4.62 (m, 2H), 2.87-2.79 (m, 1H), 1.76 (ddd, 1H, J = 12.8, 5.0, 1.4 Hz), 1.32 (d, 3H, J = 6.6 Hz), 1.23 (s, 3H), 1.14 (s, 3H) ppm.

^{13}C-NMR (126 MHz, DMSO-d$_6$): δ = 161.70, 157.46, 154.16, 148.69, 122.14, 122.02, 106.26, 103.80, 98.17, 59.58, 49.46, 43.81, 30.97, 28.67, 27.18, 20.39 ppm.

MALDI HRMS (m/z): calc. for $C_{16}H_{19}NO_3$ $[M+H]^+$ 274.14377 found 274.14370 (Δm = 0.00007, error 0.26 ppm).

Compound 7d:

Compound **6d** (1.75 g, 5.1 mmol, 1 eq) was dissolved in 45 mL dry MeOH. $NaBH_4$ (675 mg, 17.8 mmol, 3.5 eq) was added in small portions over 3 hours at room temperature. After additional stirring at room temperature for 1 hour, 50 mL HCl (1 M) was added. MeOH was removed under reduced pressure and the aqueous phase was extracted with EtOAc 4 times. The organic layers were combined and the solvent was removed under reduced pressure. The crude product was purified *via* column chromatography (cyclohexane/EtOAc 3:1) to give **7d** as a light-yellow solid.

Yield: 600 mg (39%)

TLC: cyclohexane/EtOAc 1:1, R_f = 0.40

^{1}H-NMR (500 MHz, DMSO-d_6): δ = 7.24 (d, 1H, J = 1.2 Hz), 6.38 (s, 1H), 6.05 (t, 1H, J = 1.2 Hz), 5.48 (t, 1H, J = 5.6 Hz), 4.69-4.67 (m, 2H), 3.56-3.49 (m, 1H), 3.29-3.22 (m, 1H), 2.84-2.77 (m, 1H), 1.81 (dd, 1H, J = 13.1, 4.5 Hz), 1.42 (t, 1H, J = 13.0 Hz), 1.33 (s, 3H), 1.31 (d, 3H, J = 6.6 Hz), 1.19 (s, 3H), 1.14 (t, 3H, J = 6.9 Hz) ppm.

^{13}C-NMR (126 MHz, DMSO-d_6): δ = 161.26, 156.80, 154.06, 147.61, 124.62, 120.41, 105.28, 103.92, 96.56, 59.07, 54.93, 45.53, 28.94, 26.38, 25.25, 19.71, 13.84 ppm.

MALDI HRMS (m/z): calc. for $C_{18}H_{23}NO_3$ $[M+H]^+$ 302.17507 found 302.17509 (Δm = 0.00002, error 0.07 ppm).

Compound 8a:

Carbonyldiimidazole (478 mg, 3.0 mmol, 2 eq) was suspended in 4 mL dry DCM in a microwave vessel. Compound **7a** (400 mg, 1.5 mmol, 1 eq) was added in small portions under continuous stirring. The vessel was flushed with argon and the mixture was reacted at 45 °C under microwave irradiation for 45 min. The solvent was removed under reduced pressure and the crude product was purified *via* column chromatography (cyclohexanes/EtOAc 2:1 → 1:2) to give **8a** as a yellow solid.

Yield: 446 mg (83%)

TLC: cyclohexane/EtOAc 1:2, R_f = 0.49

^{1}H-NMR (600 MHz, DMSO-d_6): δ = 8.38 (s, 1H), 7.70 (t, 1H, J = 1.4 Hz), 7.18 (s, 1H), 7.12-7.08 (m, 2H), 6.31 (s, 1H), 6.13 (s, 1H), 5.66-5.65 (m, 2H), 5.43-5.41 (m, 1H), 1.96-1.95 (m, 3H), 1.26 (s, 6H) ppm.

MALDI HRMS (m/z): calc. for $C_{20}H_{19}N_3O_4$ $[M+H]^+$ 366.14483, found 366.14475 (Δm = 0.00008, error 0.22 ppm).

Compound 8b:

Carbonyldiimidazole (108 mg, 0.67 mmol, 2 eq) was suspended in 3 mL dry DCM in a microwave vessel. Compound **7b** (100 mg, 0.33 mmol, 1 eq) was added in small portions under continuous stirring. The vessel was flushed with argon and the mixture was reacted at 45 °C under microwave irradiation for 45 min. The solvent was removed under reduced pressure and the crude product was purified *via* column chromatography (cyclohexanes/EtOAc 2:1 → 1:2) to give **8b** as a yellow solid.

Yield: 116 mg (89%)

TLC: cyclohexane/EtOAc 1:2, R_f = 0.44

^{1}H-NMR (400 MHz, DMSO-d_6): δ = 8.38 (s, 1H), 7.70 (t, 1H, J = 1.3 Hz), 7.18 (s, 1H), 7.12-7.10 (m, 1H), 6.39 (s, 1H), 6.18 (s, 1H), 5.66-5.65 (m, 2H), 5.67 (s, 1H), 5.43-5.41 (m, 1H), 3.43 (q, 2H, J = 7.0 Hz), 1.96-1.94 (m, 3H), 1.35 (s, 6H), 1.14 (t, 3H, J = 7.0 Hz) ppm.

MALDI HRMS (m/z): calc. for $C_{22}H_{23}N_3O_4$ $[M+H]^+$ 394.17613, found 394.17553 (Δm = 0.00060, error 1.52 ppm).

Compound 8c:

Carbonyldiimidazole (107 mg, 0.66 mmol, 2 eq) was suspended in 3 mL dry DCM in a microwave vessel. Compound **7c** (90 mg, 0.33 mmol, 1 eq) was added in small portions under continuous stirring. The vessel was flushed with argon and the mixture was reacted at 45 °C under microwave irradiation for 45 min. The solvent was removed under reduced pressure and the crude product was purified *via* column chromatography (cyclohexanes/EtOAc 2:1 → 1:2) to give **8c** as a yellow solid.

Yield: 95 mg (79%)

TLC: cyclohexane/EtOAc 1:1, R_f = 0.20

^{1}H-NMR (500 MHz, DMSO-d_6): δ = 8.37-8.36 (m, 1H), 7.69 (t, 1H, J = 1.4 Hz), 7.37 (s, 1H), 7.11 (dd, 1H, J = 1.5, 0.8 Hz), 6.88 (s, 1H), 6.36 (s, 1H), 6.11 (s, 1H), 5.68-5.60 (m, 2H), 2.89-2.81 (m, 1H), 1.80-1.74 (m, 1H), 1.33 (d, 3H, J = 6.6 Hz), 1.25-1.22 (m, 4H), 1.15 (s, 3H) ppm.

^{13}C-NMR (126 MHz, DMSO-d_6): δ = 160.59, 153.96, 149.09, 148.71, 147.95, 137.45, 130.52, 122.23, 121.98, 117.67, 105.20, 105.14, 97.77, 65.01, 49.12, 43.17, 30.44, 28.26, 26.72, 19.85 ppm.

MALDI HRMS (m/z): calc. for $C_{20}H_{21}N_3O_4$ $[M+H]^+$ 368.16048, found 368.16056 (Δm = 0.00008, error 0.22 ppm).

Compound 8d:

Carbonyldiimidazole (54 mg, 0.33 mmol, 2 eq) was suspended in 3 mL dry DCM in a microwave vessel. Compound **7d** (50 mg, 0.17 mmol, 1 eq) was added in small portions under continuous stirring. The vessel was flushed with argon and the mixture was reacted at 45 °C under microwave irradiation for 45 min. The solvent was removed under reduced pressure and the crude product was purified *via* flash chromatography (cyclohexanes/EtOAc) to give **8d** as a yellow solid.

Yield: 55 mg (83%)

TLC: cyclohexane/EtOAc 1:2, R_f = 0.50

^{1}H-NMR (600 MHz, DMSO-d_6): δ = 8.37 (s, 1H), 7.69 (s, 1H), 7.33 (s, 1H), 7.11 (s, 1H), 6.42 (s, 1H), 6.17 (s, 1H), 5.68-5.62 (m, 2H), 3.56-3.50 (m, 1H), 3.30-3.24 (m, 1H), 2.83-2.80 (m, 1H), 1.81 (dd, 1H, J = 13.1, 4.4 Hz), 1.42 (t, 1H, J = 13.1 Hz), 1.33 (s, 3H), 1.30 (d, 3H, J = 6.5 Hz), 1.19 (s, 3H), 1.14 (t, 3H, J = 6.9 Hz) ppm.

MALDI HRMS (m/z): calc. for $C_{22}H_{25}N_3O_4$ $[M]^{radikal}$ 395.18396, found 395.18462 (Δm = 0.00066, error 1.67 ppm).

Compound 9a:

5-Hydroxytryptamine hydrochloride (116 mg, 0.55 mmol, 2 eq) and Et_3N (138 mg, 191 µL, 1.37 mmol, 5 eq) were dissolved in 3 mL dry DMF in a microwave vessel. Compound **8a** (100 mg, 0.27 mmol, 1 eq) was added in small portions under continuous stirring. The vessel was flushed with argon and the mixture was reacted at 50 °C under microwave irradiation for 45 min. The crude reaction mixture was cooled to room temperature and directly purified *via* flash chromatography (cyclohexanes/EtOAc) without prior solvent removal. After evaporating the solvents under reduced pressure, **9a** was obtained as yellow solid.

Yield: 84 mg (65%)

TLC: cyclohexane/EtOAc 1:2, R_f = 0.68

^{1}H-NMR (500 MHz, DMSO-d_6): δ = 10.49-10.47 (m, 1H), 8.57 (s, 1H), 7.62 (t, 1H, J = 8.0 Hz), 7.13-7.11 (m, 2H), 7.05 (d, 1H, J = 2.1 Hz), 7.02-7.01 (m, 1H, J = 1.3 Hz), 6.83 (d, 1H, J = 2.1 Hz), 6.59 (dd, 1H, J = 8.6, 2.3 Hz), 6.30 (s, 1H), 5.94 (s, 1H), 5.41-5.40 (m, 1H), 5.24 (s, 2H), 3.27 (q, 2H, J = 6.0 Hz), 2.77 (t, 2H, J = 7.8 Hz), 1.96 (d, 3H, J = 0.7 Hz), 1.26 (s, 6H) ppm.

^{13}C-NMR (126 MHz, DMSO-d_6): δ = 160.59, 155.47, 155.27, 152.19, 150.20, 148.13, 130.80, 128.99, 127.94, 126.27, 123.11, 118.80, 117.28, 111.66, 111.29, 110.48, 105.60, 104.08, 102.13, 97.02, 61.01, 51.99, 41.16, 61.64, 25.60, 18.20 ppm.

MALDI HRMS (m/z): calc. for $C_{27}H_{27}N_3O_5$ $[M+H]^+$ 496.18429, found 496.18308 (Δm = 0.00121, error 2.44 ppm).

Compound 9b:

5-Hydroxytryptamine hydrochloride (108 mg, 0.51 mmol, 2 eq) and Et_3N (164 mg, 1.27 mmol, 5 eq) were dissolved in 4 mL dry DMF in a microwave vessel. Compound **8b** (100 mg, 0.25 mmol, 1 eq) was added in small portions under continuous stirring. The vessel was flushed with argon and the mixture was reacted at 50 °C under microwave irradiation for 45 min. After cooling to room temperature, the solution was flushed with 30 mL DCM and washed with 20 mL water. The aqueous phase was extracted with DCM two times. The organic phases were combined and the solvent was removed under reduced pressure. The crude product was purified *via* flash chromatography (DCM/MeOH) to give **9b** as yellow solid.

Yield: 87 mg (69%)

TLC: DCM/MeOH 9:1, R_f = 0.61

^{1}H-NMR (600 MHz, DMSO-d_6): δ = 10.49 (s, 1H), 8.59 (s, 1H), 7.63 (t, 1H, J = 5.8 Hz), 7.13-7.11 (m, 2H), 7.05-7.04 (m, 1H), 6.83 (d, 1H, J = 1.8 Hz), 6.59 (dd, 1H, J = 8.6, 2.1 Hz), 6.37 (s, 1H), 5.98 (s, 1H), 5.41 (s, 1H), 5.25 (s, 2H), 3.42 (q, 2H, J = 7.0 Hz), 3.28 (q, 2H, J = 8.0 Hz), 2.76 (t, 2H, J = 8.2 Hz), 1.96 (s, 3H), 1.13 (t, 3H, J = 6.6 Hz) ppm.

^{13}C-NMR (126 MHz, DMSO-d_6): δ = 160.65, 155.67, 155.45, 151.96, 150.19, 146.87, 130.79, 129.99, 127.93, 125.63, 123.10, 119.05, 118.56, 111.65, 111.27, 110.46, 105.10, 104.61, 102.12, 96.04, 60.97, 57.65, 28.99, 25.99, 18.24, 13.26 ppm.

MALDI HRMS (m/z): calc. for $C_{29}H_{31}N_3O_5$ $[M+Na]^+$ 524.21559, found 524.21430 (Δm = 0.00129, error 2.46 ppm).

Compound 9c:

5-Hydroxytryptamine hydrochloride (98 mg, 0.46 mmol, 2 eq) and Et_3N (150 mg, 1.16 mmol, 5 eq) were dissolved in 4 mL dry DMF in a microwave vessel. Compound **8c** (85 mg, 0.25 mmol, 1 eq) was added in small portions under continuous stirring. The vessel was flushed with argon and the mixture was reacted at 50 °C under microwave irradiation for 45 min. After cooling to room temperature, the solution was flushed with 30 mL DCM and washed with 20 mL water. The aqueous phase was extracted with DCM two times. The organic phases were combined and the solvent was removed under reduced pressure. The crude product was purified *via* flash chromatography (DCM/MeOH) to give **9c** as yellow solid.

Yield: 87 mg (69%)

TLC: cyclohexane/EtOAc 1:2, R_f = 0.50

^{1}H-NMR (600 MHz, DMSO-d_6): δ = 10.49 (s, 1H), 8.59 (s, 1H), 7.63 (t, 1H, J = 5.6 Hz), 7.29 (s, 1H), 7.12 (d, 1H, J = 8.6 Hz), 7.05 (s, 1H), 6.83 (s, 2H), 6.59 (dd, 1H, J = 8.6, 1.9 Hz), 6.34 (s, 1H), 5.91 (s, 1H), 5.24 (q, 2H, J = 15.6 Hz), 3.30-3.25 (m, 2H), 2.85-2.81 (m, 1H), 2.77-2.74 (m, 2H), 1.76 (dd, 1H, J = 12.8, 4.8 Hz), 1.33 (d, 3H, J = 6.6 Hz), 1.29-1.26 (m, 1H), 1.23 (s, 3H), 1.14 (s, 3H) ppm.

^{13}C-NMR (126 MHz, DMSO-d_6): δ = 160.83, 155.48, 153.85, 152.17, 150.19, 148.54, 130.80, 127.83, 123.09, 212.96, 121.82, 111.65, 111.27, 110.47, 105.37, 103.95, 102.12, 97.71, 60.93, 54.90, 49.07, 43.22, 41.16, 30.94, 30.45, 28.24, 26.70, 25.58, 22.05, 19.80, 13.95 ppm.

MALDI HRMS (m/z): calc. for $C_{27}H_{29}N_3O_5$ $[M+Na]^+$ 498.19994, found 498.19894 (Δm = 0.00100, error 2.01 ppm).

Compound 9d:

5-Hydroxytryptamine hydrochloride (54 mg, 0.25 mmol, 2 eq) and Et_3N (98 mg, 0.76 mmol, 6 eq) were dissolved in 4 mL dry DMF in a microwave vessel. Compound **8d** (50 mg, 0.13 mmol, 1 eq) was added in small portions under continuous stirring. The vessel was flushed with argon and the mixture was reacted at 50 °C under microwave irradiation for 45 min. After cooling to room temperature, the solution was flushed with 30 mL DCM and washed with 20 mL water. The aqueous phase was extracted with DCM two times. The organic phases were combined and the solvent was removed under reduced pressure. The crude product was purified *via* flash chromatography (DCM/MeOH) to. After removing the solvent, the resulting oil was dissolved in 2 mL DCM and 15 mL *n*-hexane (precooled to -20 °C) was added. The precipitant **9d** was filtered and dried in vacuo.

Yield: 49 mg (77%)

TLC: cyclohexane/EtOAc 1:2, R_f = 0.56

^{1}H-NMR (500 MHz, DMSO-d_6): δ = 10.48 (s, 1H), 8.57 (s, 1H), 7.61 (t, 1H, J = 5.7 Hz), 7.26 (s, 1H), 7.12 (d, 1H, J = 8.6 Hz), 7.05 (d, 1H, J = 2.1 Hz), 6.83 (d, 1H, J = 2.1 Hz), 6.59 (dd, 1H, J = 8.6, 2.3 Hz), 6.41 (s, 1H), 5.97 (s, 1H), 5.29-5.22 (m, 2H), 3.57-3.50 (m, 1H), 3.30-3.26 (m, 3H), 2.84-2.75 (m, 3H), 1.82 (dd, 1H, J = 13.1, 4.4 Hz), 1.42 (t, 1H, J = 13.0 Hz), 1.34 (s, 3H), 1.32 (d, 3H, J = 6.5 Hz), 1.20 (s, 3H), 1.14 (t, 3H, J = 6.8 Hz) ppm.

^{13}C-NMR (126 MHz, DMSO-d_6): δ = 161.37, 155.95, 154.74, 152.45, 150.67, 148.40, 131.28, 128.32, 125.42, 123.58, 121.18, 112.13, 111.75, 110.95, 105.41, 105.06, 102.60, 97.15, 61.40, 55.56, 45.92, 41.64, 39.18, 29.39, 26.88, 26.06, 25.80, 20.09, 14.27 ppm.

MALDI HRMS (m/z): calc. for $C_{29}H_{33}N_3O_5$ $[M+H]^+$ 504.24930, found 504.24854 (Δm = 0.00076, error 1.51 ppm).

Compound 10:

Carbonyldiimidazole (393 mg, 2.4 mmol, 2 eq) was suspended in 3 mL dry DCM in a microwave vessel. Compound **9** (300 mg, 1.2 mmol, 1 eq, prepared according to literature[1]) was added in small portions under continuous stirring. The vessel was flushed with argon and the mixture was reacted at 45 °C under microwave irradiation for 45 min. The solvent was removed under reduced pressure and the crude product was purified *via* flash chromatography (cyclohexanes/EtOAc) to give **10** as a yellow solid.

Yield: 320 mg (77%)

TLC: cyclohexane/EtOAc 1:2, R_f = 0.39

^{1}H-NMR (250 MHz, DMSO-d_6): δ = 8.38 (s, 1H), 7.70 (t, 1H, J = 1.4 Hz), 7.56 (d, 1H, J = 9.0 Hz), 7.12-7.10 (m 1H), 6.71 (dd, 1H, J = 9.0, 2.5 Hz), 6.56 (d, 1H, J = 2.5 Hz), 6.20 (s, 1H), 5.62 (s, 1H), 3.44 (q, 4H, J = 7.0 Hz), 1.12 (t, 6H, J = 7.0 Hz) ppm.

Compound 11:

5-Hydroxytryptamine hydrochloride (125 mg, 0.59 mmol, 2 eq) and Et_3N (148 mg, 1.46 mmol, 5 eq) were dissolved in 2 mL dry DMF in a microwave vessel. Compound **10** (100 mg, 0.29 mmol, 1 eq) was added in small portions under continuous stirring. The vessel was flushed with argon and the mixture was reacted at 50 °C under microwave irradiation for 45 min. After cooling to room temperature, the solution was flushed with 30 mL DCM and washed with 20 mL water. The aqueous phase was extracted with DCM two times. The organic phases were combined and the solvent was removed under reduced pressure. The crude product was purified *via* flash chromatography (cyclohexane/EtOAc) to give **11** as yellow solid.

Yield: 90 mg (68%)

TLC: cyclohexane/EtOAc 1:2, R_f = 0.50

^{1}H-NMR (500 MHz, DMSO-d_6): δ = 10.48 (s, 1H), 8.58 (s, 1H), 7.62 (t, 1H, J = 5.7 Hz), 7.45 (d, 1H, J = 9.0 Hz), 7.12 (d, 1H, J = 8.6 Hz), 7.05 (d, 1H, J = 2.0 Hz), 6.83 (d, 1H, J = 2.0 Hz), 6.69 (dd, 1H, J = 9.1, 2.4 Hz), 6.59 (dd, 1H, J = 8.6, 2.3 Hz), 6.54 (d, 1H, J = 2.4 Hz), 5.98 (s, 1H), 5.23 (s, 2H), 3.42 (q, 4H, J = 6.9 Hz), 3.30-3.26 (m, 2H), 2.77 (t, 2H, J = 7.6 Hz), 1.12 (t, 6H, J = 7.0 Hz) ppm.

^{13}C-NMR (126 MHz, DMSO-d_6): δ = 160.77, 155.77, 155.45, 152.00, 150.45, 150.20, 130.81, 127.87, 125.34, 123.13 111.67, 111.28, 110.50, 108.78, 105.31, 104.52, 102.14, 96.85, 60.88, 43.99, 41.17, 25.57, 12.31 ppm.

MALDI HRMS (m/z): calc. for $C_{25}H_{27}N_3O_5$ $[M+H]^+$ 450.20235, found 450.20124 (Δm = 0.00111, error 2.50 ppm).

Two-photon excitation fluorescence experiments

For the two-photon excitation fluorescence (TPEF) measurements we used a tunable Ti:Sa laser (Tsunami, Spectra-Physics, USA) with a pulse duration of 150 fs and a 80 MHz repetition rate. The excitation was adjusted to an average energy of 300 mW. The pulses were then tightly focused onto the sample compartment. The TPEF-signal was coupled into a spectrograph (SpectraPro 300i, Acton Research Corp., USA), which is equipped with a CCD-camera (EEV 400_1340F, Roper Scientific, USA). To obtain the two-photon absorption spectrum we determined the two-photon absorption action cross sections in the range of 770-870 nm. We used coumarin307 as reference, using the following equation[2–5]

$$\phi_F(X)\,\sigma_2(X) = \sigma_2(R)\cdot\phi_F(R)\frac{I_F(X)\cdot c(R)\cdot\eta(R)}{I_F(R)\cdot c(X)\cdot\eta(X)} \quad (1)$$

where σ_2= two-photon absorption cross section, ϕ_F=fluorescence quantum yield, X=sample, R= reference, I_F= fluorescence intensity, c=concentration, η=refractive index of the solvent. We furthermore assumed the one-photon fluorescence quantum yield to be equal to the two-photon fluorescence quantum yield.[2,3] The values for the reference compound were taken from *Xu et al.*[2] The concentrations for the samples, as well as the coumarin307 reference were adjusted to 100 µM.

Time-correlated single photon counting experiments

The fluorescence decays of the compounds **7a-d** and **9a-d** were determined by the time-correlated single photon counting (TCSPC) technique. Our home-built TCSPC setup is composed of a single-photon detection photomultiplier tube (PMA-C 182 M, PicoQuant, Germany) and a PCIe card (TimeHarp 260 PICO Single, PicoQuant) for sub-ns data processing. Pulsed orthogonal excitation of the samples was achieved by a pulsed LED (PLS360, PicoQuant) with a peak wavelength of 360 nm and a FWHM < 800 ps. Deconvolution with the IRF and multi-exponential fitting of the temporal traces was performed with FluoFit Pro 4.6 (PicoQuant) based on the following equation[6]

$$\boldsymbol{I(t)} = \int_{-\infty}^{t} \boldsymbol{IRF(t')} \sum_{i=1}^{n} \boldsymbol{A_i e^{-\frac{t-t_i}{\tau_i}} dt'} \quad (2)$$

The samples were measured in 4 × 10 mm quartz glass cuvettes with an OD of ~0.1 on 10 mm optical pathlength.

Femtosecond UV/vis-pump-probe experiments

The time-resolved transient absorption measurements were performed using a home-built pump-probe setup. A Ti:Sa chirped pulse regenerative amplifier (MXR-CPA-iSeries, Clark-MXR Inc., USA) with a central output wavelength of 775 nm, a 1 kHz repetition rate, and a pulse width of 150 fs was used as the fs-laser source. The fundamental was split for pump and probe pulse generation. Pump pulses at a central wavelength of 388 nm were generated in a second harmonic generation (SHG). The temporal FWHM of the final pump pulses was determined to be ~120-130 fs. The excitation energy was set to 90 nJ/pulse at the sample position. The supercontinuum for the probe pulses was generated by focusing the fundamental in a constantly moving CaF_2 window of 5 mm thickness, leading to stable white light in the range of 375-740 nm. The white light was then split and guided through the sample and the reference arm of the detection setup. Each arm makes use of a spectrograph (Multimode, AMKO, Germany), which is equipped with two gratings (300 nm/ 500 nm blaze, 600/1200 grooves per mm), a photodiode array (S8865-64, Hamamatsu Photonics, Japan) and a corresponding driver circuit (C9118, Hamamatsu Photonics, Japan). The signals were digitized by a 16 bits data acquisition card (NI-PCI-6110, National Instruments, USA). The pump and probe pulses were set to the magic angle configuration at 54.7° to account for anisotropic effects. The sample-compartment was constantly moved to minimize sample degradation.

Kinetic analysis of the ultrafast spectroscopic data

The analysis of our experimental data was done by OPTIMUS.[7] For the ultrafast TA measurements, we used the lifetime distribution analysis (LDA). Within this method a quasi-continuous sum of exponentials is used to allow a model independent analysis of the data. The pre-exponential factors of a set amount of exponential functions, in this case 100, with fixed and equally distributed lifetimes were determined and plotted in a contour representation (lifetime density map, LDM). For a further read on the methodology see (www.optimusfit.org).

<u>Supplementary Data</u>

<u>Ultrafast Transient Absorption Data</u>

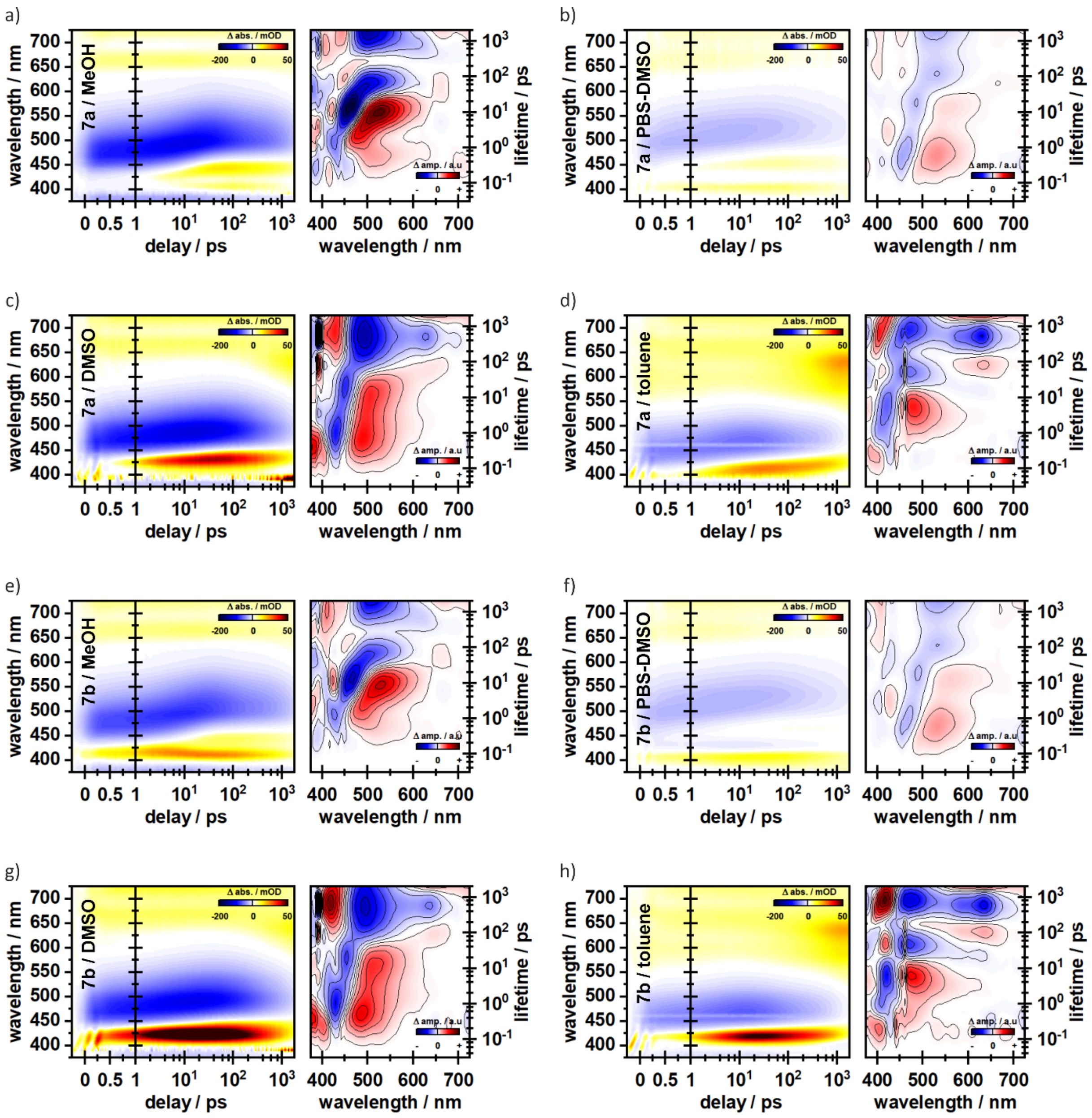

Figure S1: Transient absorption data and the corresponding lifetime density analysis of compound 7a (a-d) and 7b (e-h) in different solvents.

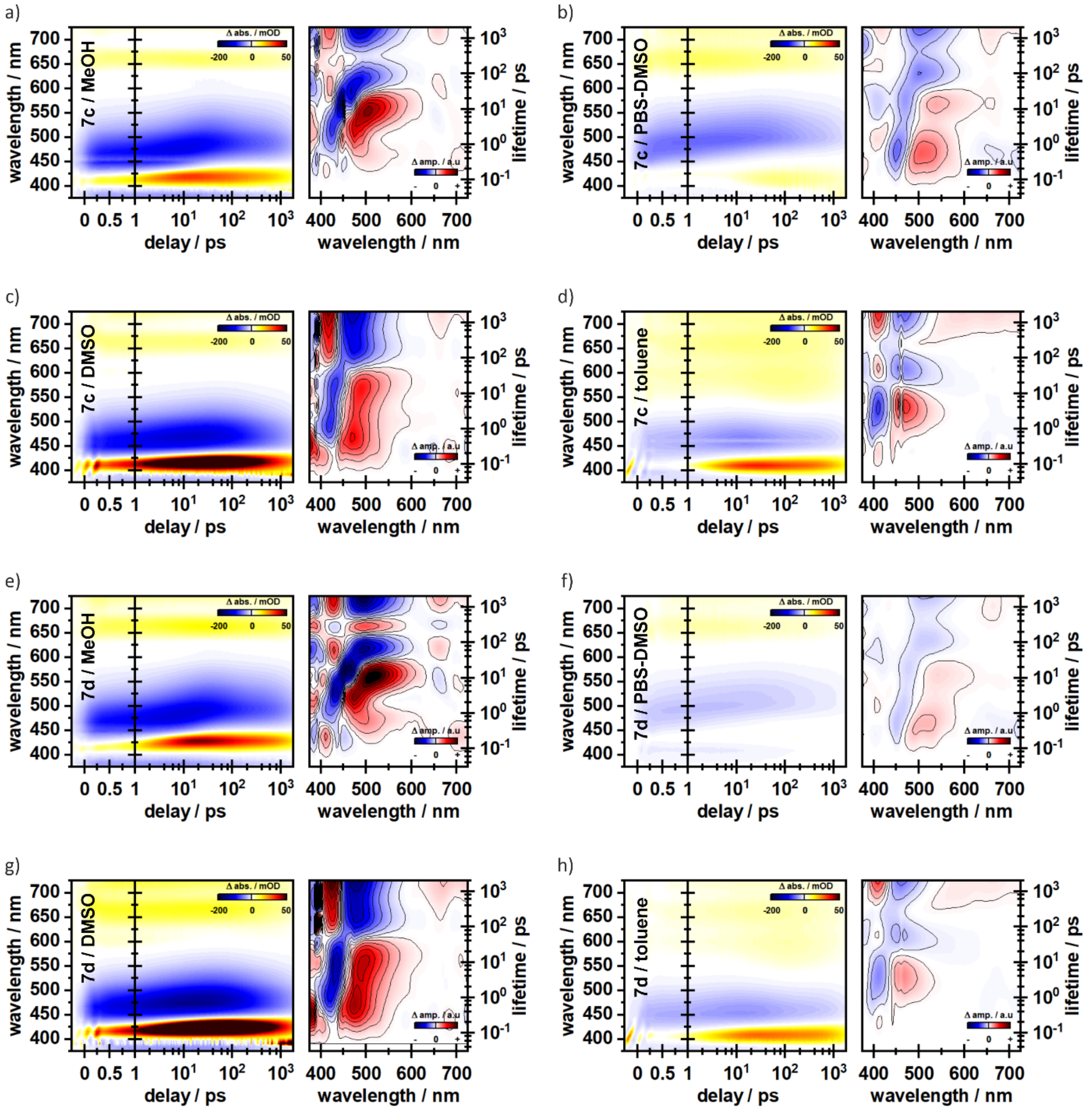

Figure S2: Transient absorption data and the corresponding lifetime density analysis of compound 7c (a-d) and 7d (e-h) in different solvents.

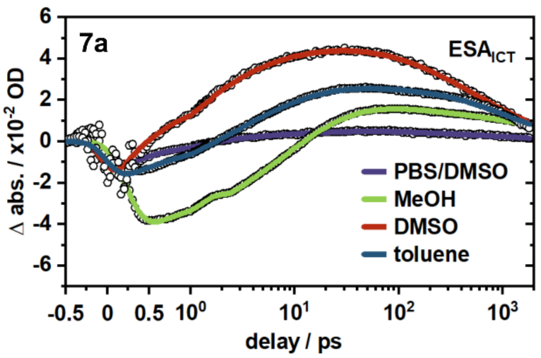

Figure S3: Transients of the ESA$_{ICT}$ signal for compound 7a in different solvents. Note that for each solvent, except the PBS/DMSO mixture, a significant rise occurs on the 1-100 ps timescale. This indicates that the CT state becomes more populated over time.

Dynamic Stokes' Shifts – Solvent Dependence

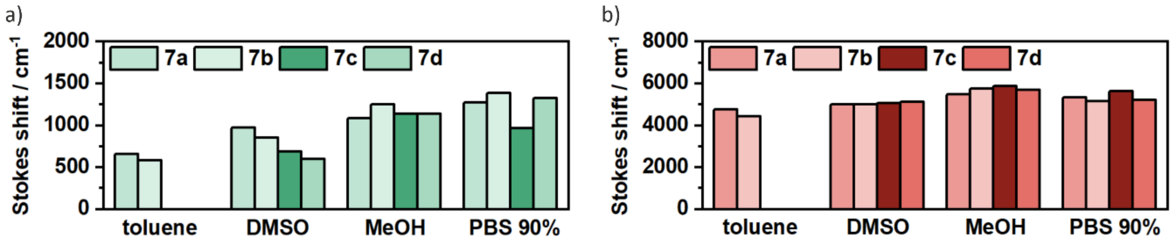

Figure S4: Stokes' shifts derived from the TA data. a) Difference of the SE bands at 0.5 ps and 100 ps, describing the dynamic Stokes' shift due to solvent relaxation. b) Difference of the ground state absorption and the SE band at 100 ps, describing the overall Stokes' shifts, which are similar in trend compared to the ones determined in steady state experiments (Figure 4c). Note that in toluene the SE signatures (at 100 ps) of compounds 7c and 7d are significantly overlapped with ESA bands. We therefore left out the Stokes' shifts for compound 7c and 7d in toluene.

To obtain the Stokes' shifts from the ultrafast TA data, we extracted the transient spectra at 0.5 ps and 100 ps, respectively. The spectra were then transformed into the frequency domain (given in wavenumbers) according to the Jacobian transformation.[8] This also included a scaling of the amplitudes (which is then indicated as corr. Δ abs. in Figures 5c and 5d). The Stokes' shifts were roughly determined by the wavenumber difference of the SE signals at 0.5 ps and 100 ps (Figure S4a) or the ground state absorption and the SE signal at 100 ps (Figure S4b).

Lifetime density analysis of 7a-d and 9a-d

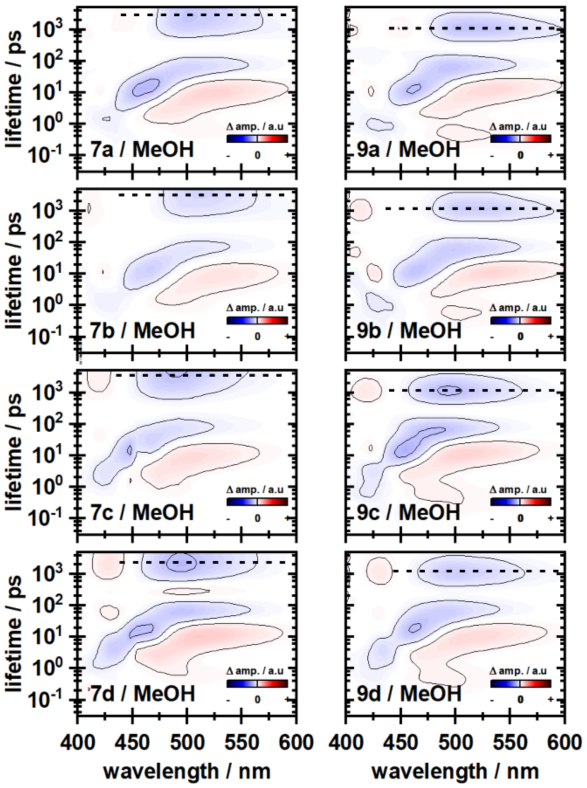

Figure S5: Lifetime density analysis (LDA) of compounds 7a-d and 9a-d in MeOH in the spectral range of the stimulated emission. Compounds 9a-d show a faster decay of the excited state (indicated by the dashed lines) in comparison to compounds 7a-d. The earlier lifetimes remain similar.

Lifetimes obtained by TCSPC

Table S1 Fluorescence decay lifetimes of compounds 7a-d and 9a-d obtained by TCSPC measurements. A similar trend as in the ultrafast measurements (Figure S5) is observed with a significant decrease of excited state lifetime in 9a-d.

	**7a**	**7b**	**7c**	**7d**
**τ_{avg} [ns]**	4.61	4.78	5.25	5.39
	**9a**	**9b**	**9c**	**9d**
**τ_{avg} [ns]**	2.05	2.63	2.36	2.28

Determination of quantum yields

Three stock solutions of each compound **9a-d** and **11** were prepared by diluting approx. 1.0 mg in MeOH/PBS (1:1). For photolysis 50 µL were taken and irradiated at nine different irradiation times (t = 0 s, 5 s, 10 s, 20 s, 45 s, 90 s, 180 s, 360 s, 720 s) with a 405 nm LED (*Thorlabs*), resulting in 27 differently irradiated solutions for each compound. The photon flux for the setup was determined with a fulgide-derivative – as described in literature.[9] The photolysis was analyzed via RP-HPLC (*Agilent 1200*) as the

ratio of the peak areas of starting material and uridine as internal standard – as described in literature.[10]

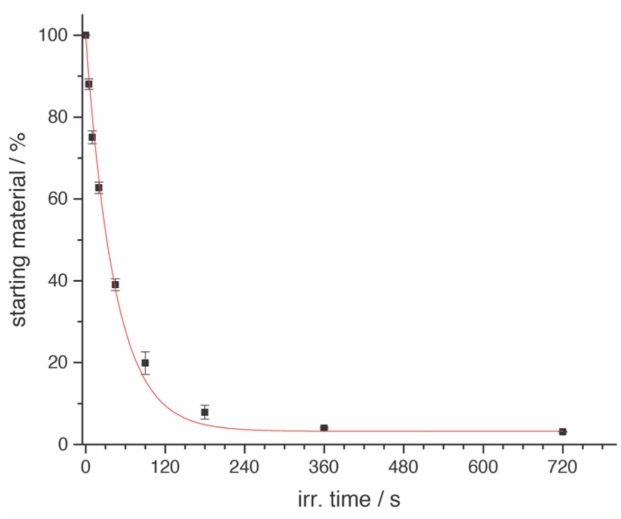

Figure S6: Photolysis curve of compound **9c** in PBS/MeOH (1:1).

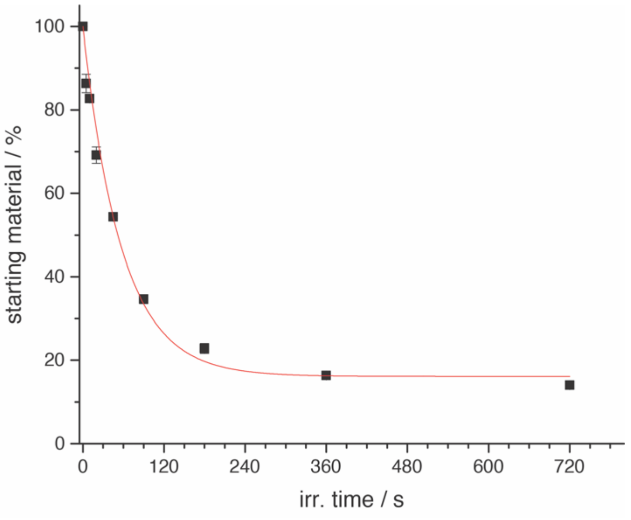

Figure S7: Photolysis curve of compound **9d** in PBS/MeOH (1:1).

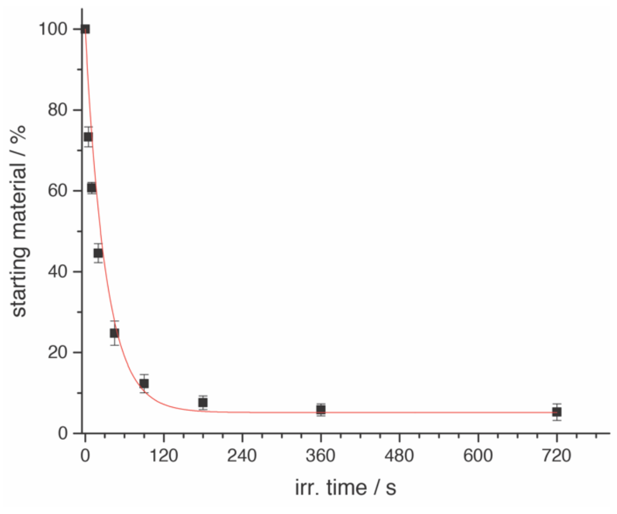

Figure S8: Photolysis curve of compound **9a** in PBS/MeOH (1:1).

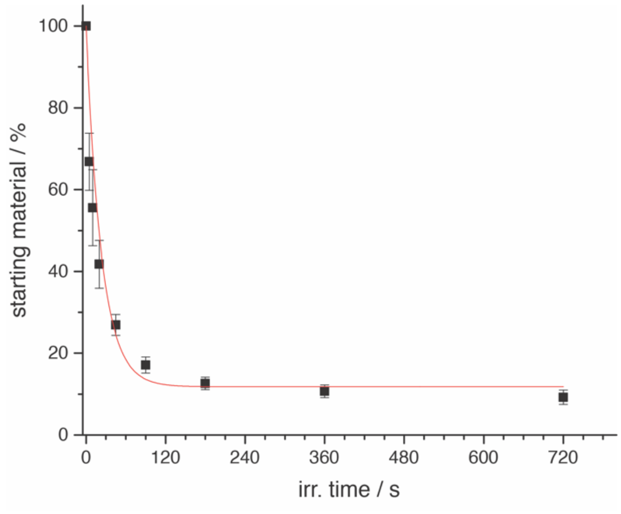

Figure S9: Photolysis curve of compound **9b** in PBS/MeOH (1:1).

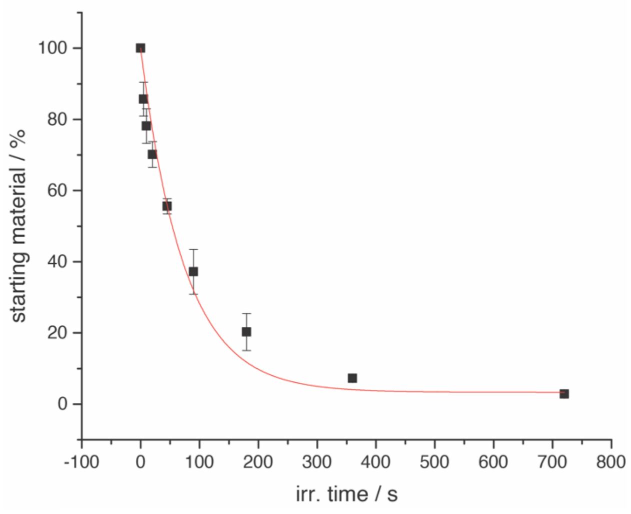

Figure S10: Photolysis curve of compound **11** in PBS/MeOH (1:1).

Measurement of photo- and pH-stability

Compounds **7a** and **7b** were dissolved in MeOH. Different buffers were used at the individual pH values (pH 4 – citrate/phosphate buffer, pH 7 – PBS buffer, pH 10 – CHES buffer). The final samples contained a mixture of MeOH and the respective buffer in a 1:1 ratio. Illumination was done with a LED of 385 nm central wavelength and an output power of roughly 40 mW / cm^2 at the sample position. The sample was constantly stirred to ensure an even irradiation and mixing. Before each measurement the sample was equilibrated for 20 minutes at 20° C. No sample degradation occurred in the equilibration phase. The samples were then illuminated for 60 minutes and spectra taken in 20 sec intervals, resulting in a total of 181 spectra per measurement (including the dark spectrum). Transients of the photodegradation (at the absorption maximum) are shown in Figure S11 c) and d). For simplification, the amplitudes were normalized with respect to the maximum at time zero. This allowed us to easily extract the time $t_{1/2}$ after which half of the sample is degraded. The respective values are shown in the table attached to Figures S11 c) and d).

Two trends are observed: Compound **7b** seems to be more stable during irradiation in comparison to compound **7a**. Highest photostability (or longest $t_{1/2}$) is achieved at pH 7.

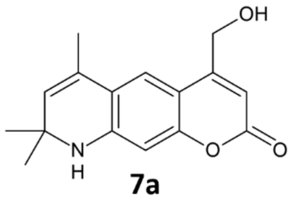

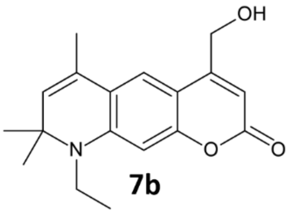

a)

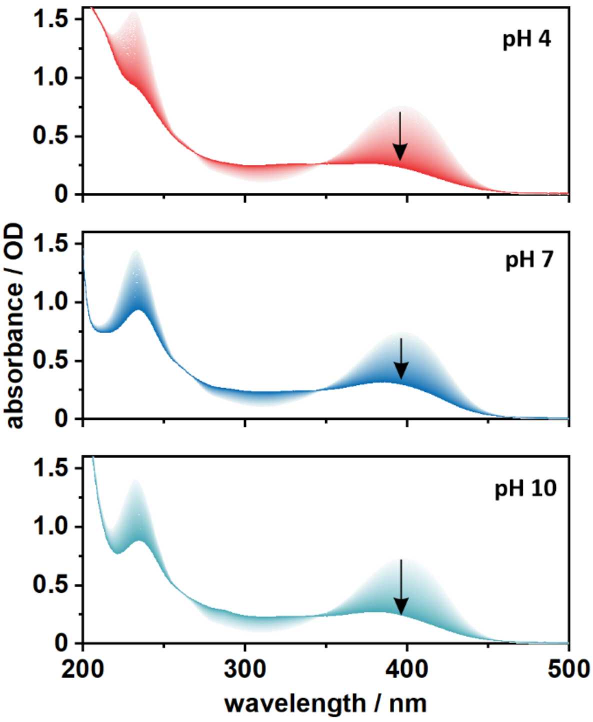

b)

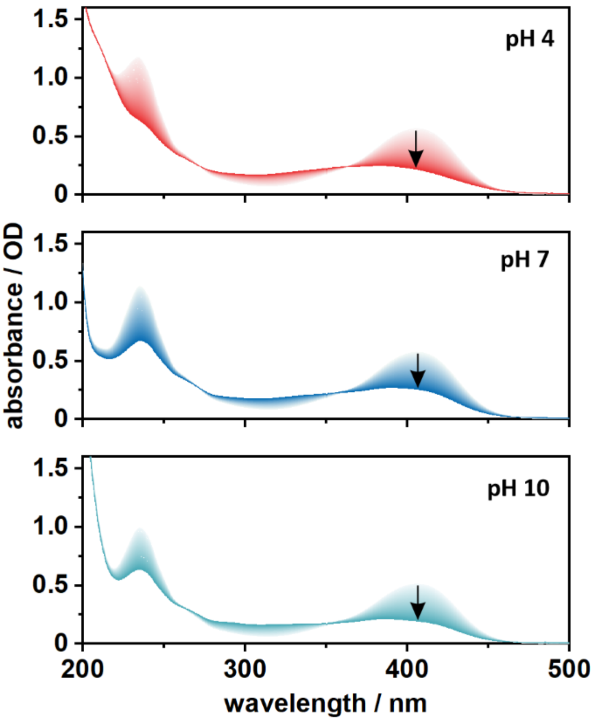

c)

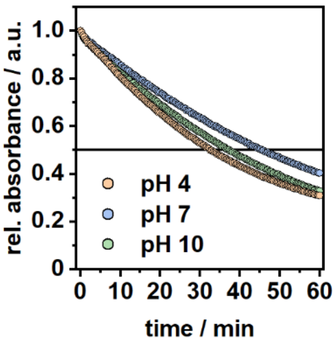

pH	$t_{1/2}$ / s
4	1940
7	2740
10	2240

d)

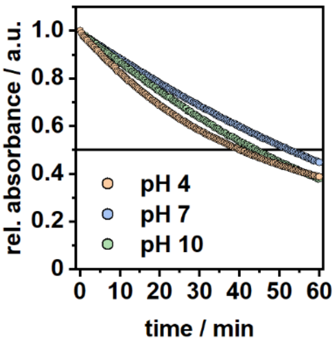

pH	$t_{1/2}$ / s
4	2440
7	3160
10	2660

Figure S11: Photostability and pH-stability measurements of compounds **7a** and **7b**.

NMR spectra

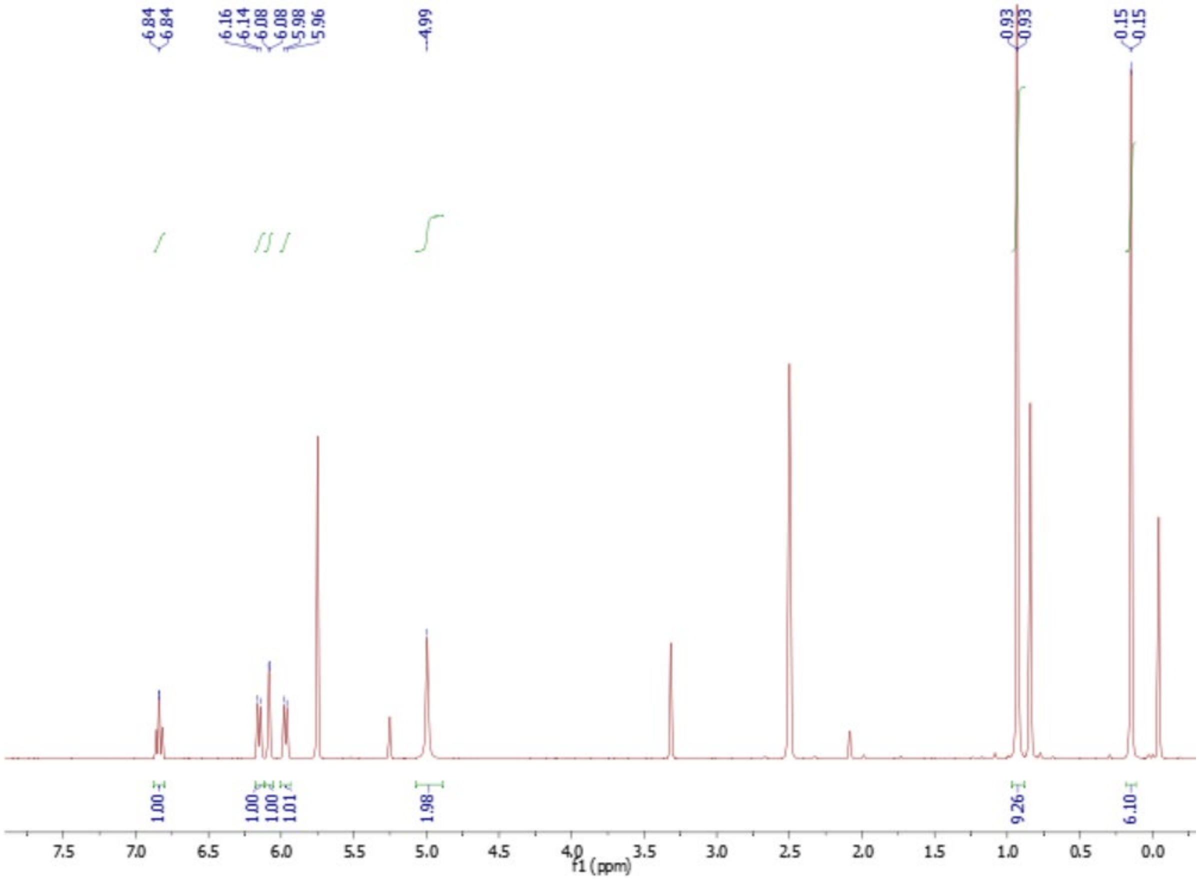

^{1}H-NMR spectrum of compound **3** in DMSO-d$_6$.

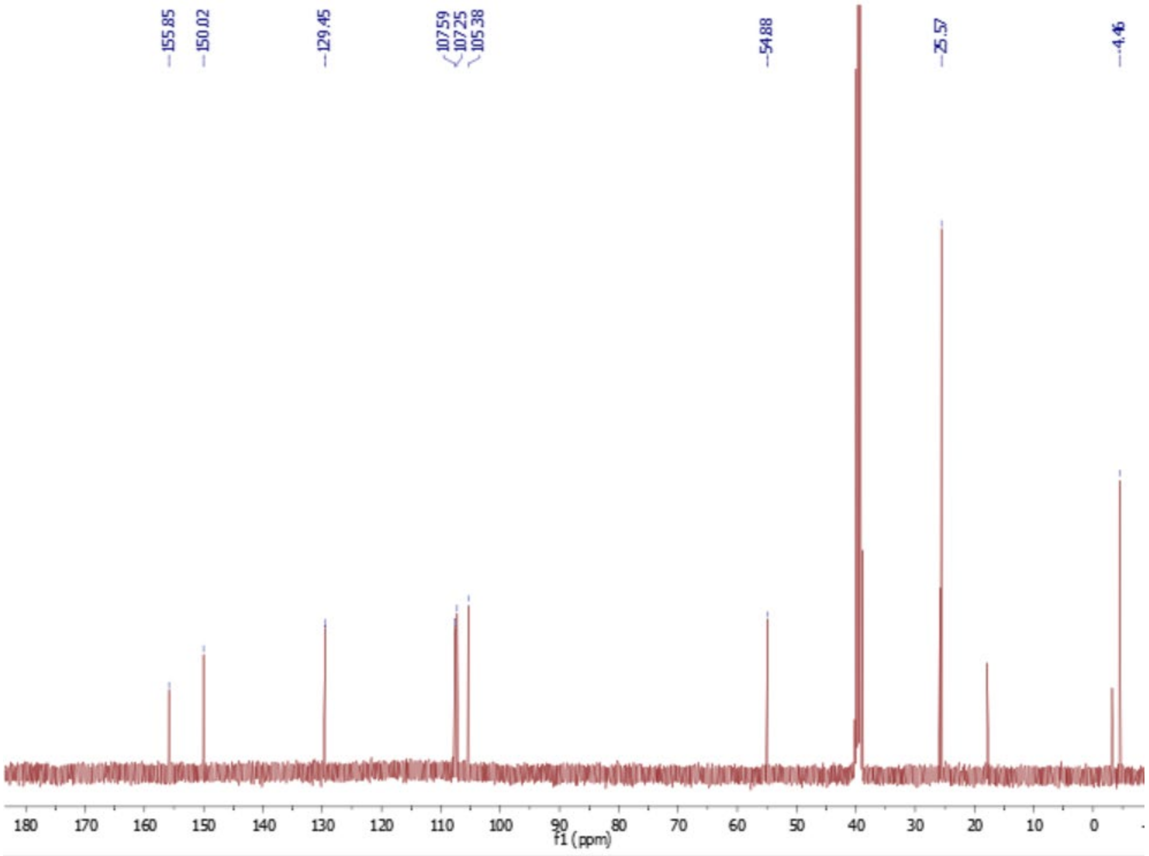

^{13}C-NMR spectrum of compound **3** in DMSO-d$_6$.

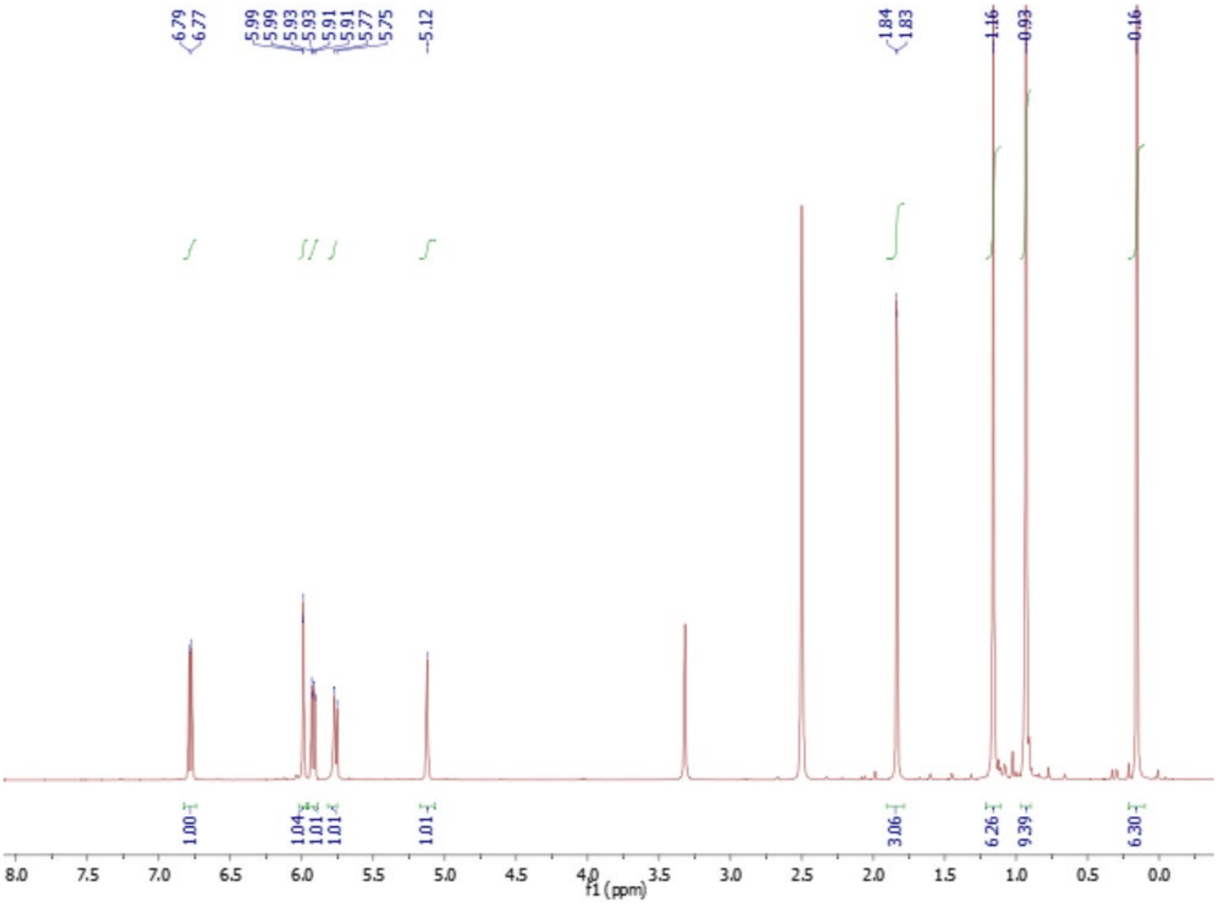

^{1}H-NMR spectrum of compound **4a** in DMSO-d_6.

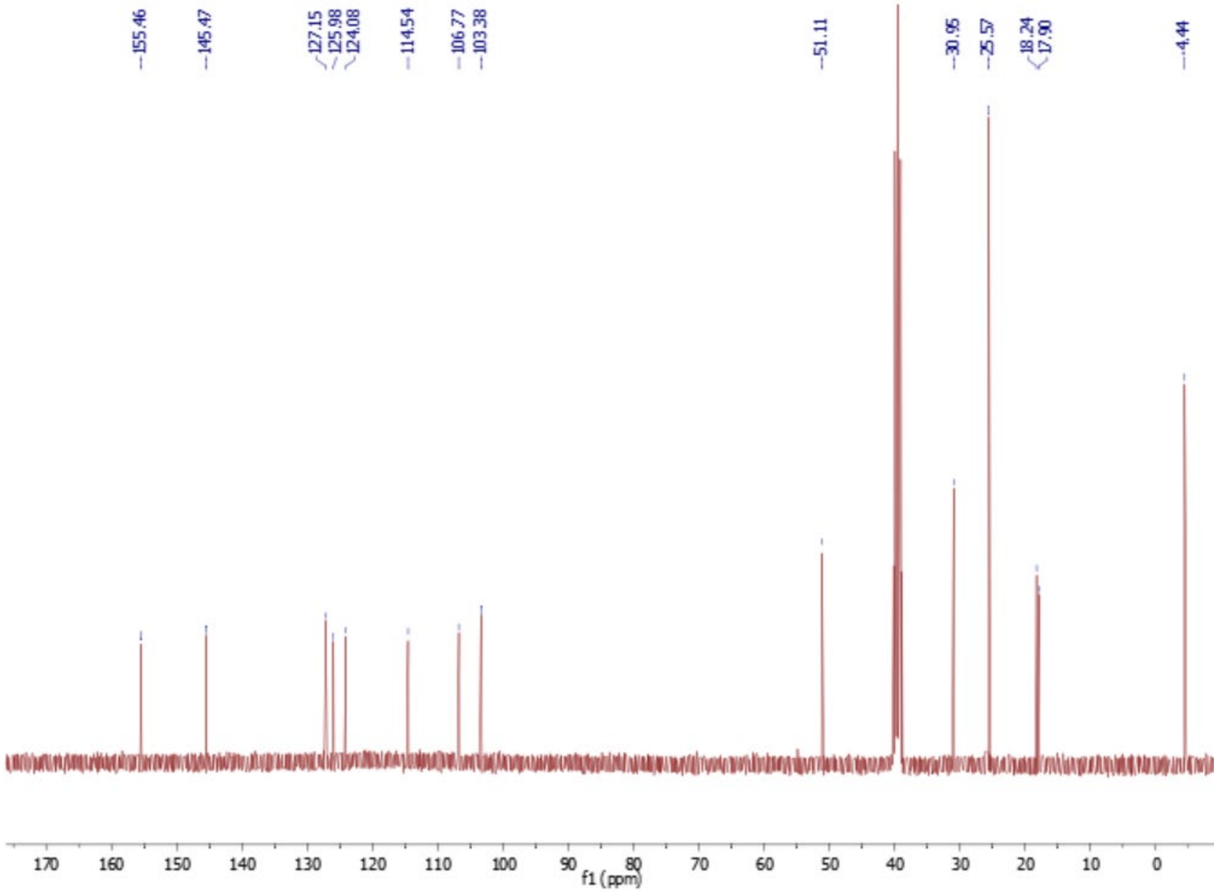

^{13}C-NMR spectrum of compound **4a** in DMSO-d_6.

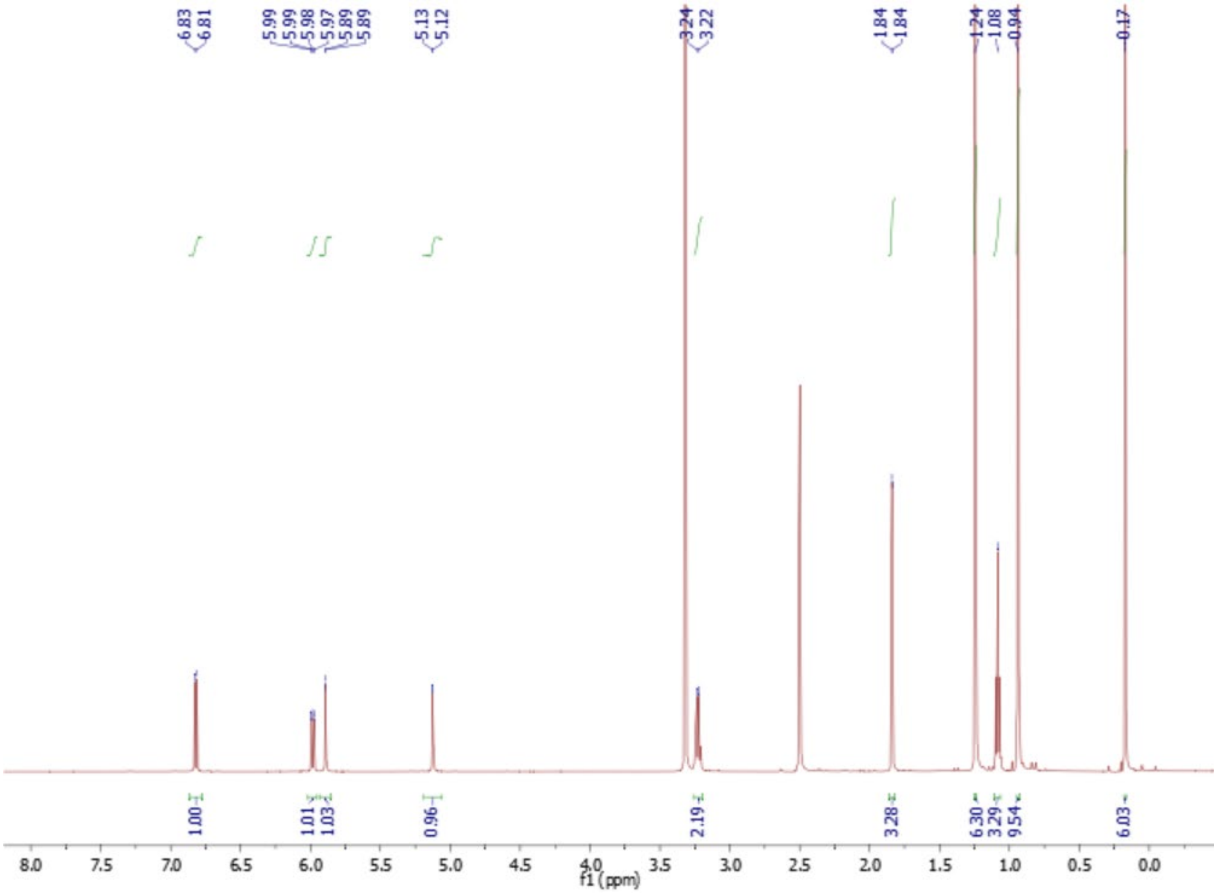

^{1}H-NMR spectrum of compound **4b** in DMSO-d_6.

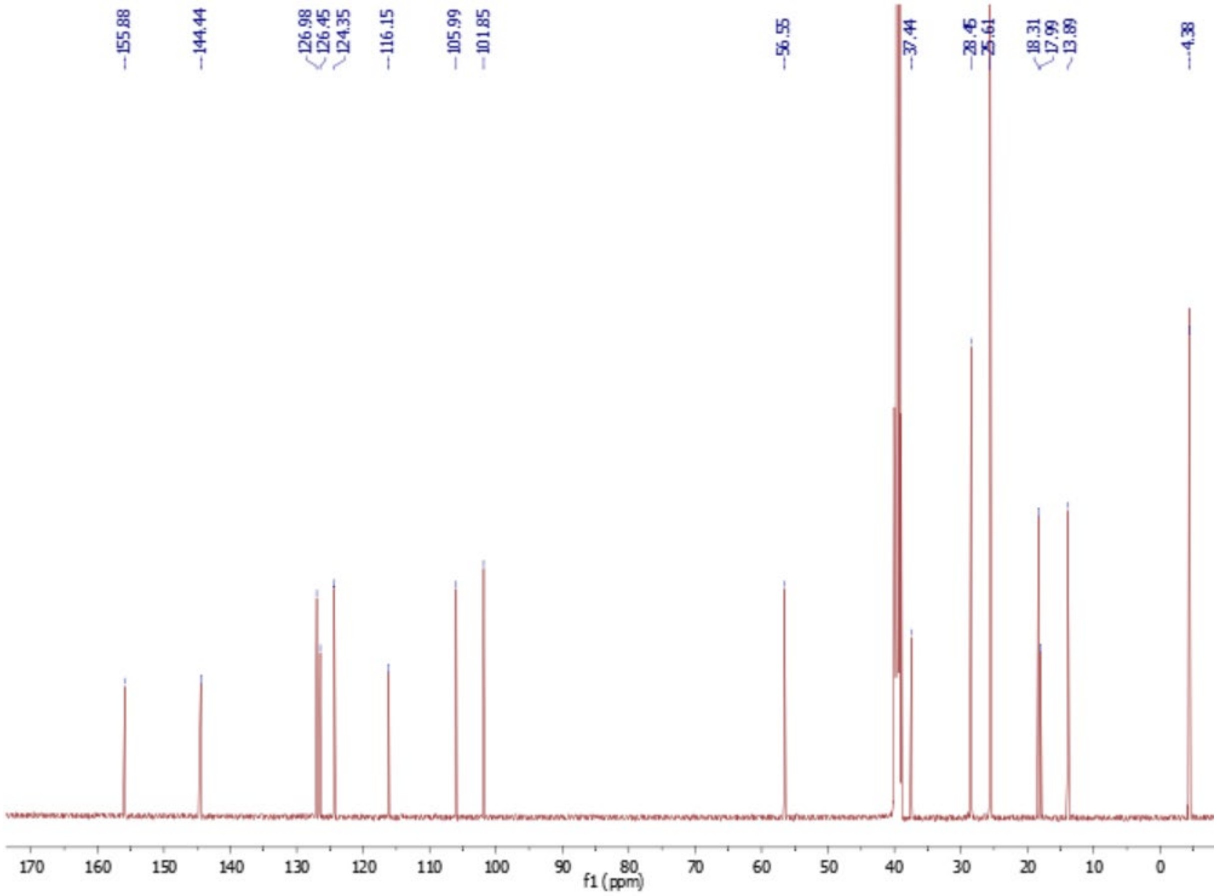

^{13}C-NMR spectrum of compound **4b** in DMSO-d_6.

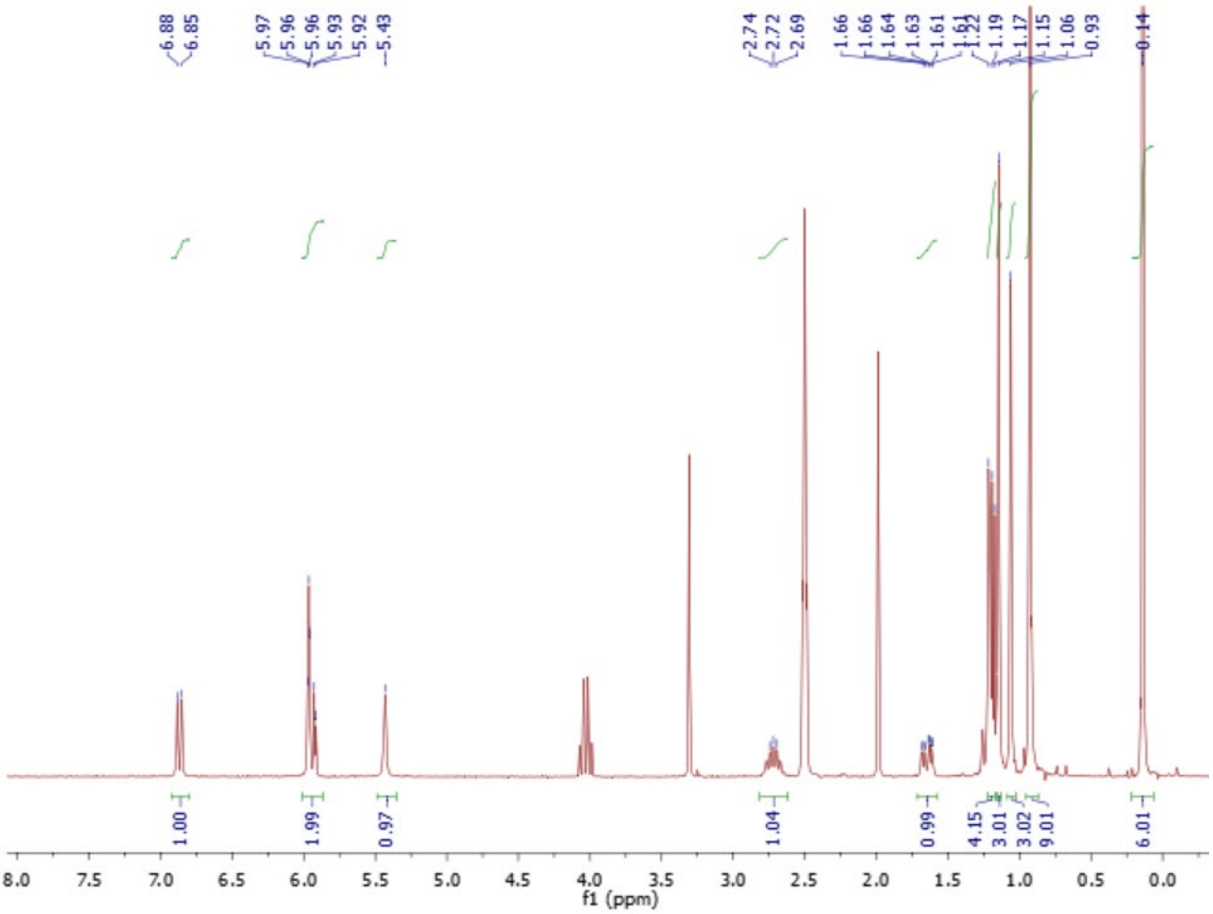

^{1}H-NMR spectrum of compound **4c** in DMSO-d_6.

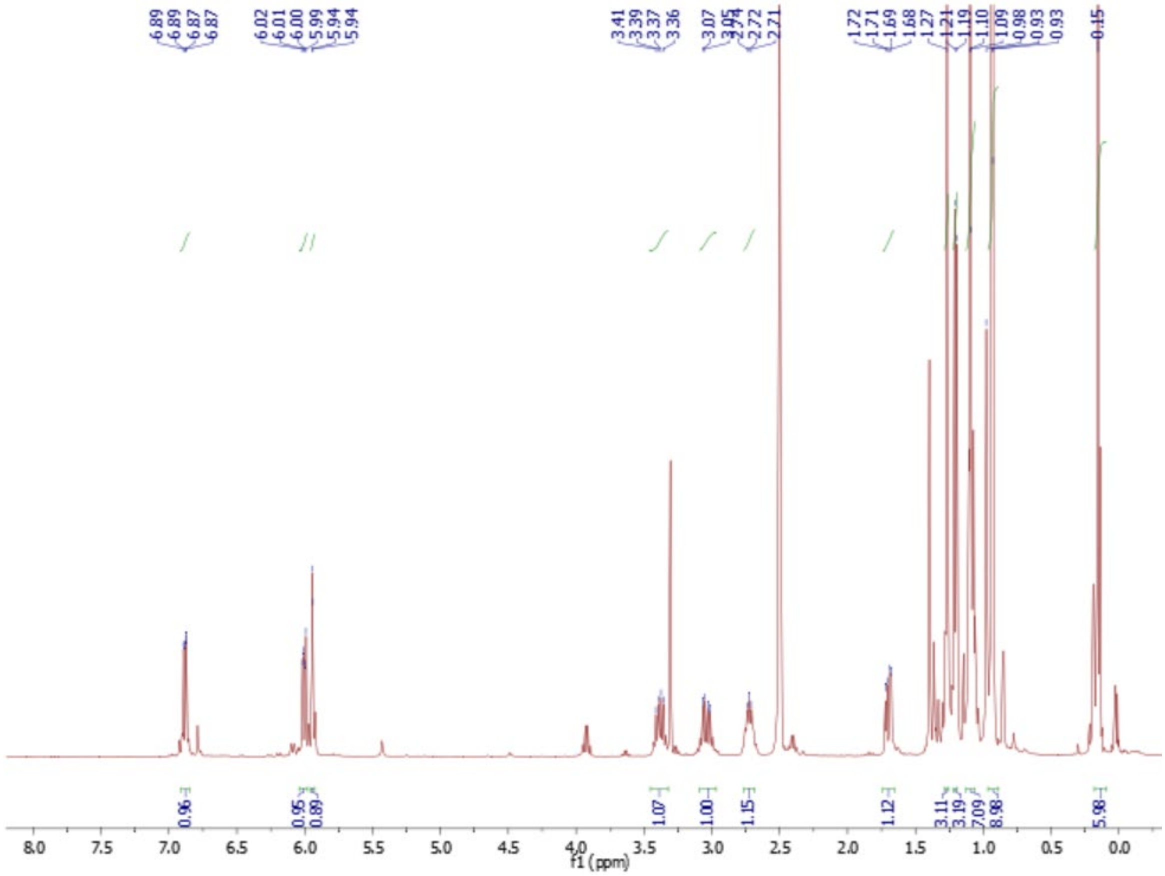

^{1}H-NMR spectrum of compound **4d** in DMSO-d_6.

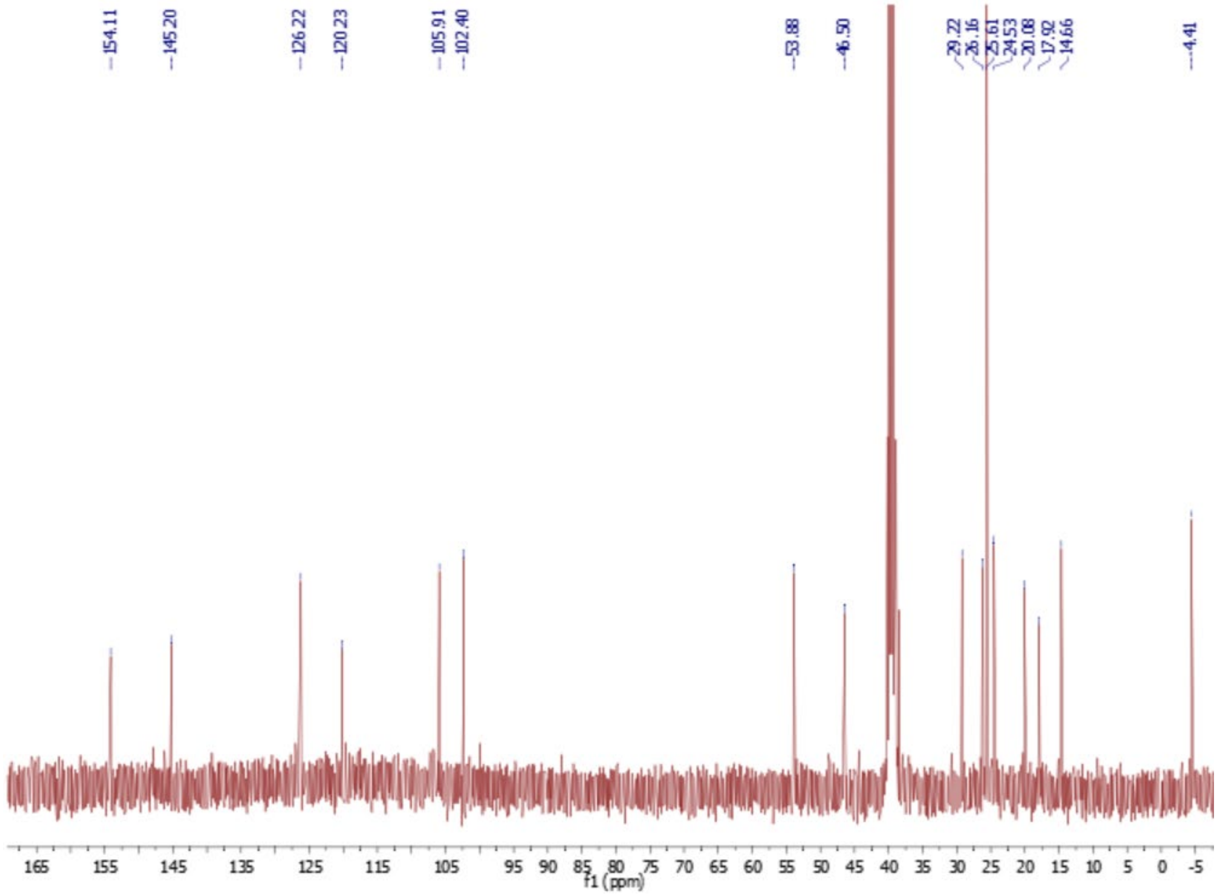

^{13}C-NMR spectrum of compound **4d** in DMSO-d$_6$.

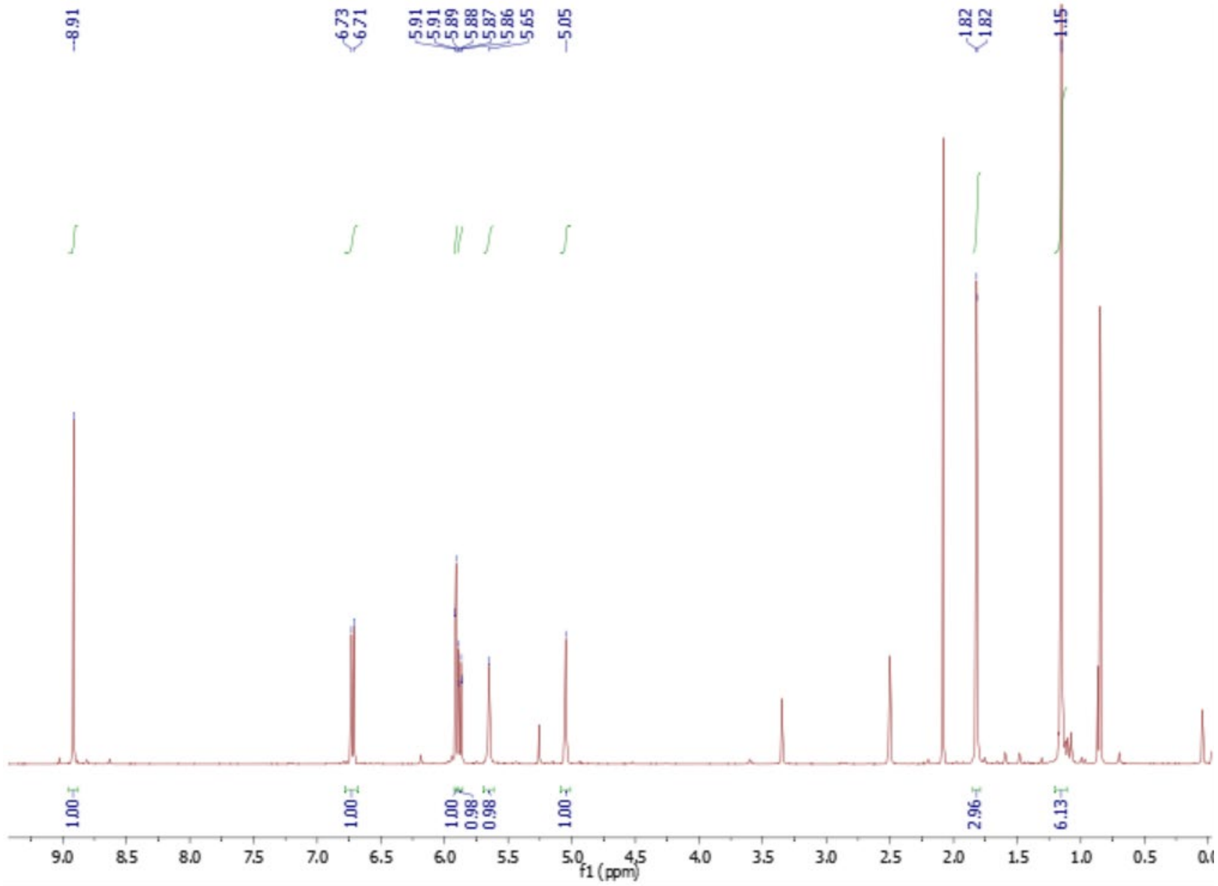

^{1}H-NMR spectrum of compound **5a** in DMSO-d$_6$.

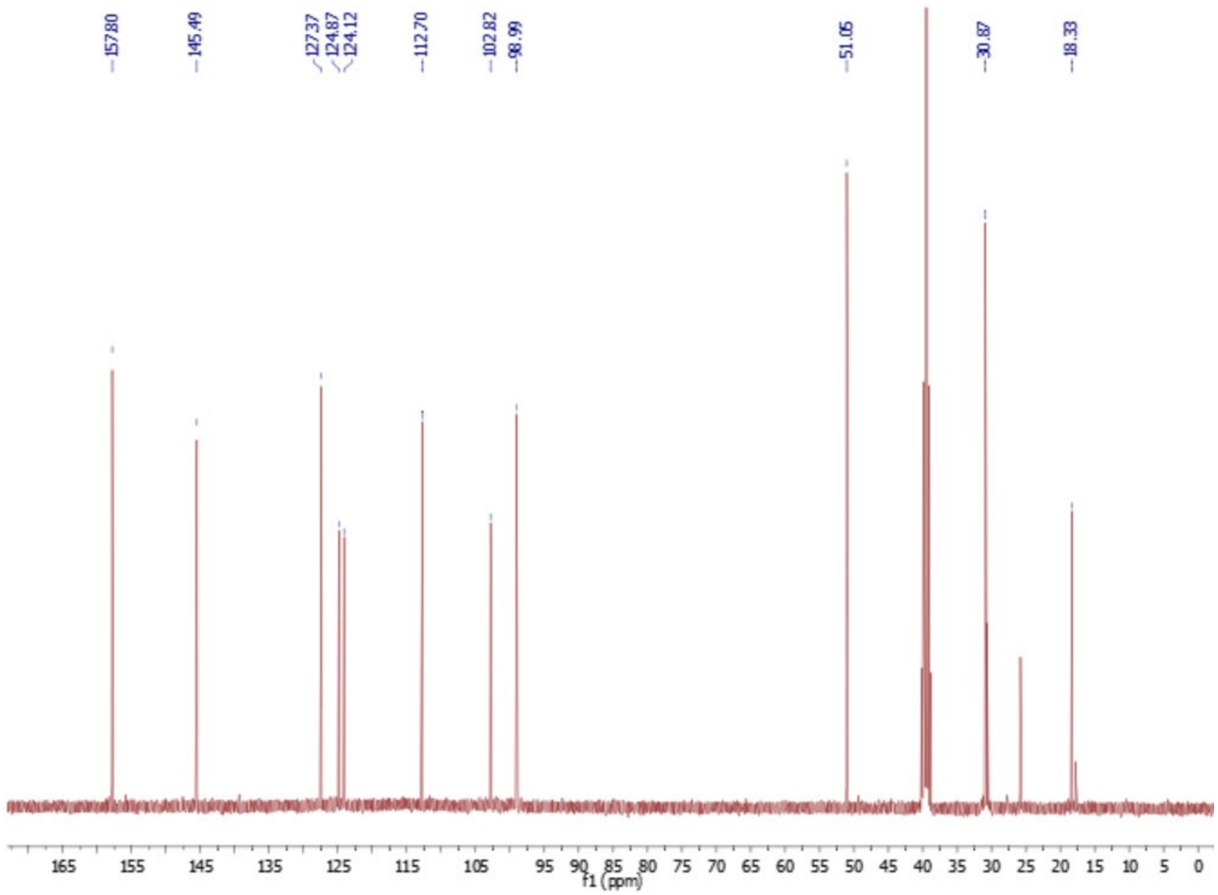

^{13}C-NMR of compound **5a** in DMSO-d_6.

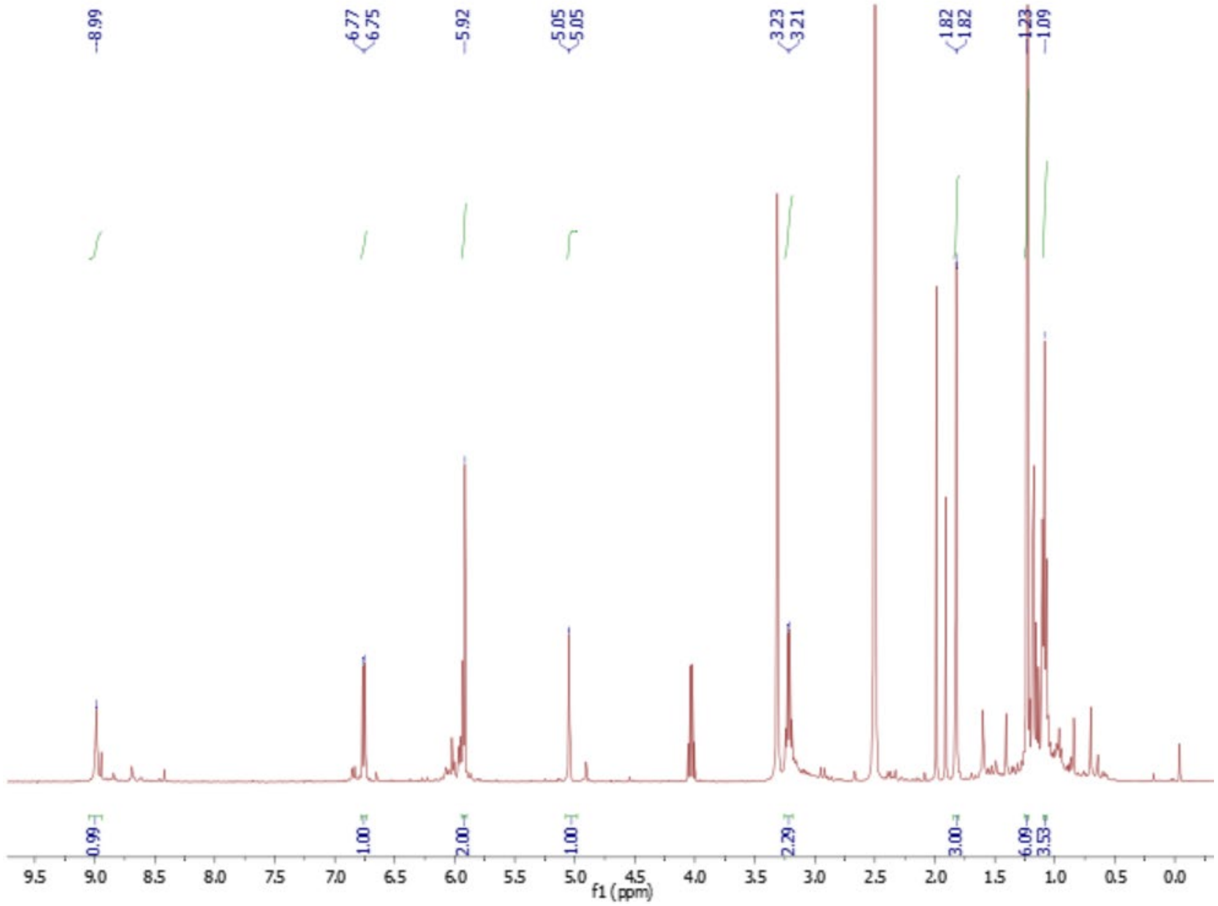

^{1}H-NMR spectrum of compound **5b** in DMSO-d_6.

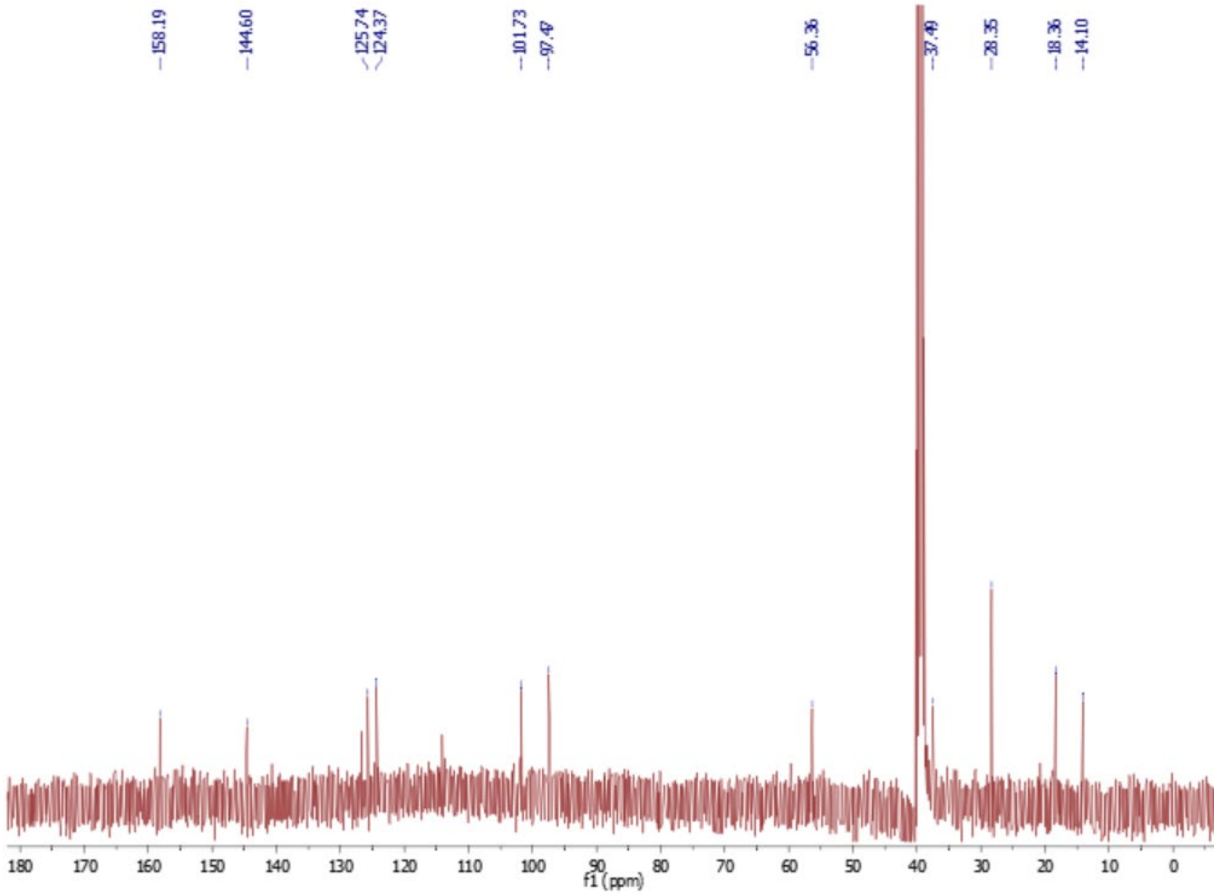

^{13}C-NMR spectrum of compound **5b** in DMSO-d_6.

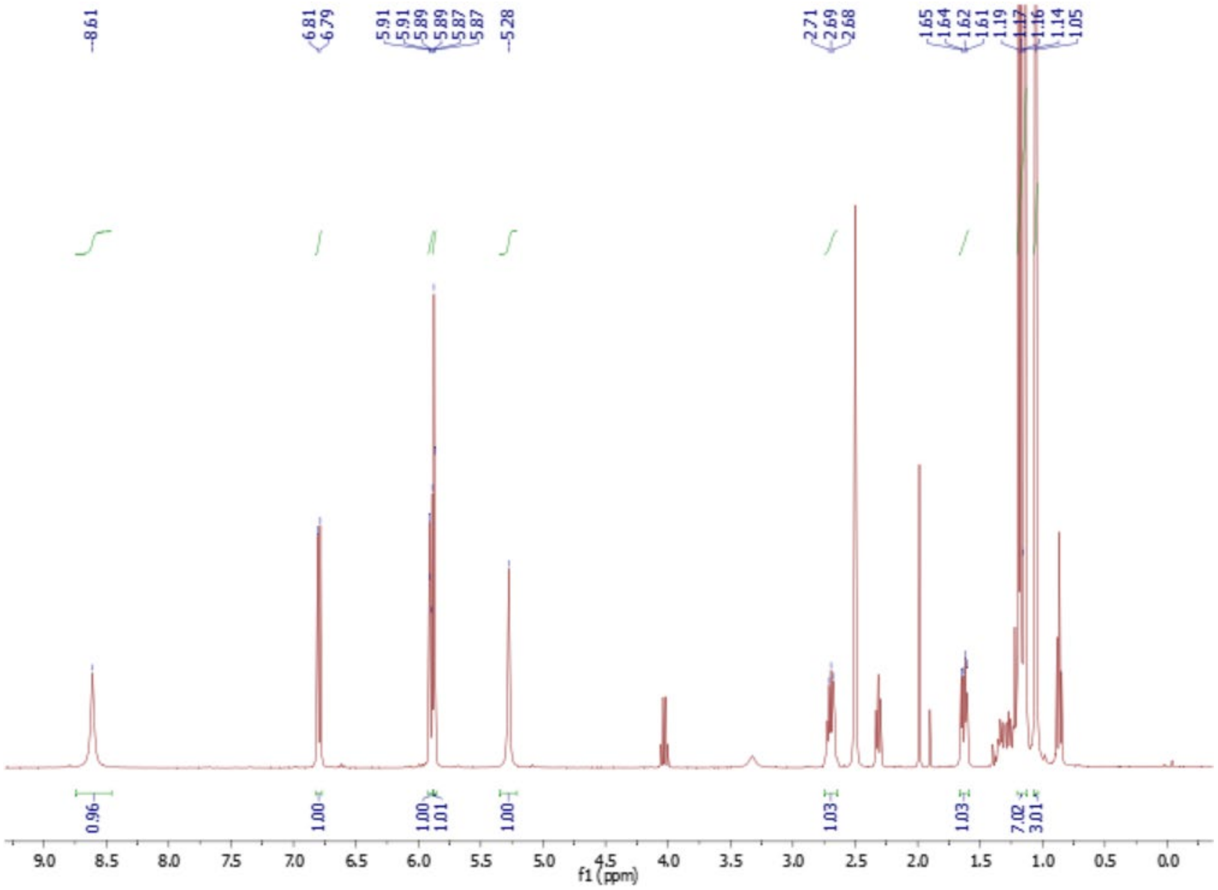

^{1}H-NMR spectrum of compound **5c** in DMSO-d_6.

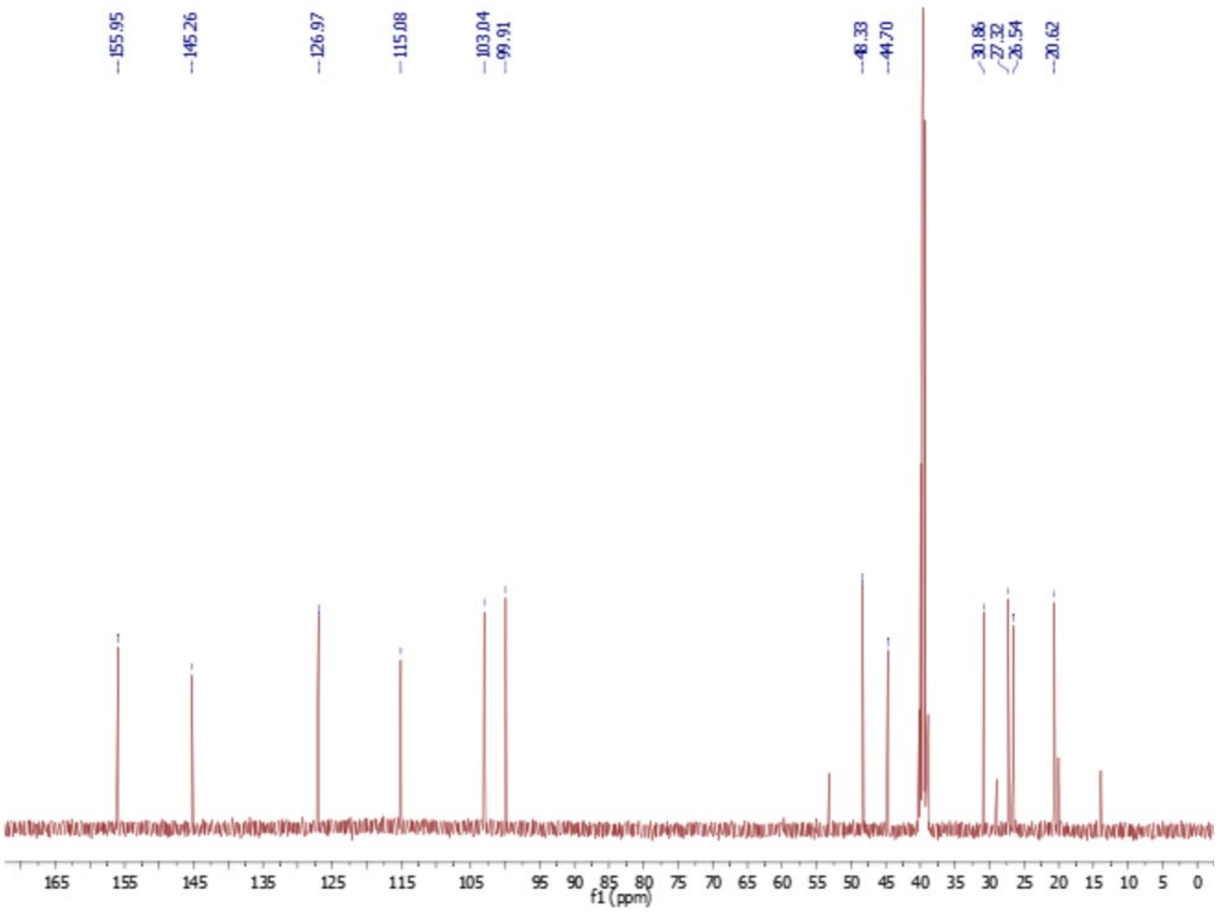

^{13}C-NMR spectrum of compound **5c** in DMSO-d_6.

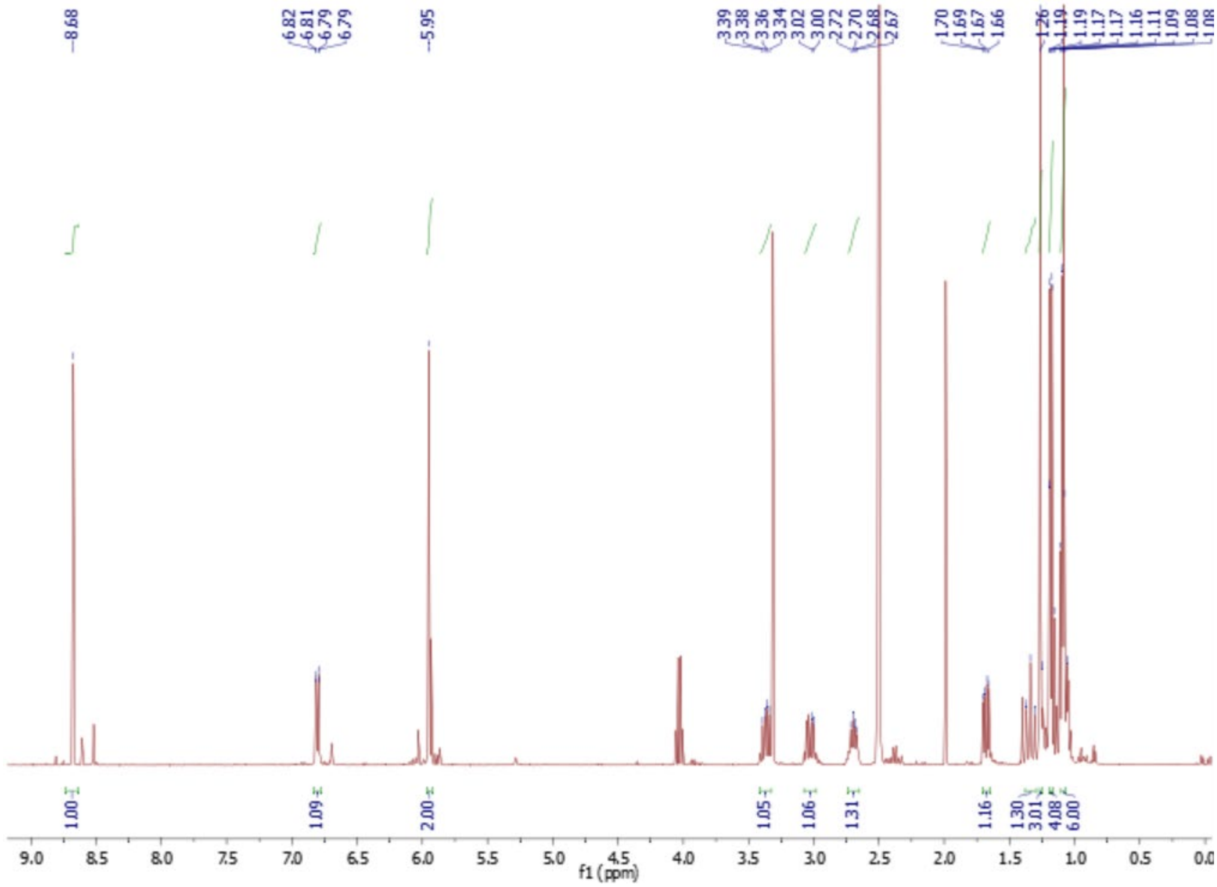

^{1}H-NMR spectrum of compound **5d** in DMSO-d_6.

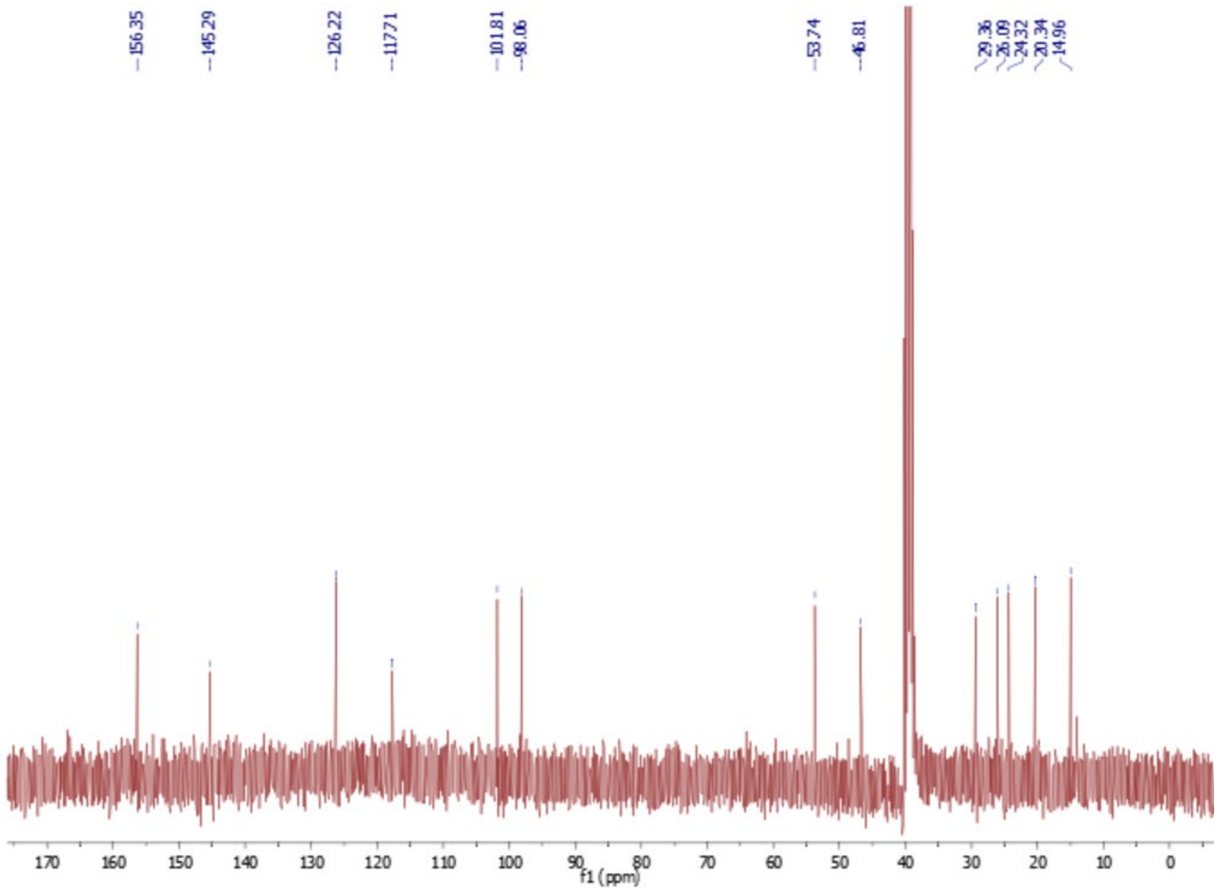

^{13}C-NMR spectrum of compound **5d** in DMSO-d$_6$.

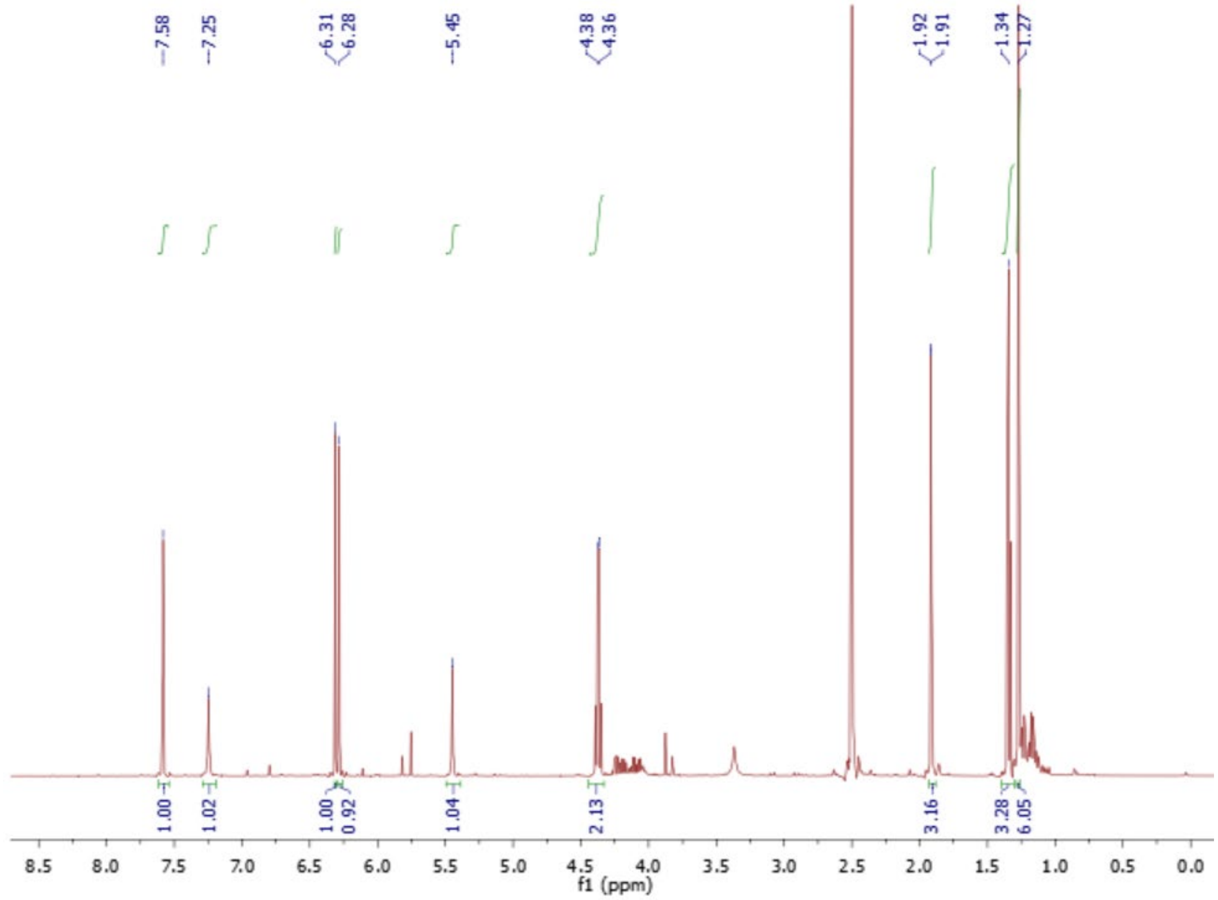

^{1}H-NMR spectrum of compound **6a** in DMSO-d$_6$.

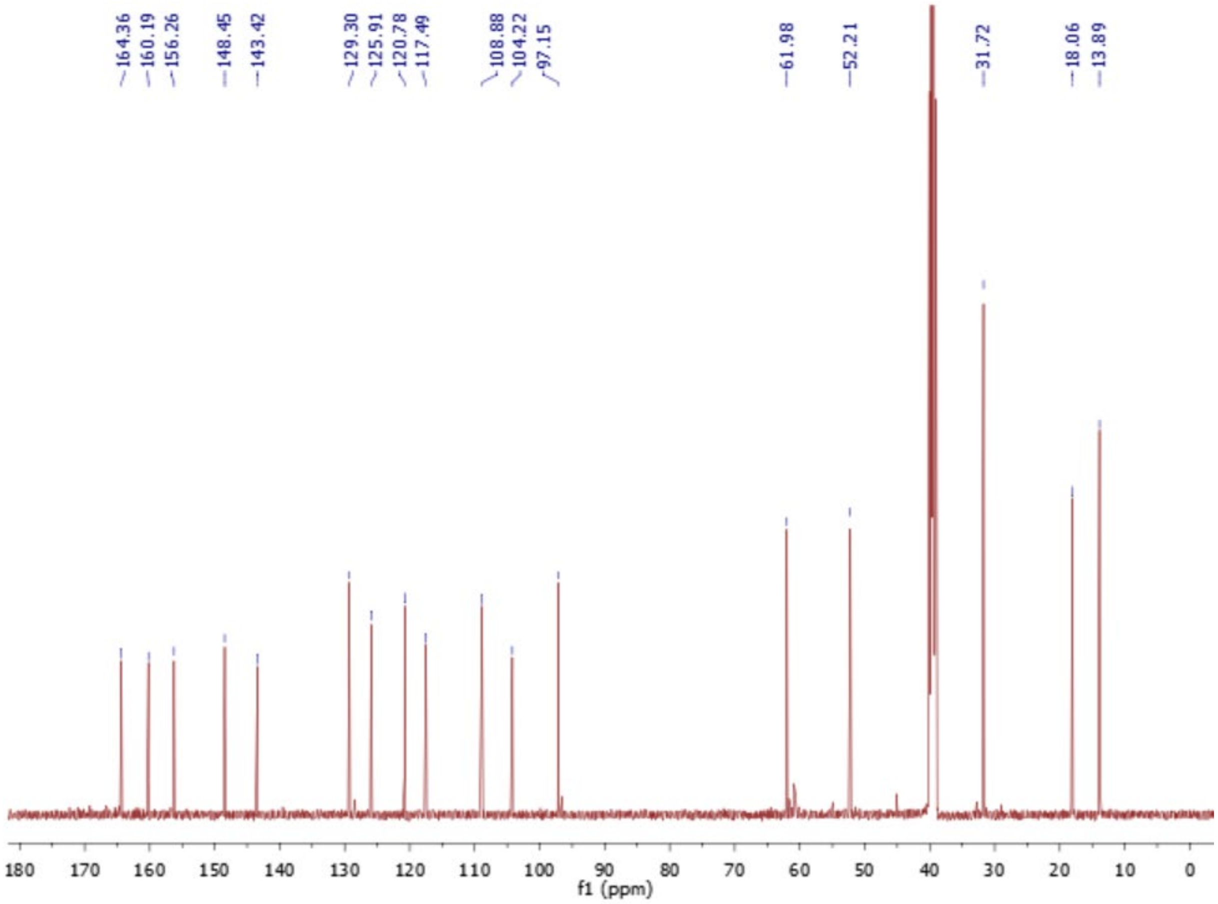

^{13}C-NMR spectrum of compound **6a** in DMSO-d_6.

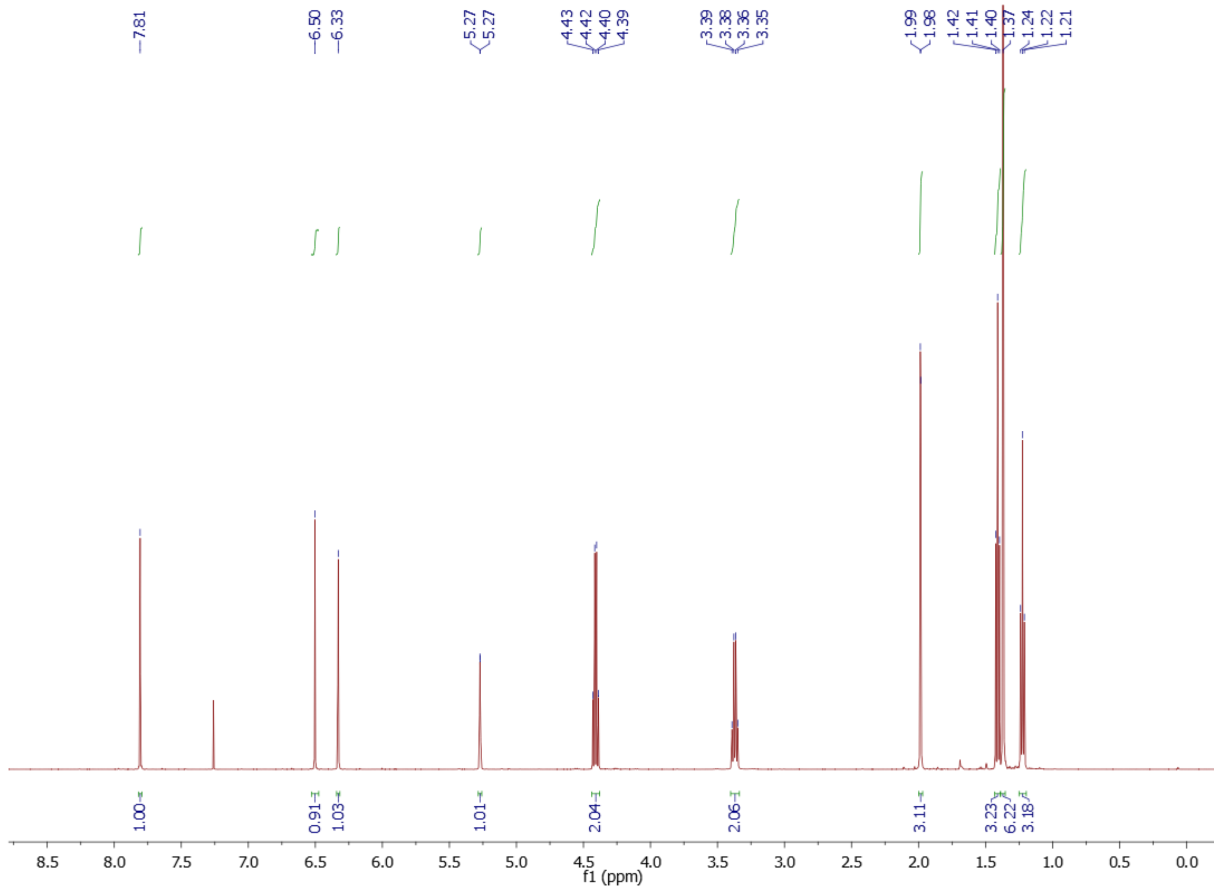

^{1}H-NMR spectrum of compound **6b** in $CDCl_3$.

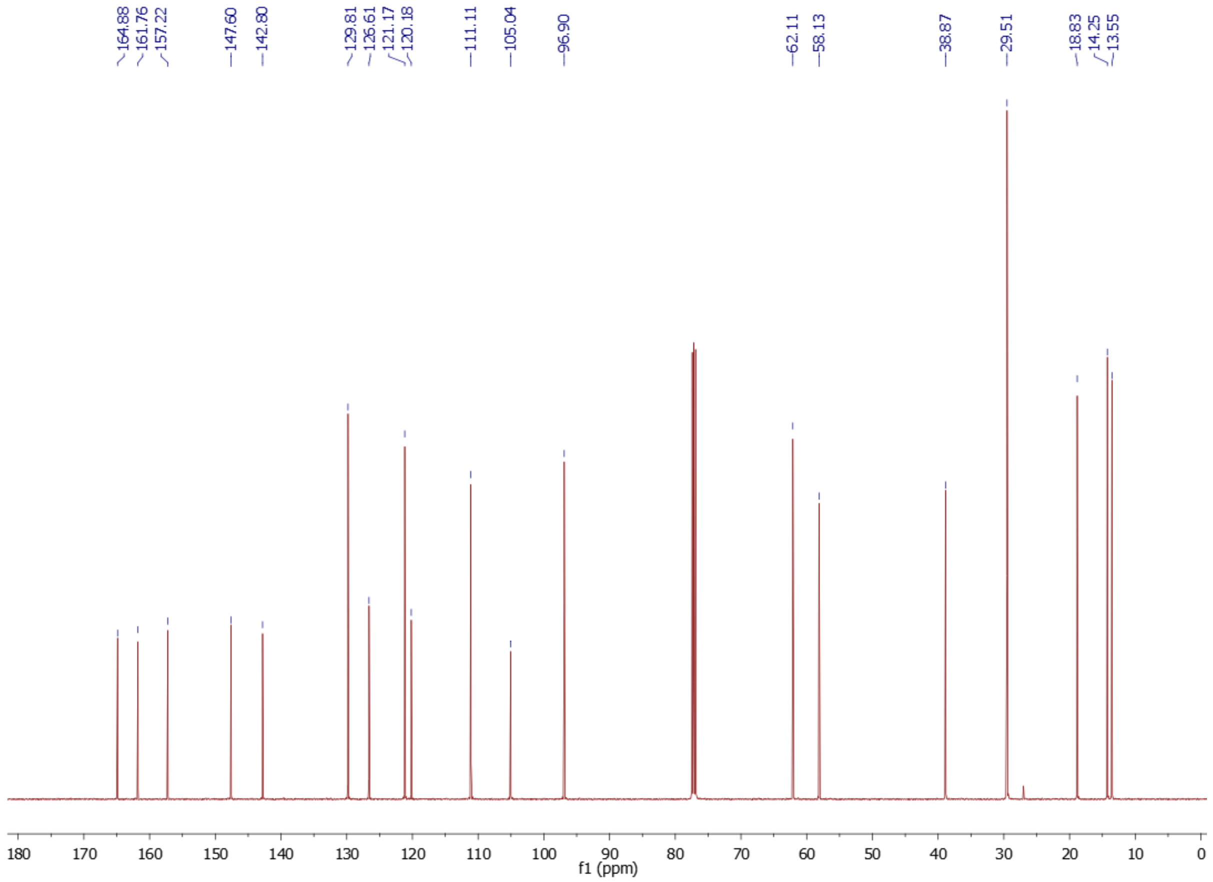

^{13}C-NMR spectrum of compound **6b** in $CDCl_3$.

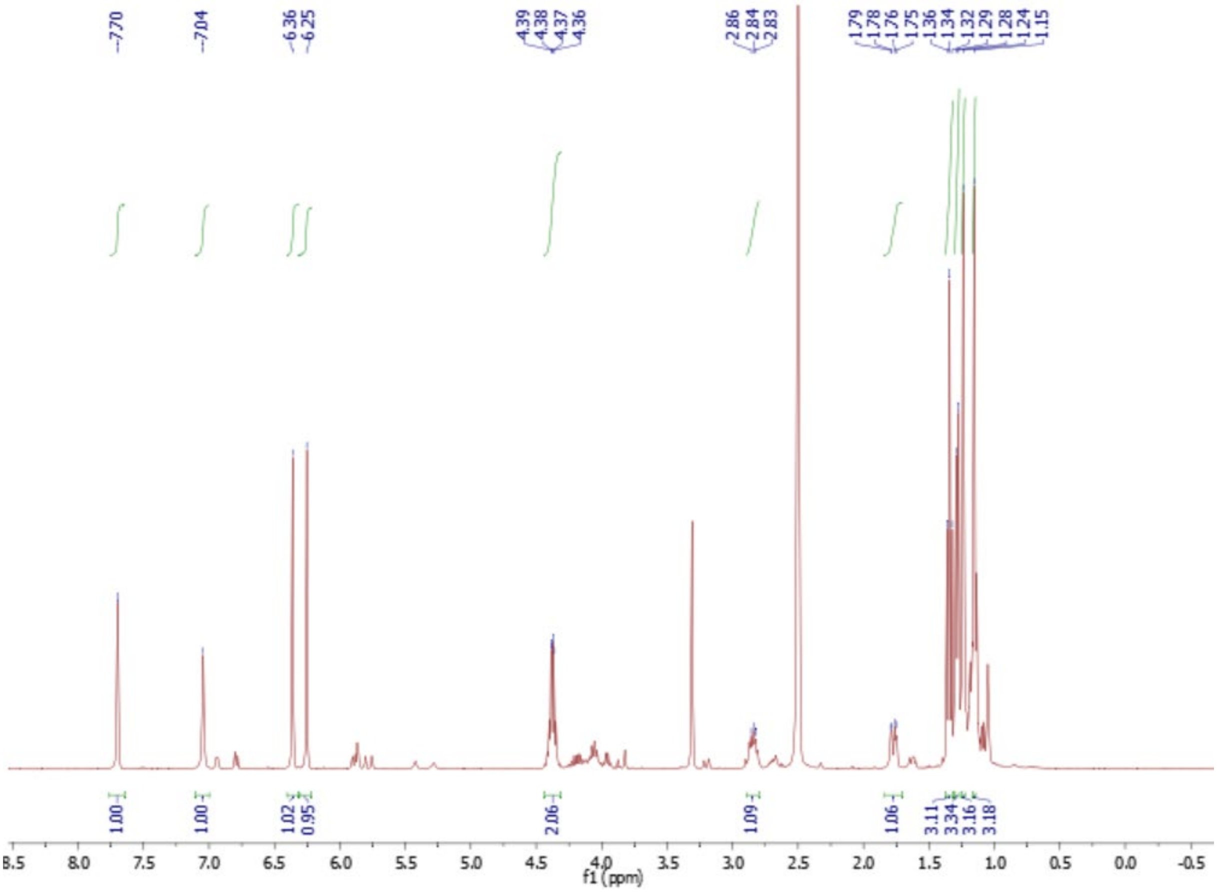

^{1}H-NMR spectrum of compound **6c** in DMSO-d_6.

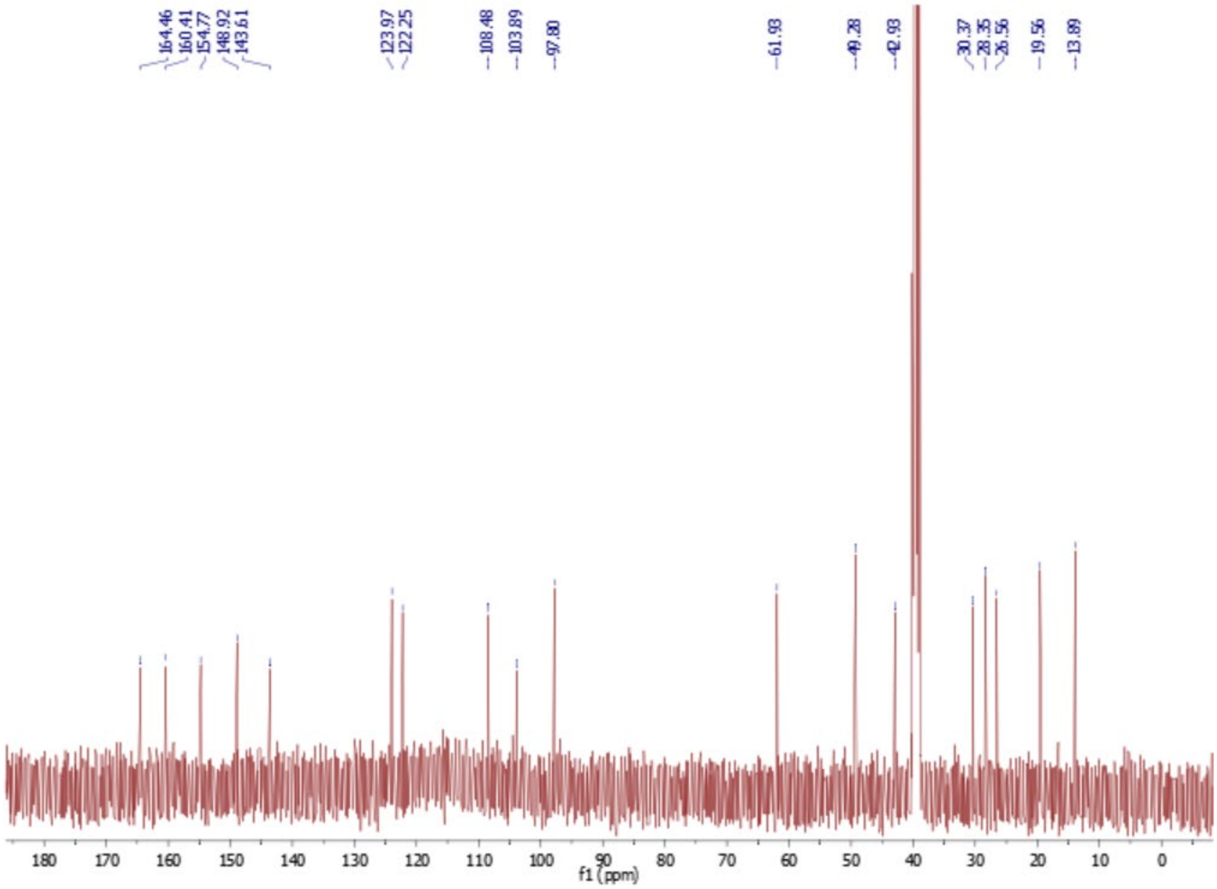

^{13}C-NMR spectrum of compound **6c** in DMSO-d_6.

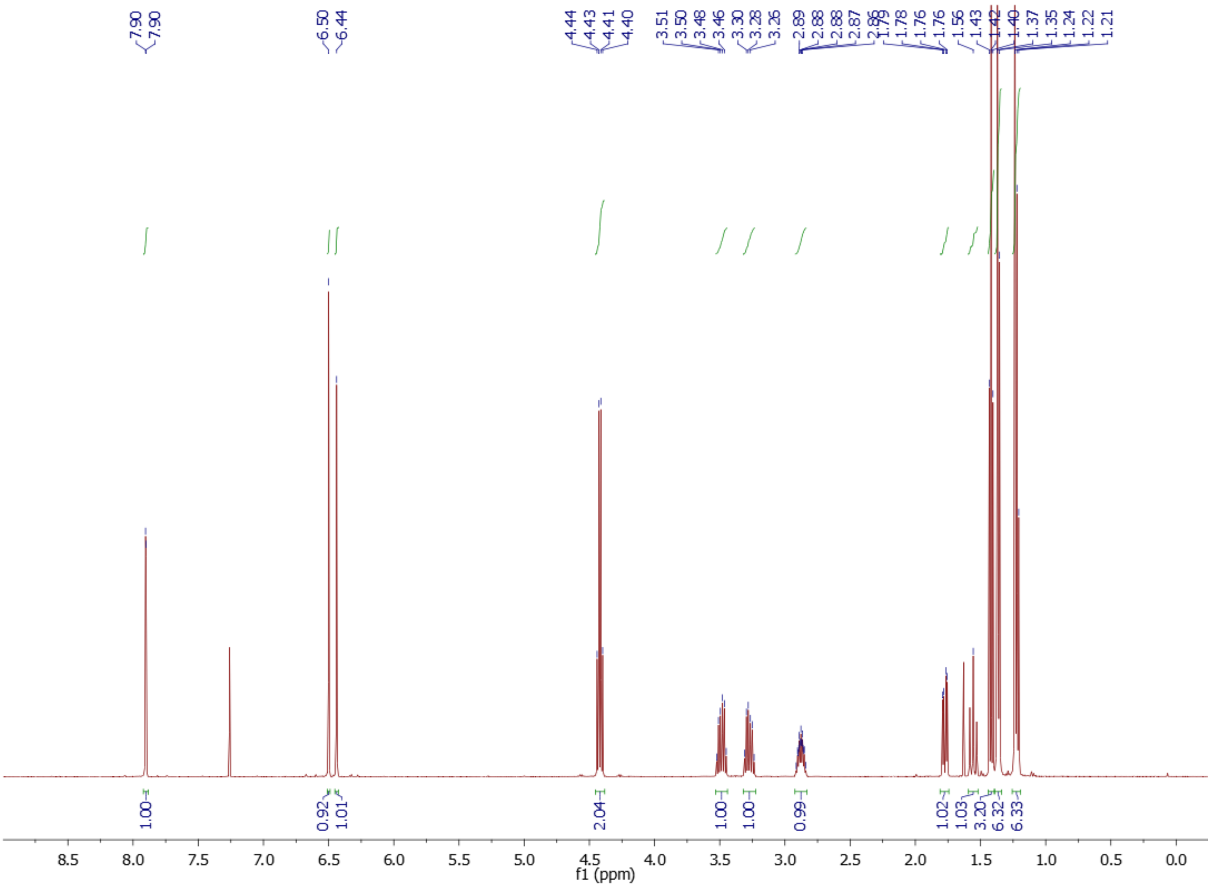

^{1}H-NMR spectrum of compound **6d** in $CDCl_3$.

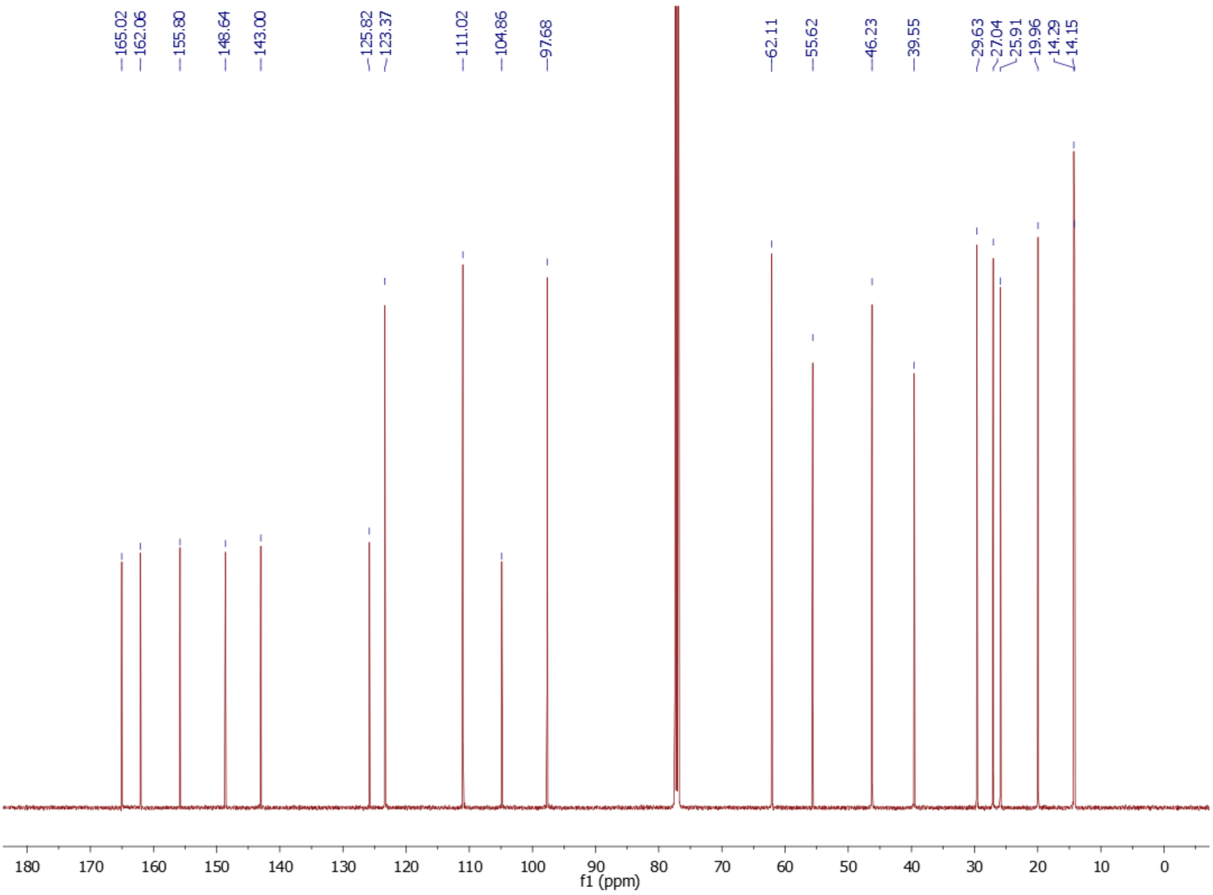

^{13}C-NMR spectrum of compound **6d** in $CDCl_3$.

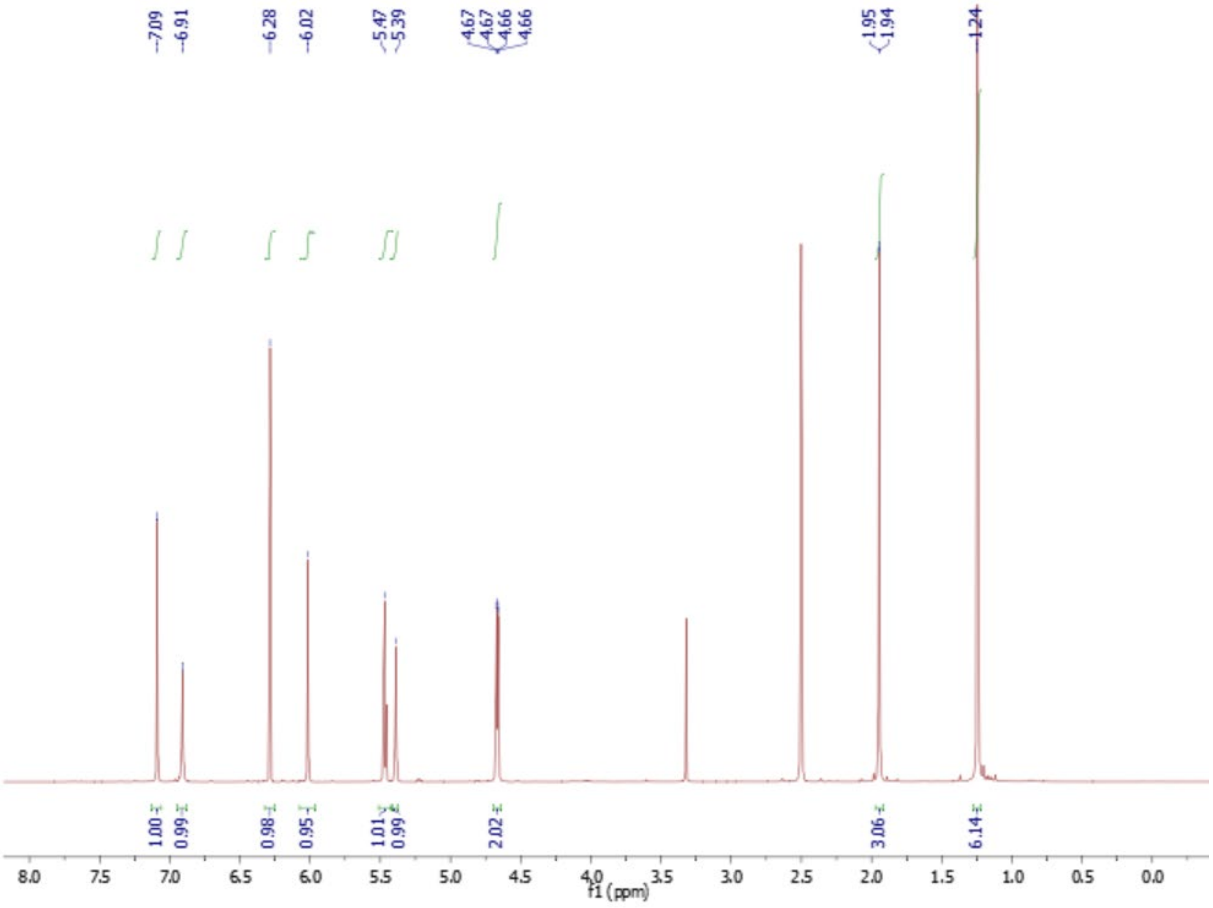

^{1}H-NMR spectrum of compound **7a** in DMSO-d_6.

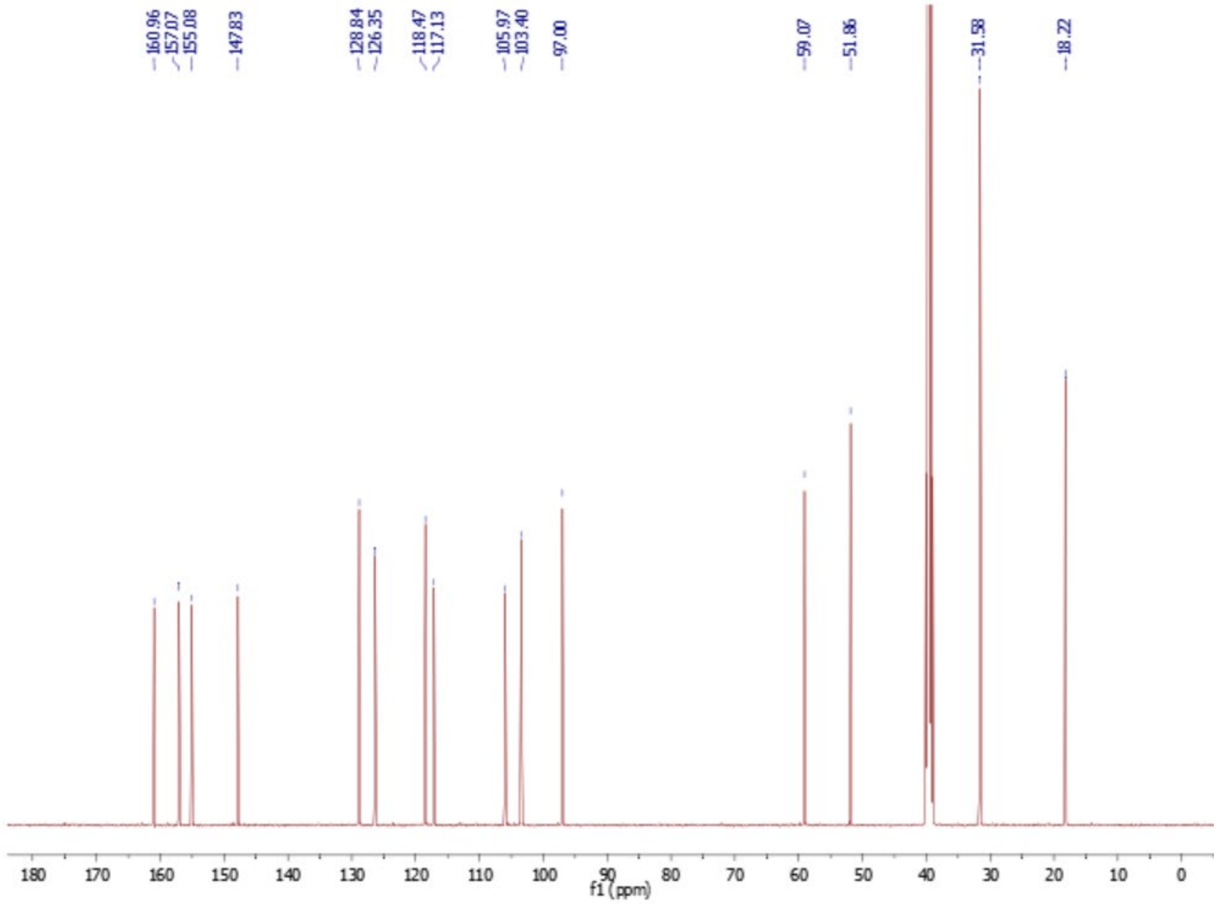

^{13}C-NMR spectrum of compound **7a** in DMSO-d$_6$.

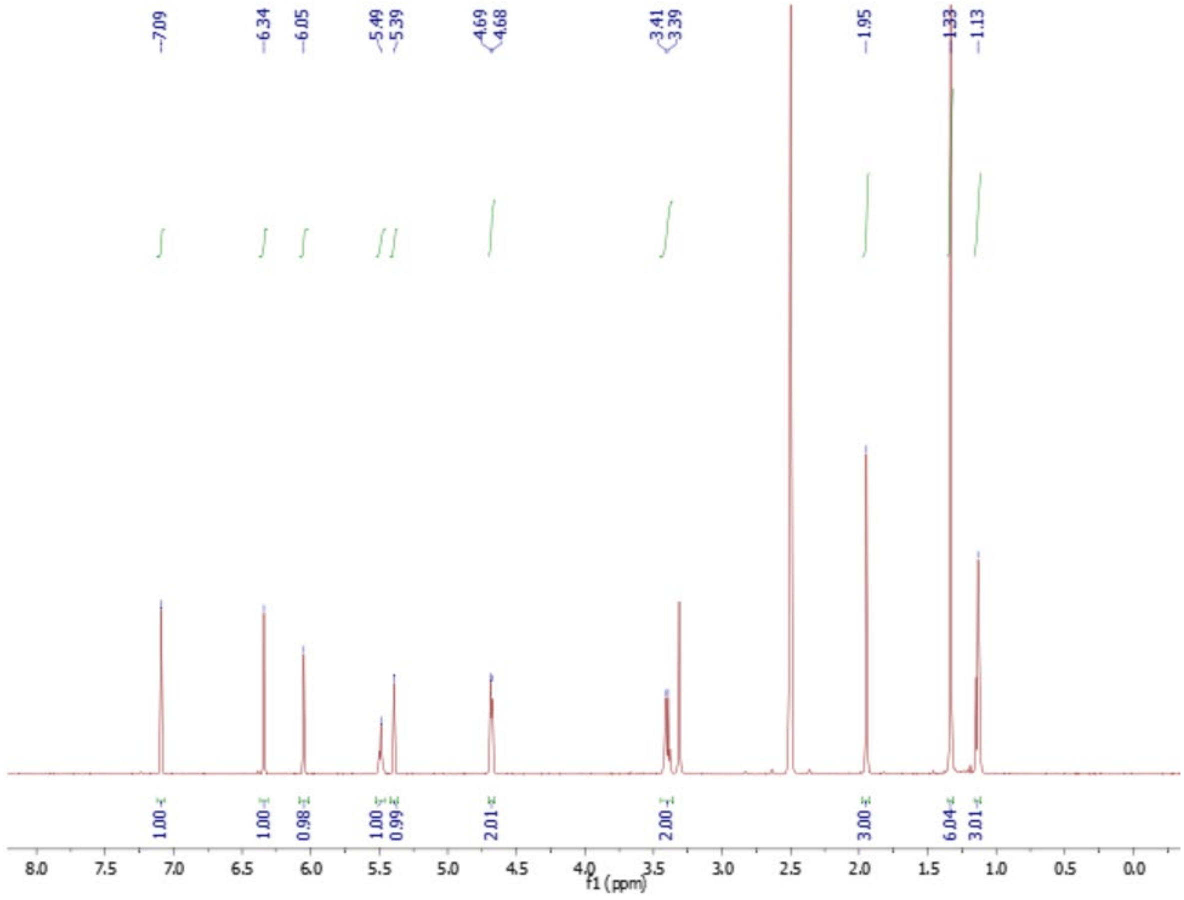

^{1}H-NMR spectrum of compound **7b** in DMSO-d$_6$.

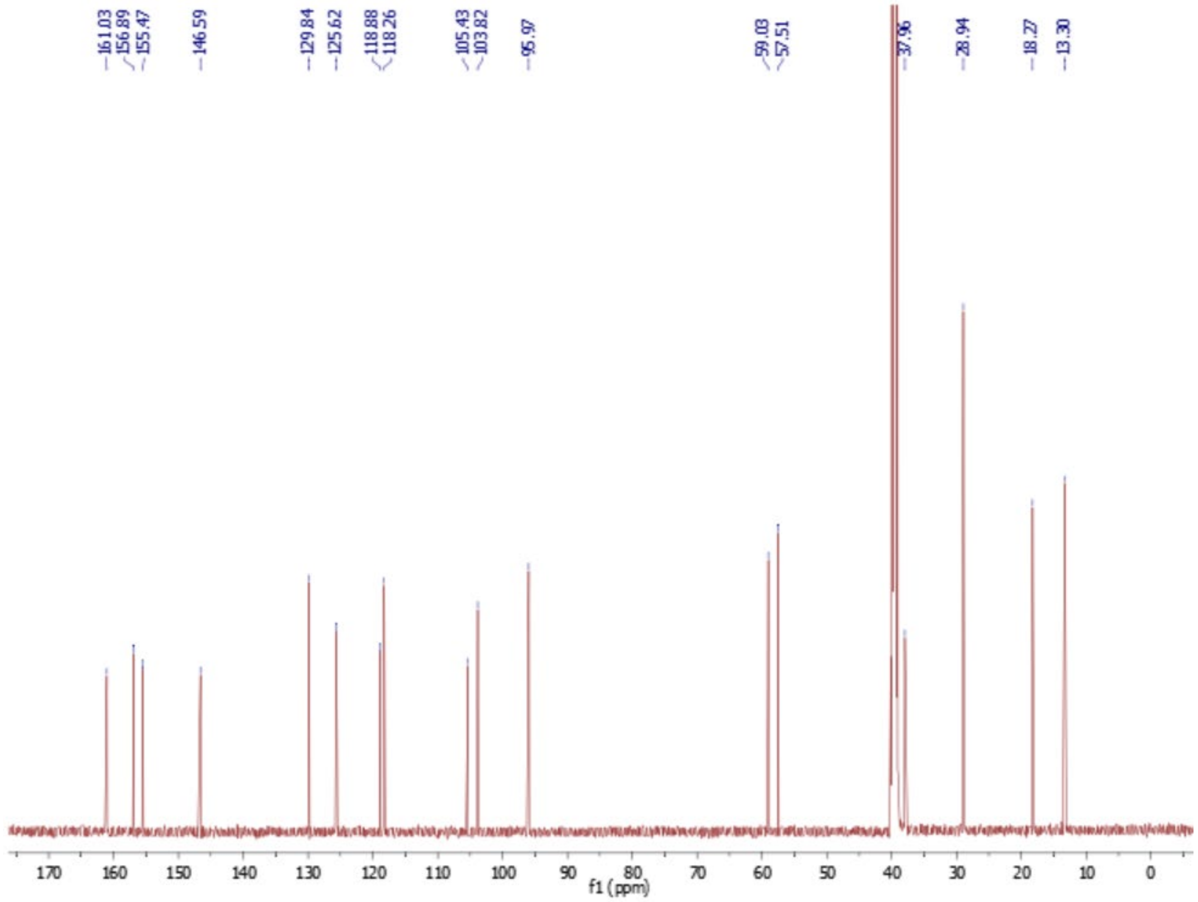

^{13}C-NMR spectrum of compound **7b** in DMSO-d_6.

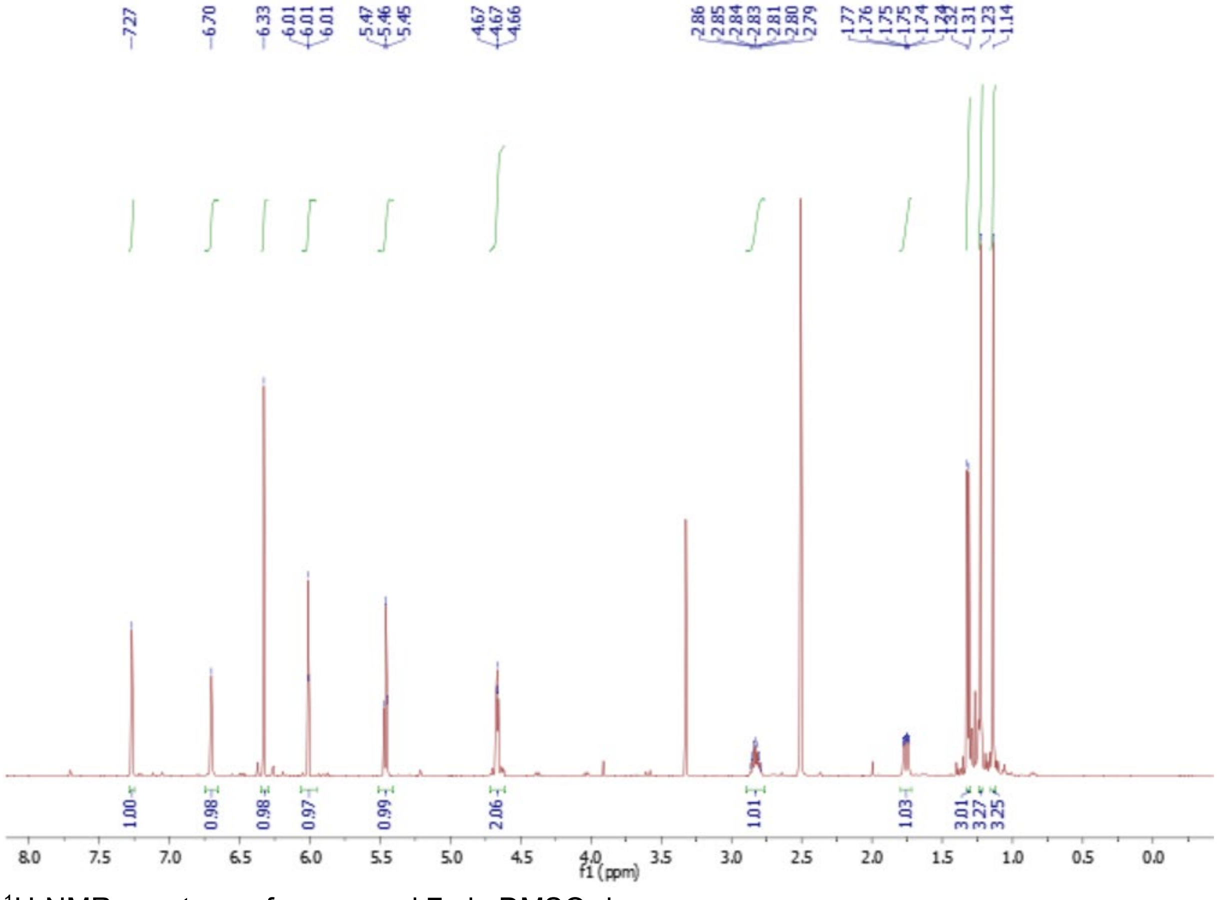

^{1}H-NMR spectrum of compound **7c** in DMSO-d_6.

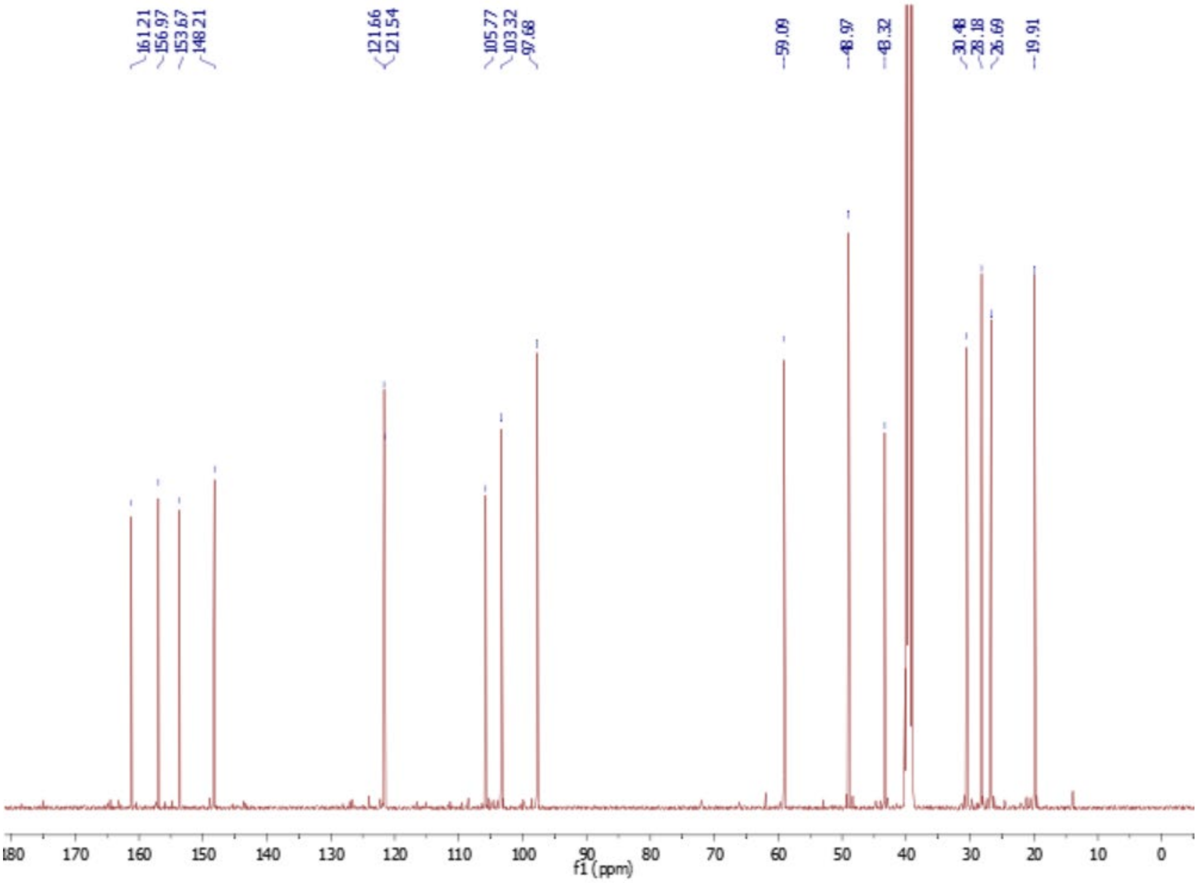

^{13}C-NMR spectrum of compound **7c** in DMSO-d$_6$.

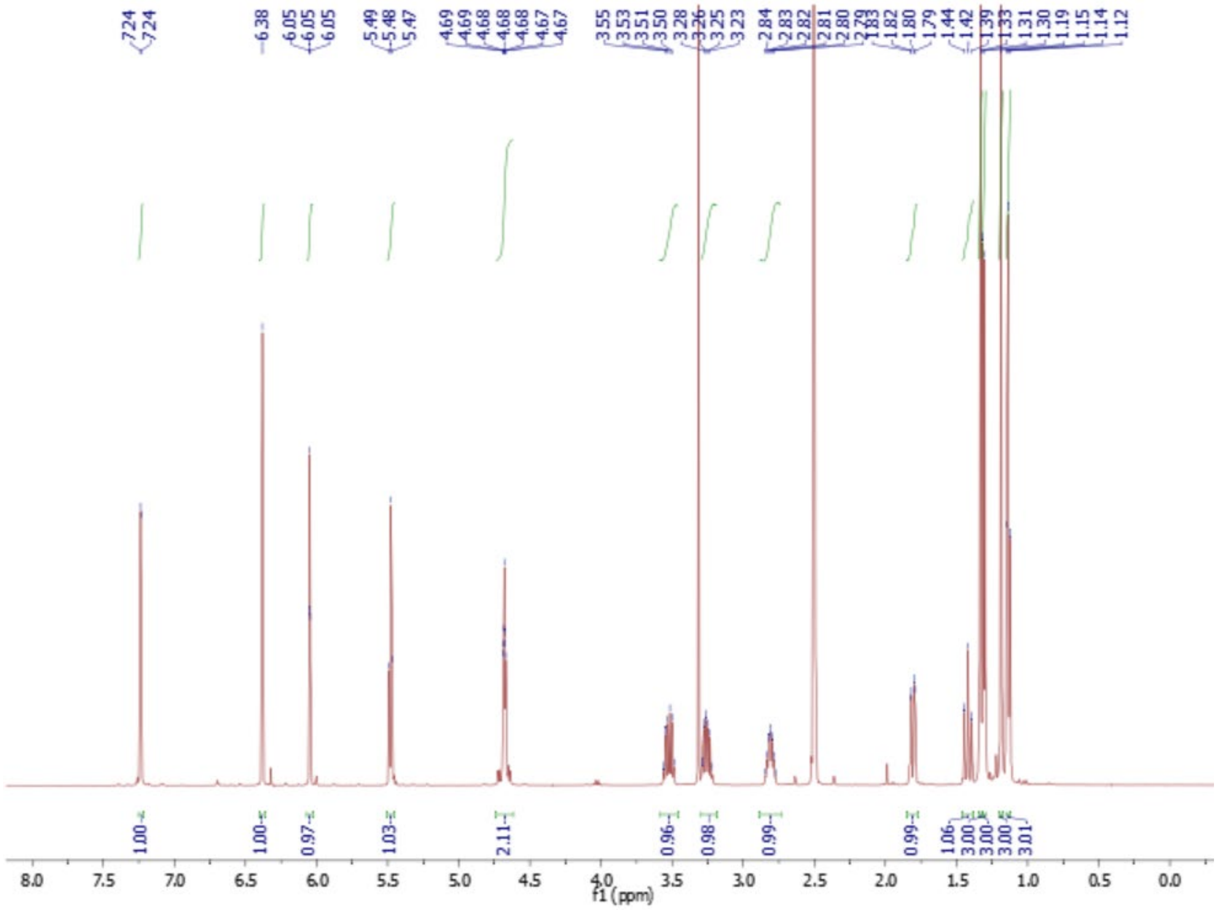

^{1}H-NMR spectrum of compound **7d** in DMSO-d$_6$.

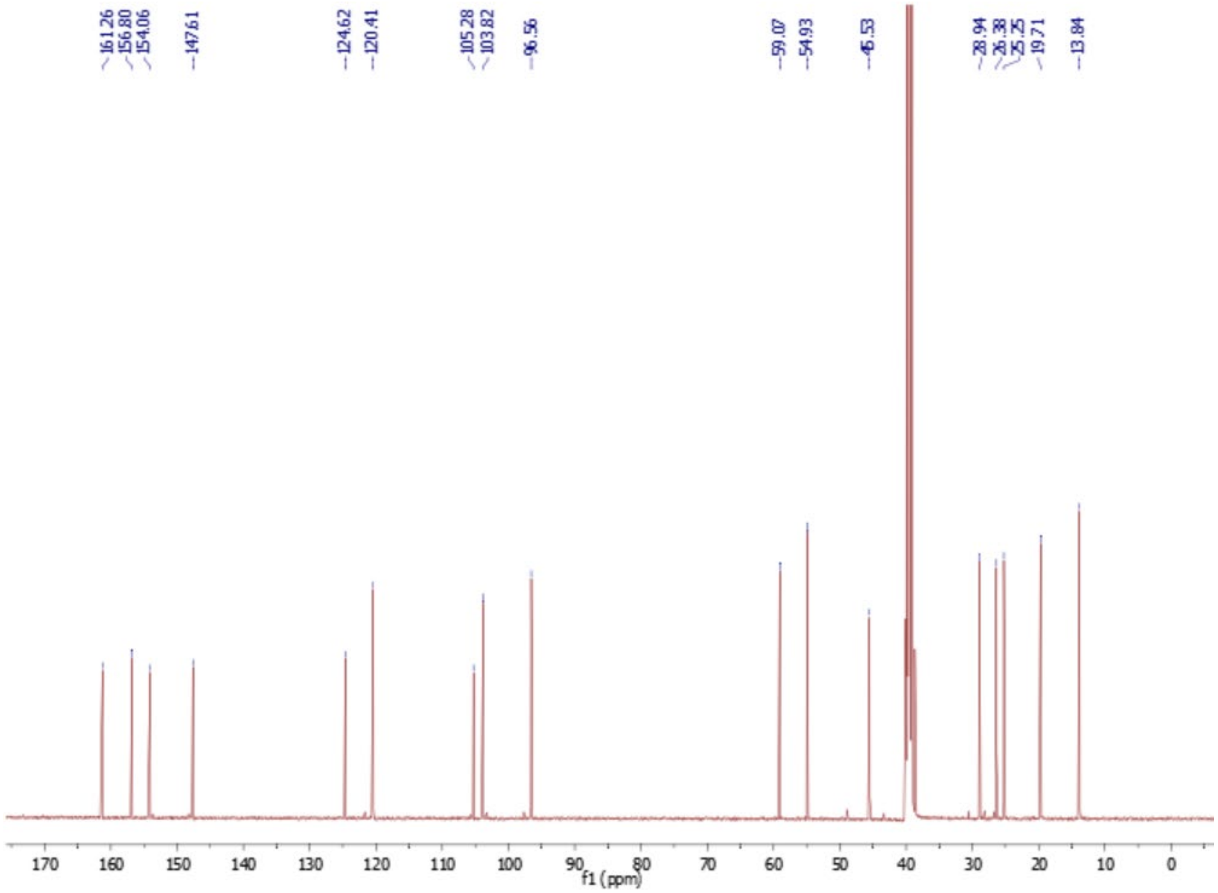

^{13}C-NMR spectrum of compound **7d** in DMSO-d_6.

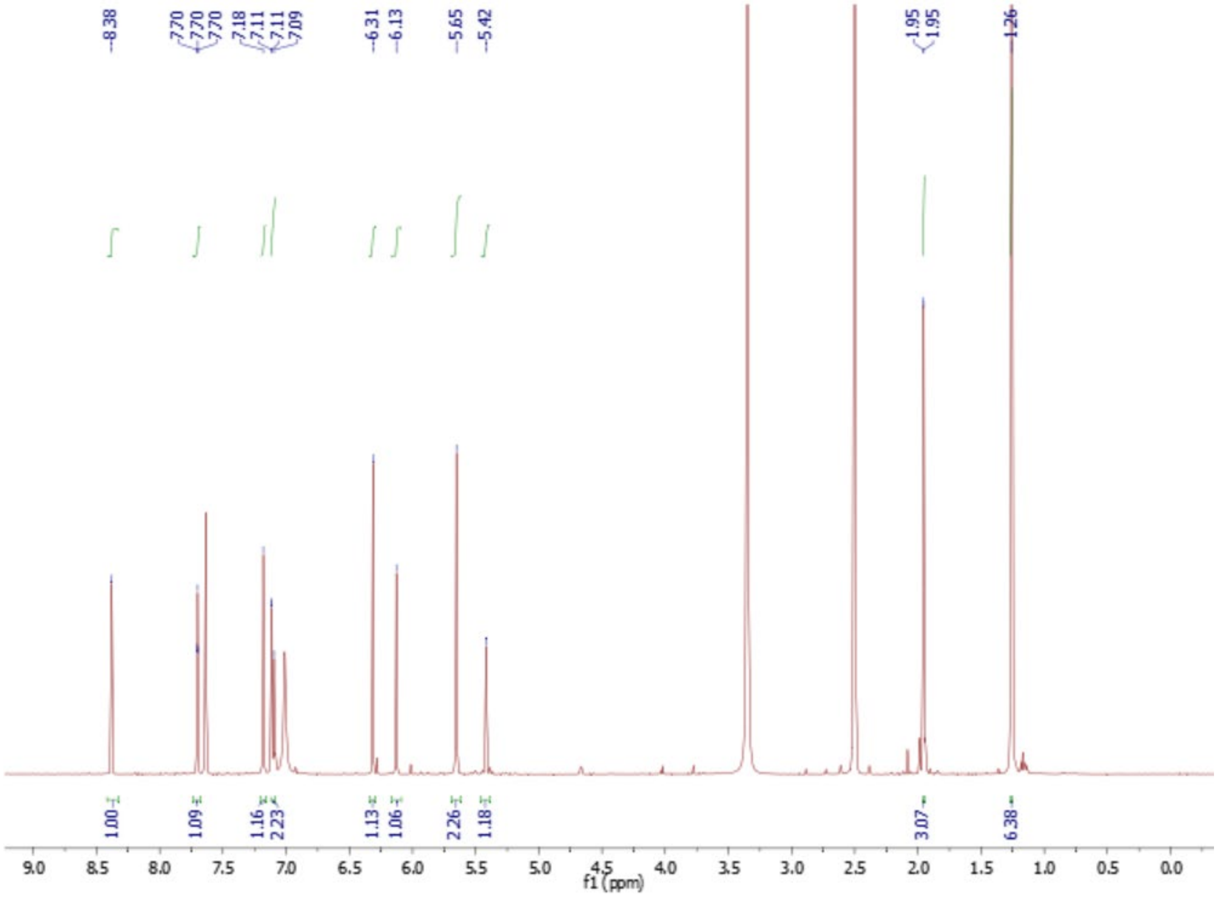

^{1}H-NMR spectrum of compound **8a** in DMSO-d_6.

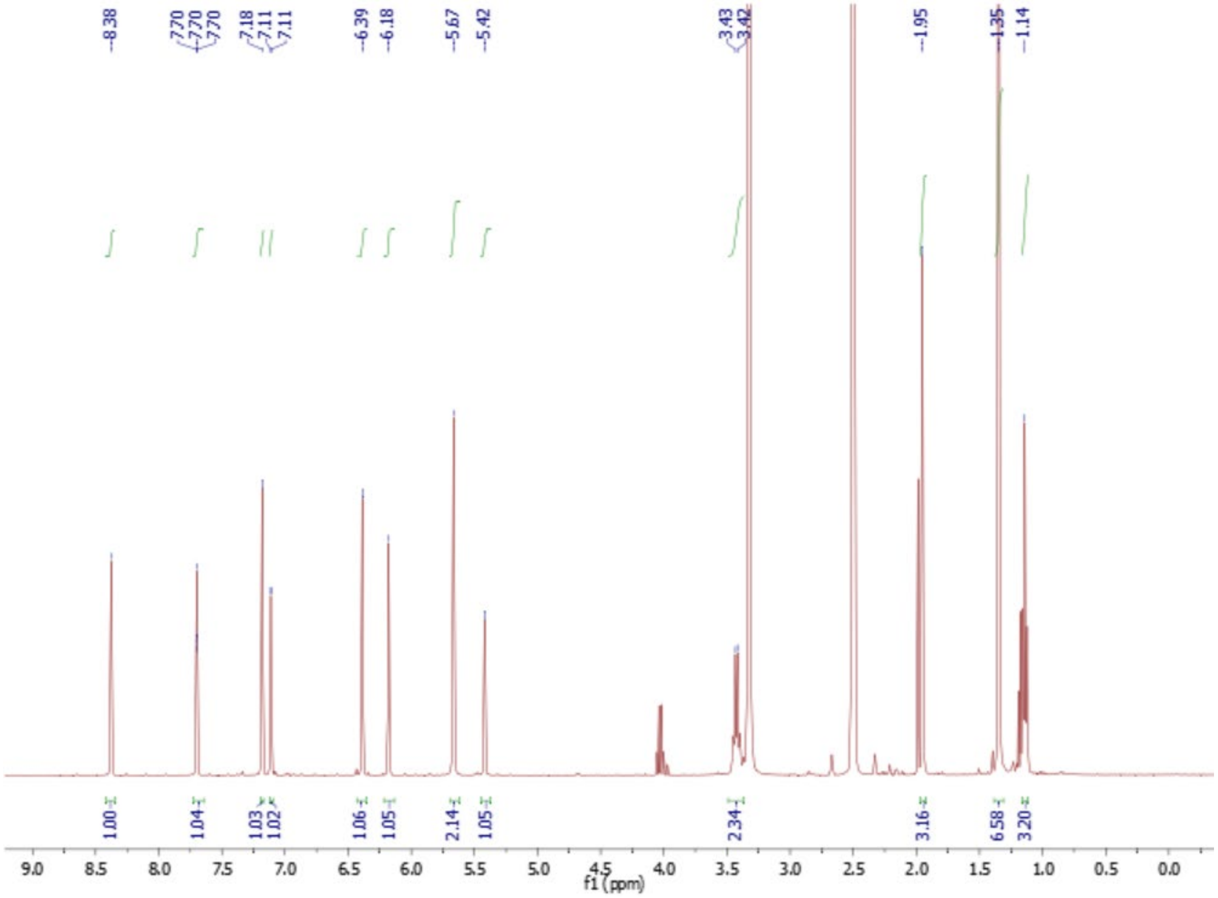

^{1}H-NMR spectrum of compound **8b** in DMSO-d_6.

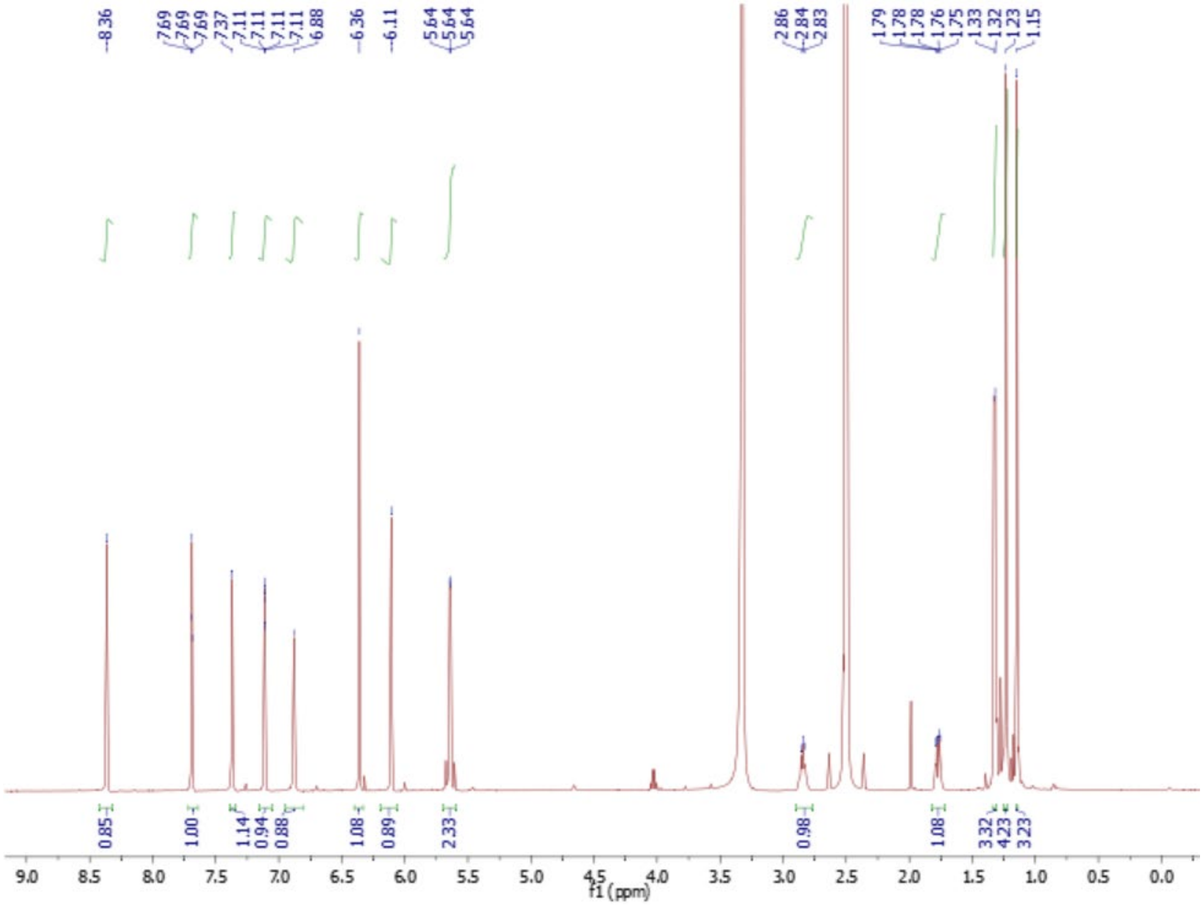

^{1}H-NMR spectrum of compound **8c** in DMSO-d_6.

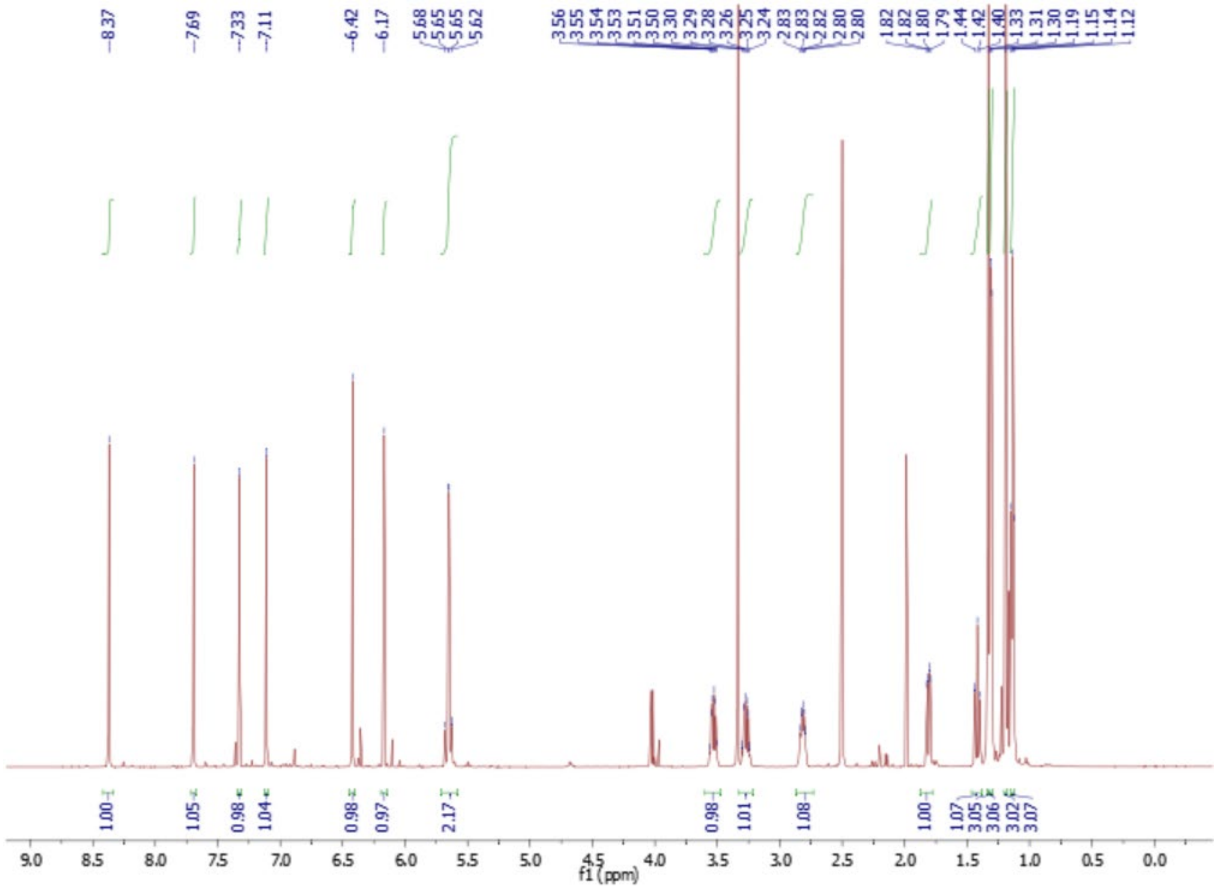

^{1}H-NMR spectrum of compound **8d** in DMSO-d_6.

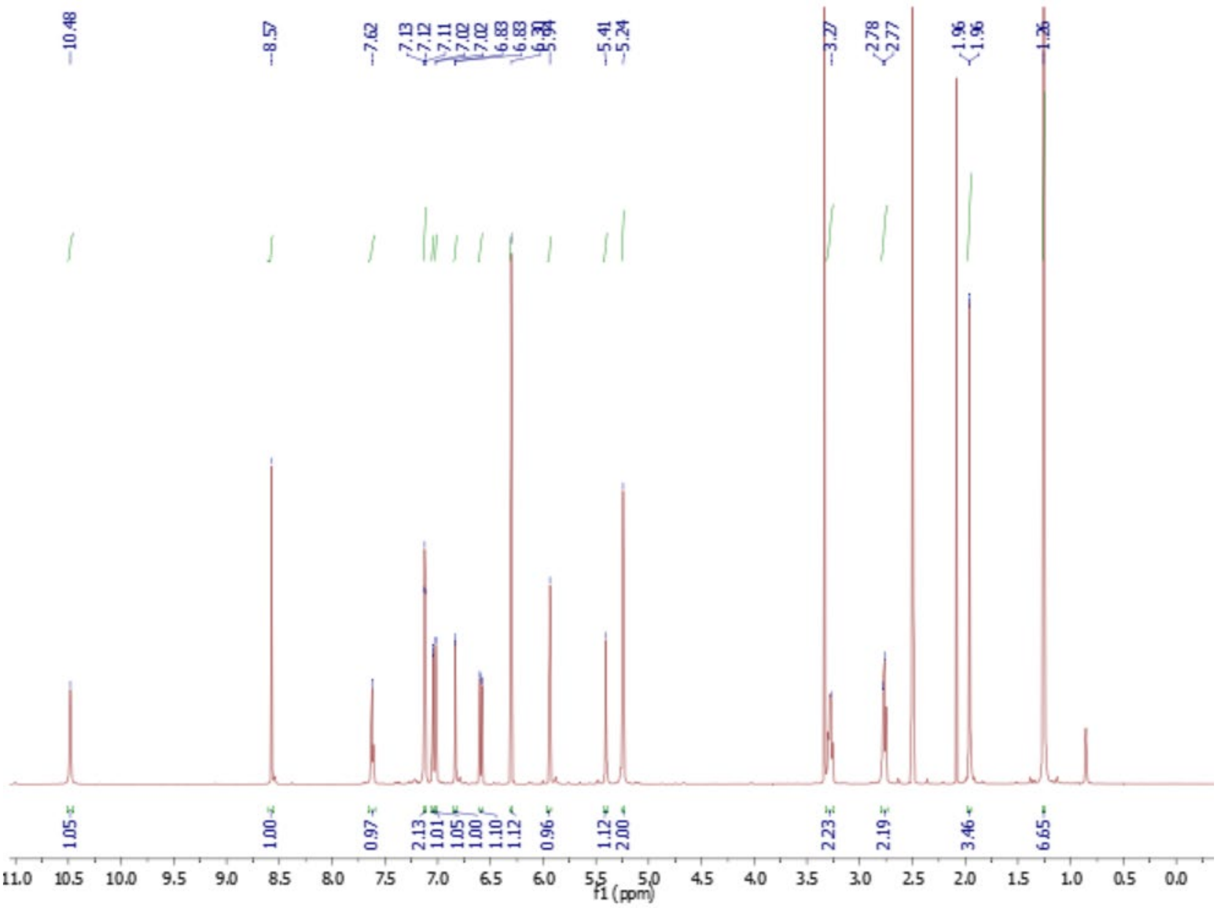

^{1}H-NMR spectrum of compound **9a** in DMSO-d_6.

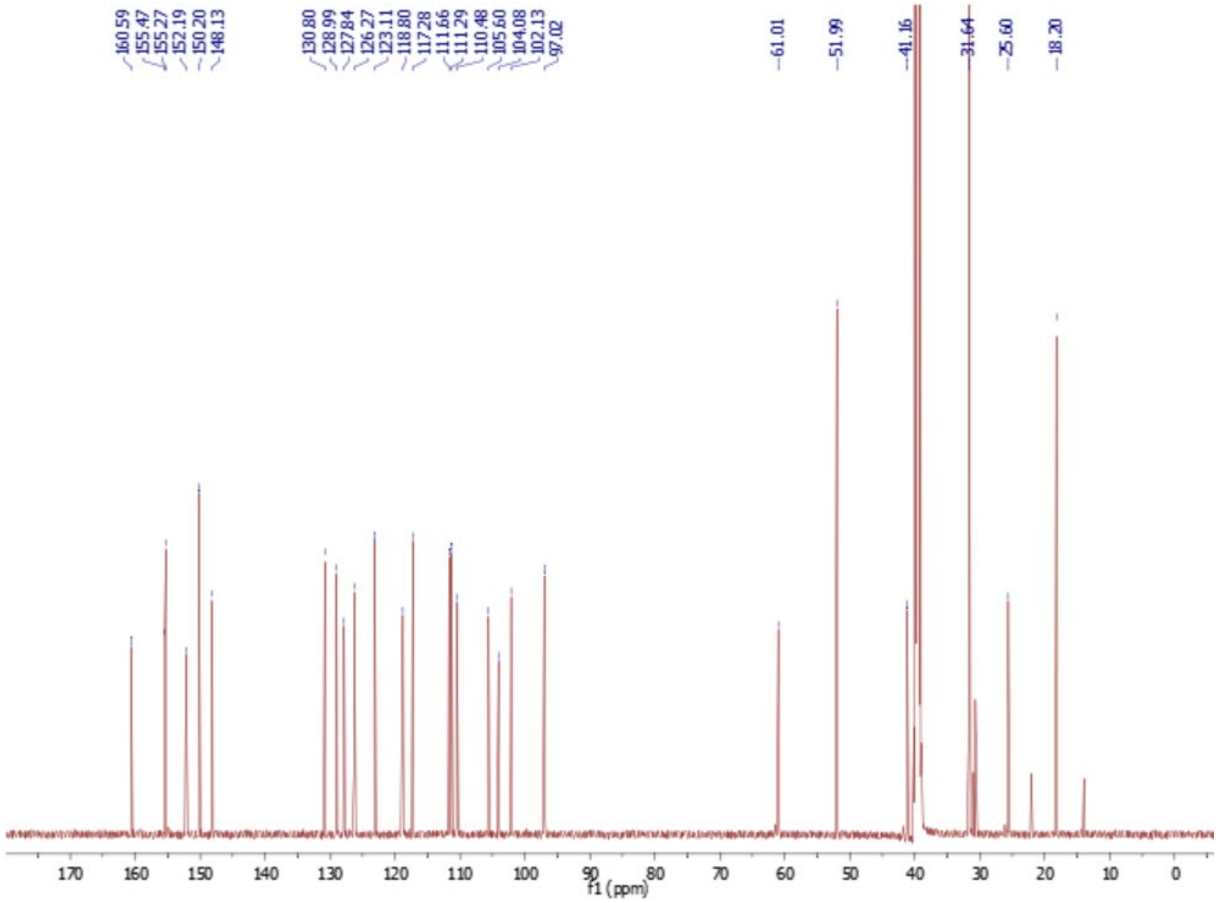

^{13}C-NMR spectrum of compound **9a** in DMSO-d$_6$.

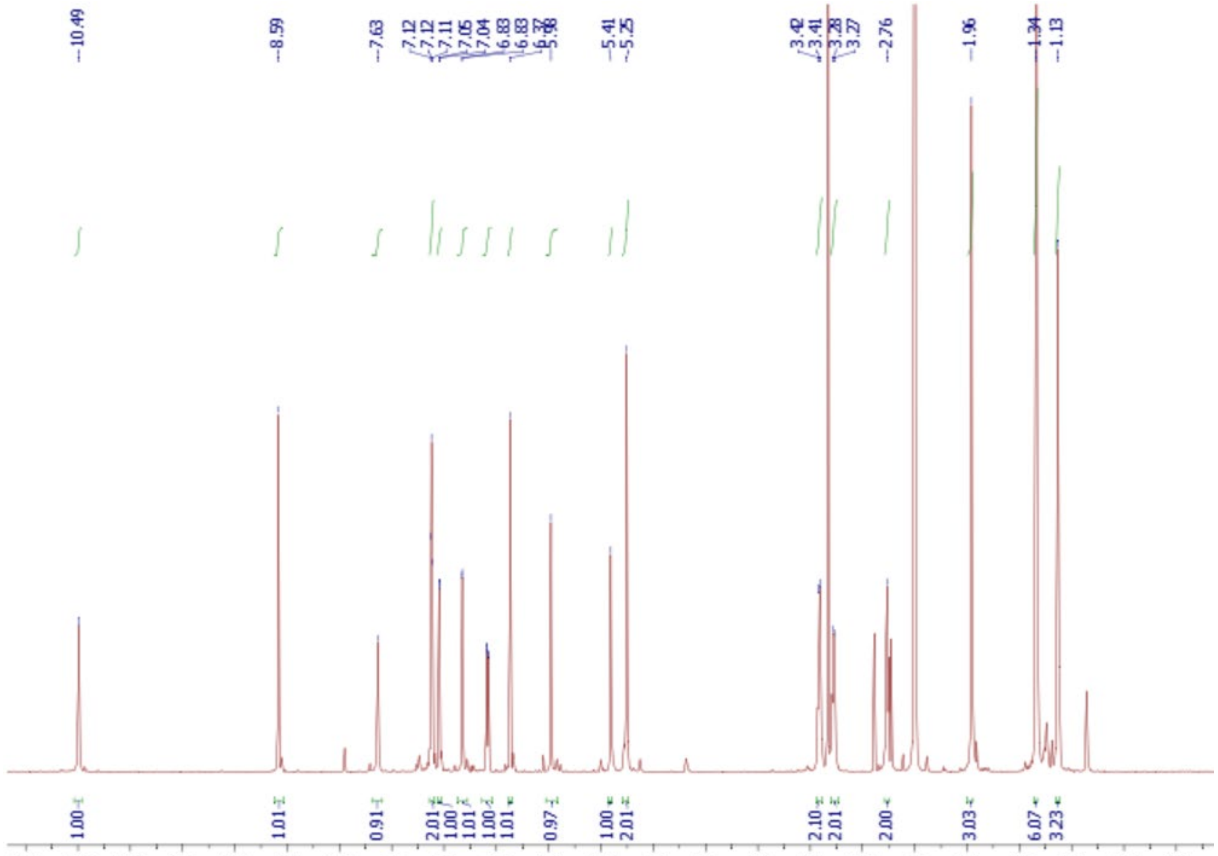

^{1}H-NMR spectrum of compound **9b** in DMSO-d$_6$.

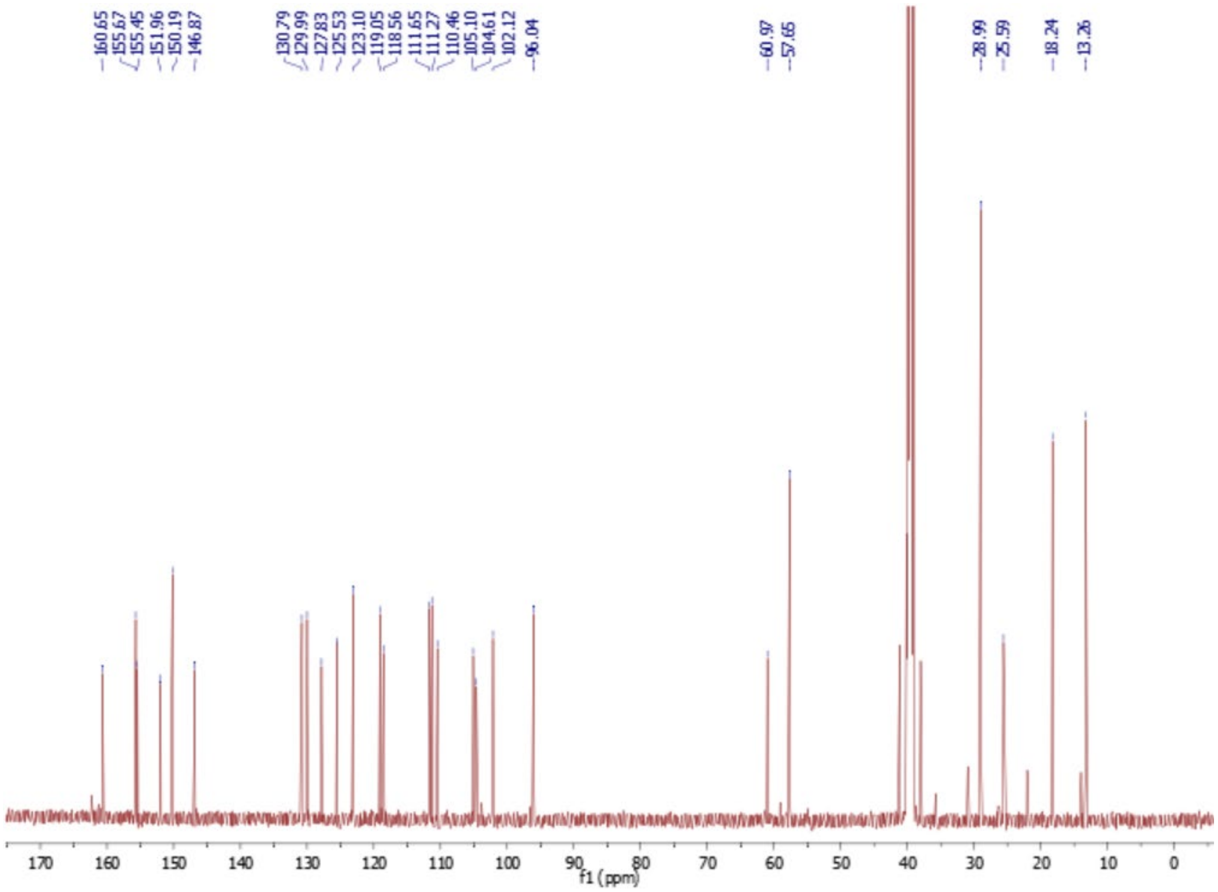

^{13}C-NMR spectrum of compound **9b** in DMSO-d$_6$.

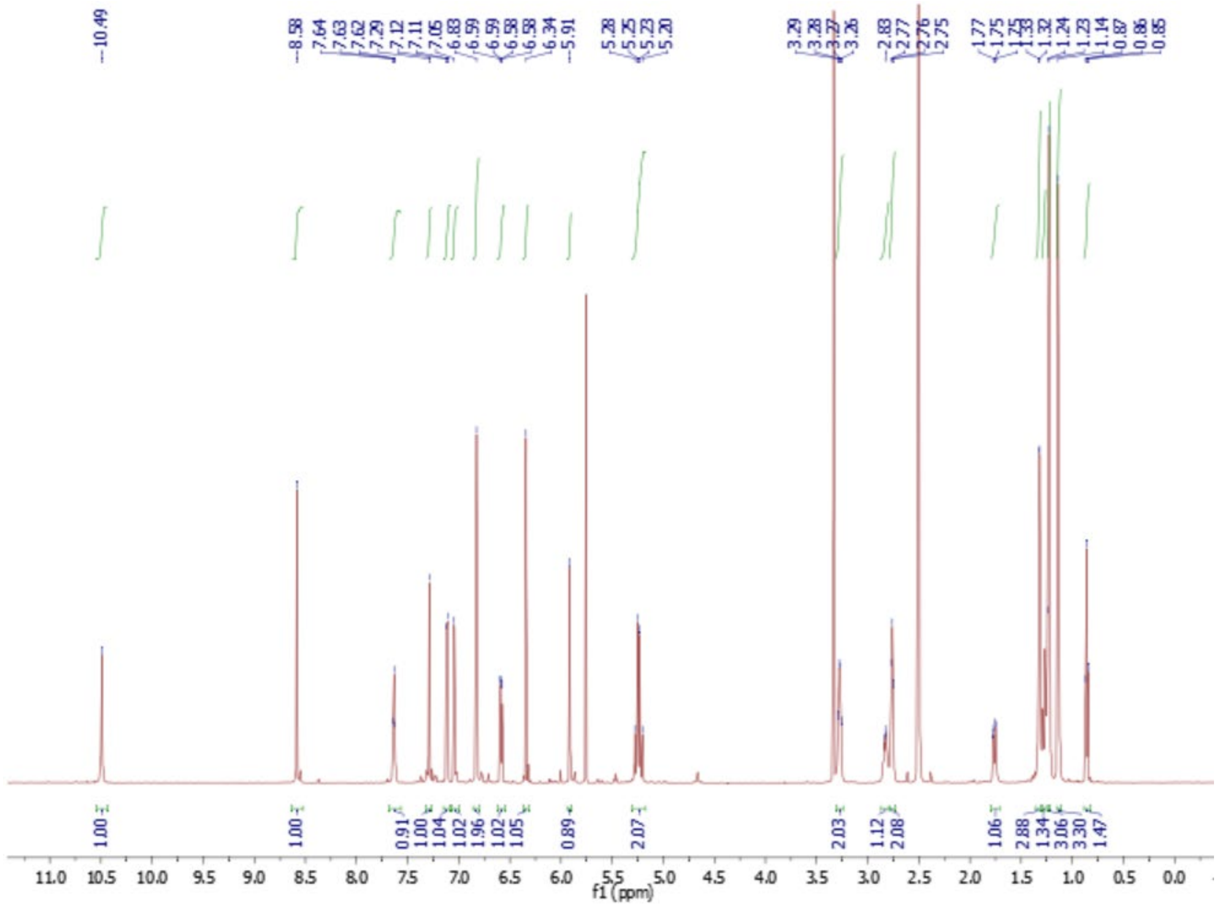

^{1}H-NMR spectrum of compound **9c** in DMSO-d$_6$.

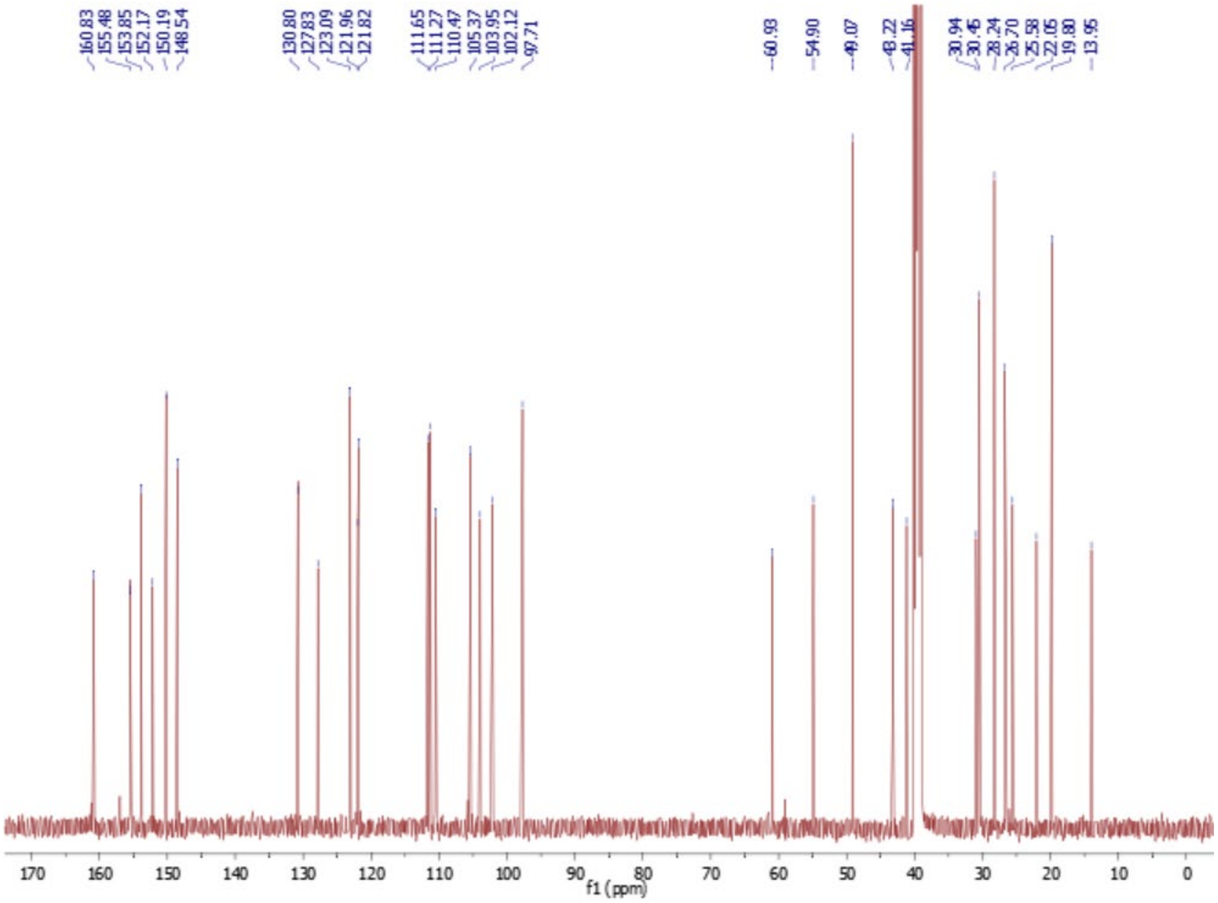

^{13}C-NMR spectrum of compound **9c** in DMSO-d_6.

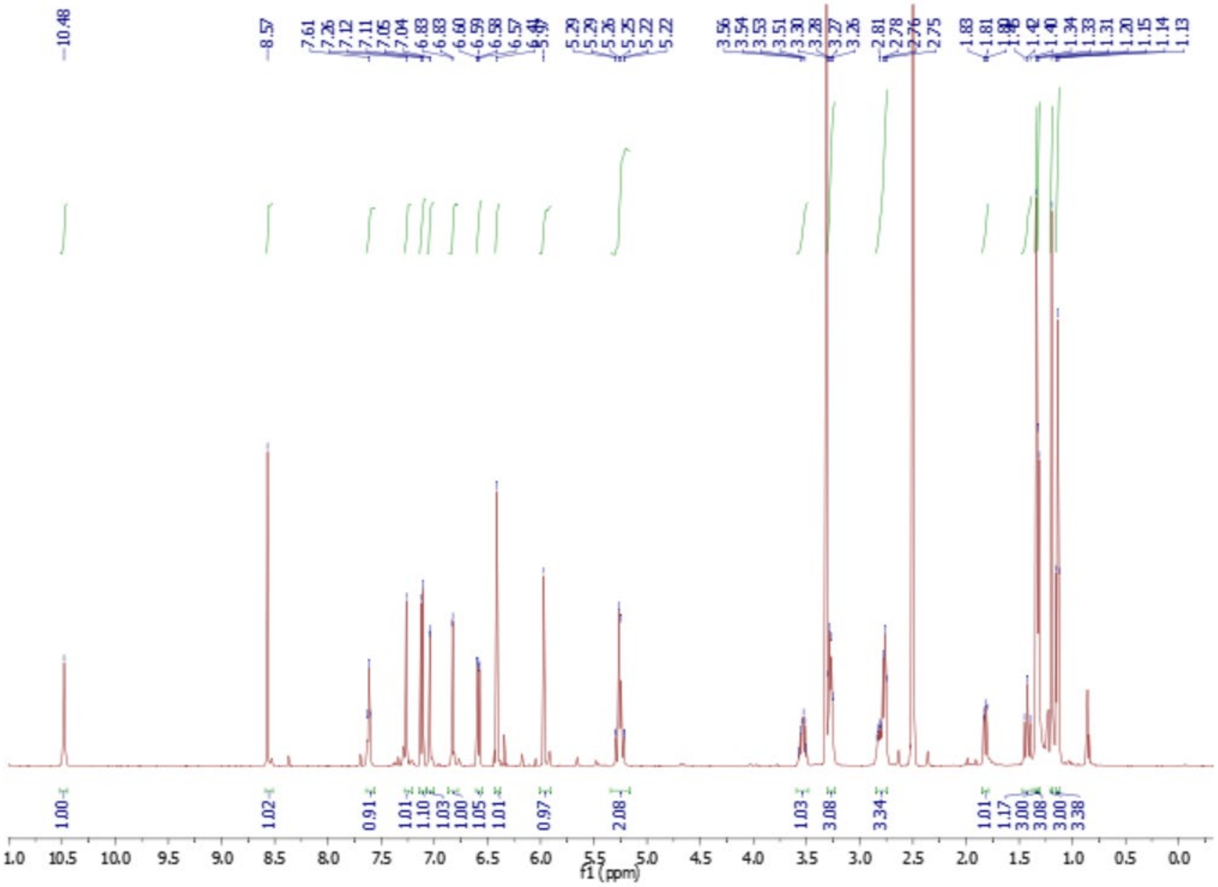

^{1}H-NMR spectrum of compound **9d** in DMSO-d_6.

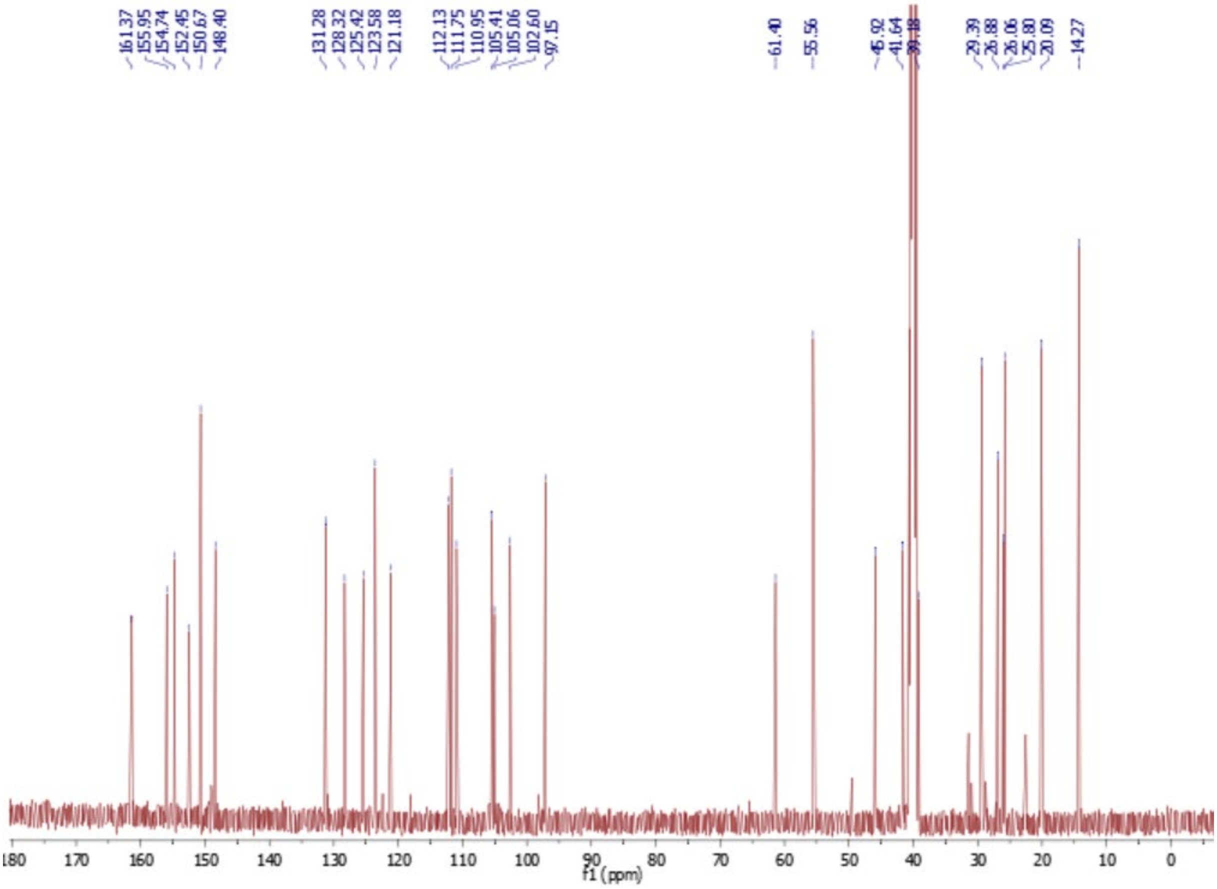

^{13}C-NMR spectrum of compound **9d** in DMSO-d$_6$.

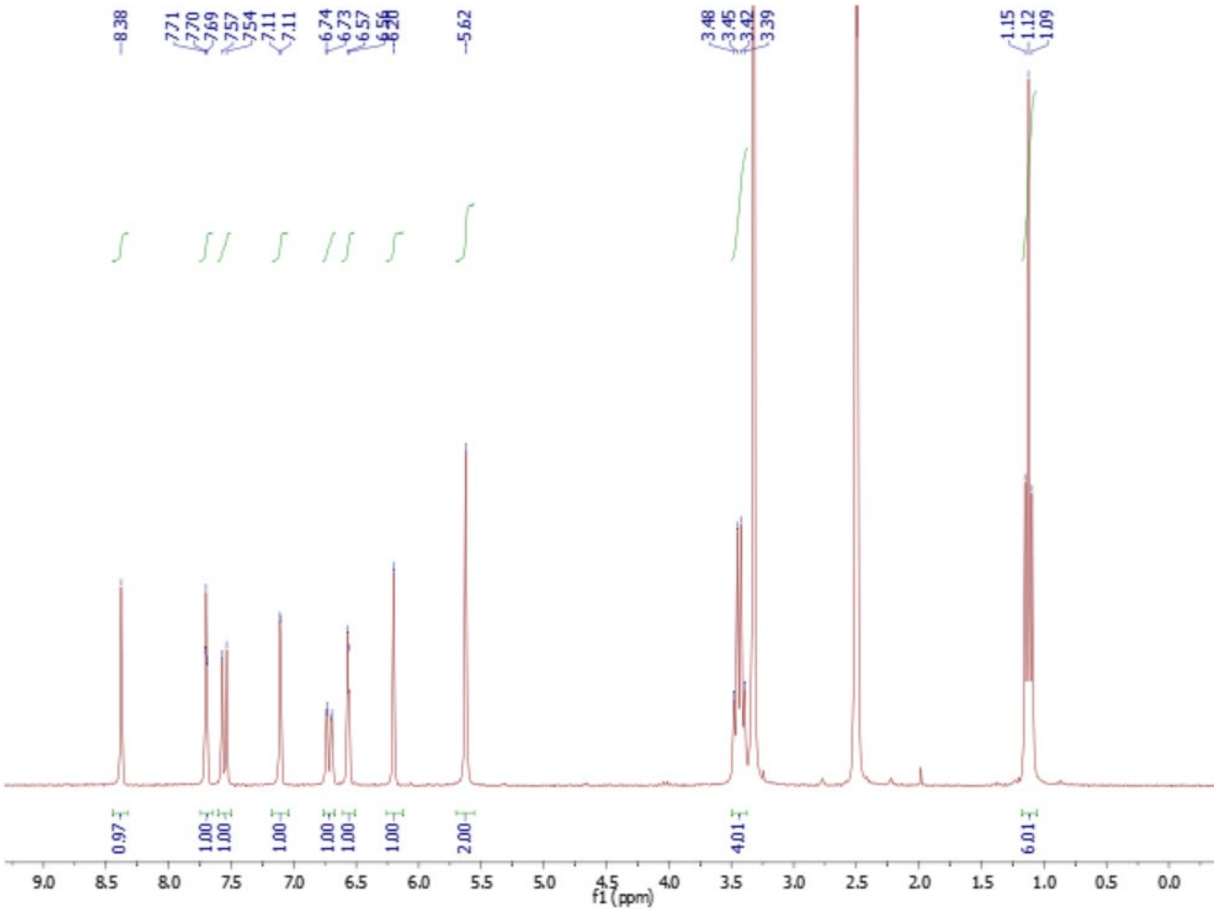

^{1}H-NMR spectrum of compound **10** in DMSO-d$_6$.

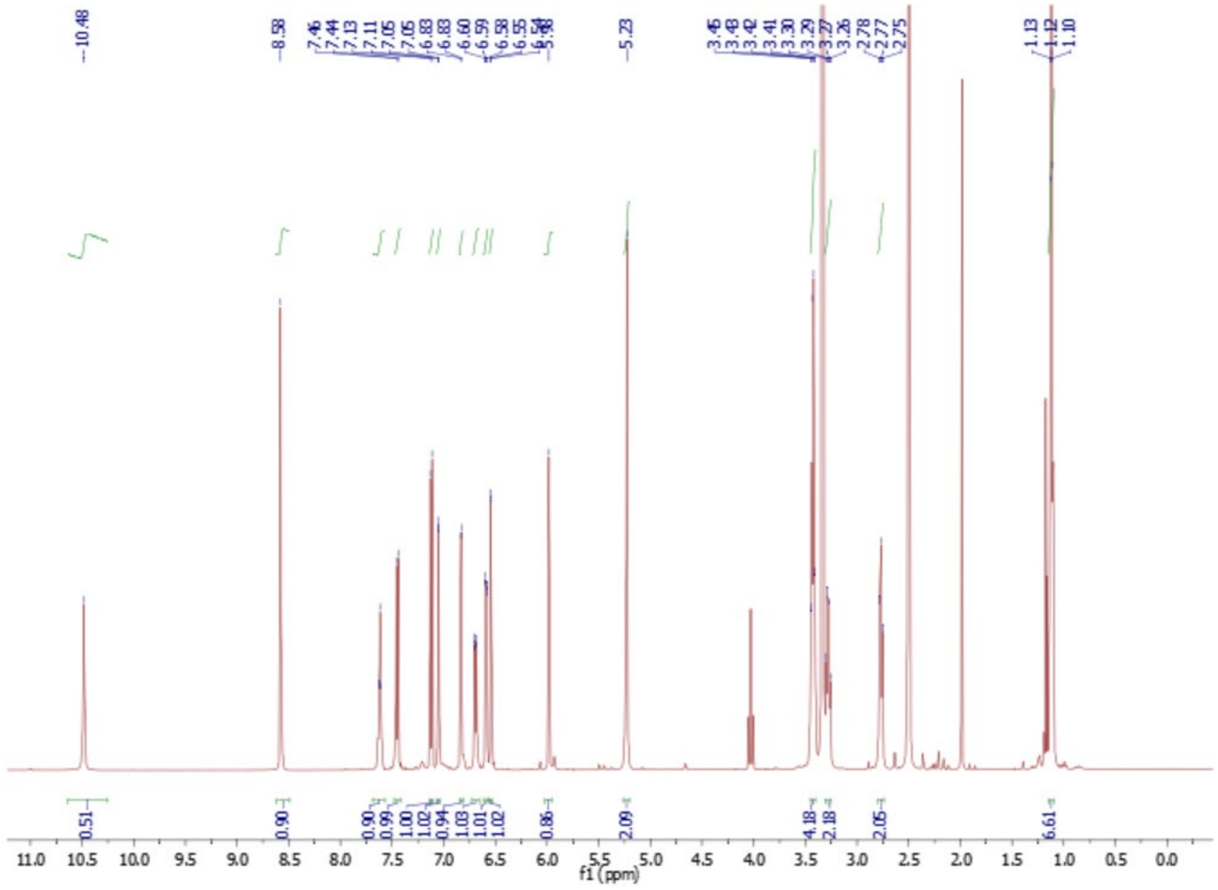

^{1}H-NMR spectrum of compound **11** in DMSO-d_6.

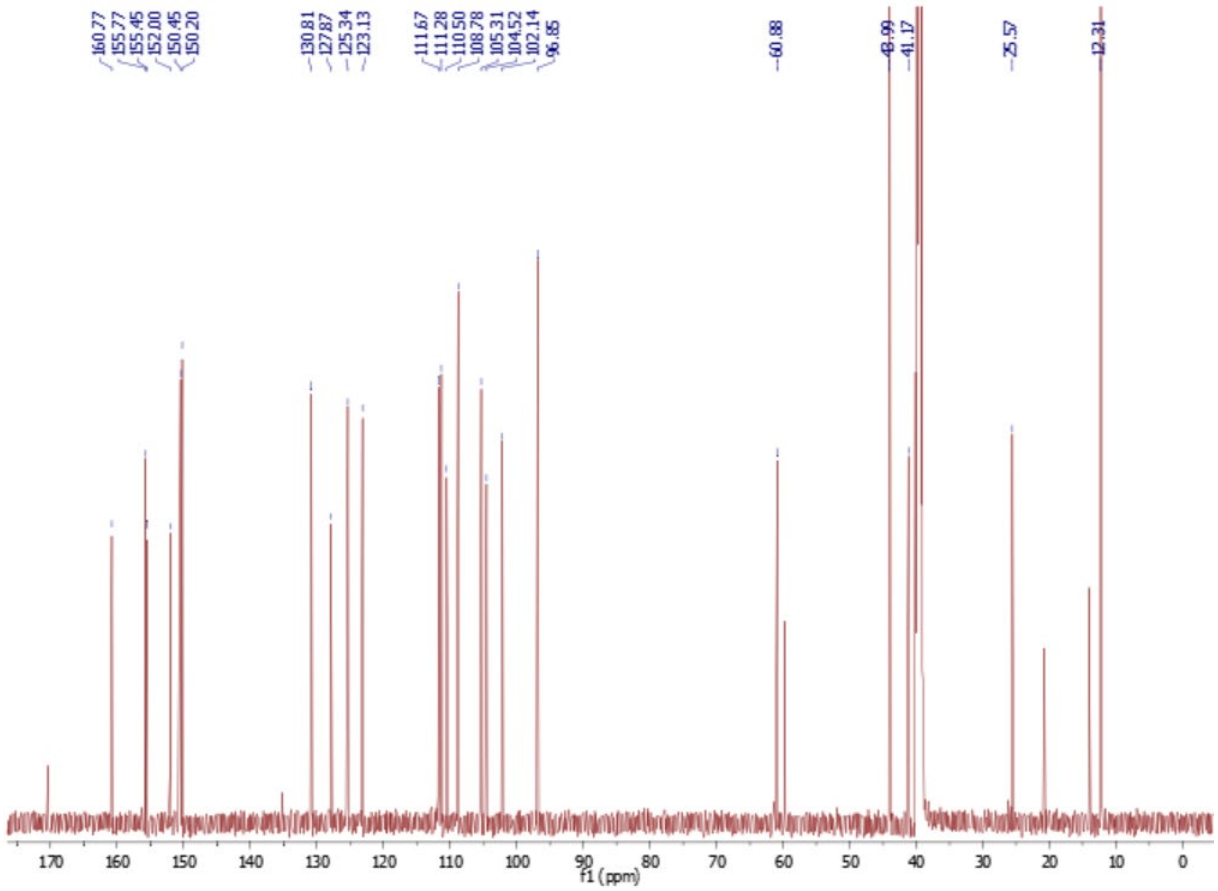

^{13}C-NMR spectrum of compound **11** in DMSO-d_6.

Mass spectra of key compounds

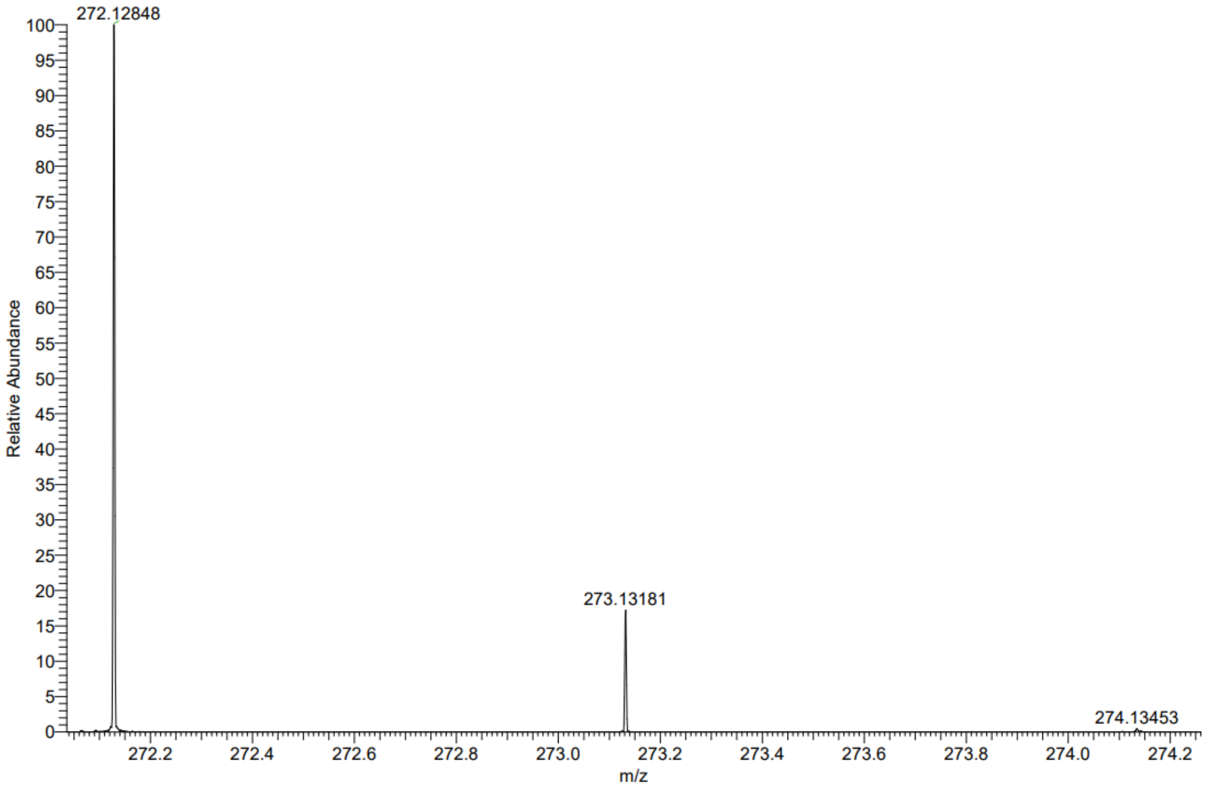

MALDI-HRMS spectrum of compound **7a**. Calculated for $C_{16}H_{17}NO_3$ $[M+H]^+$ 272.12812 (Δm = 0.00036, error 1.32 ppm).

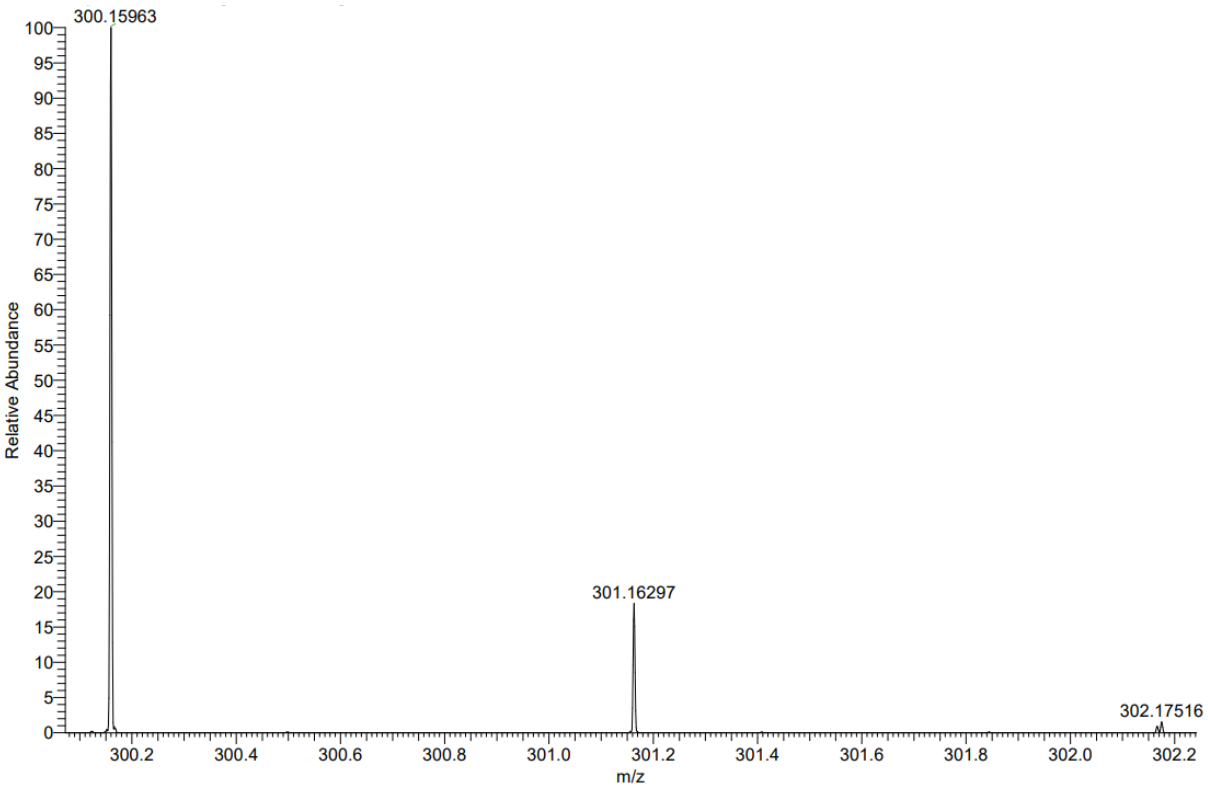

MALDI-HRMS spectrum of compound **7b**. Calculated for $C_{18}H_{21}NO_3$ $[M+H]^+$ 300.15942 (Δm = 0.00021, error 0.70 ppm).

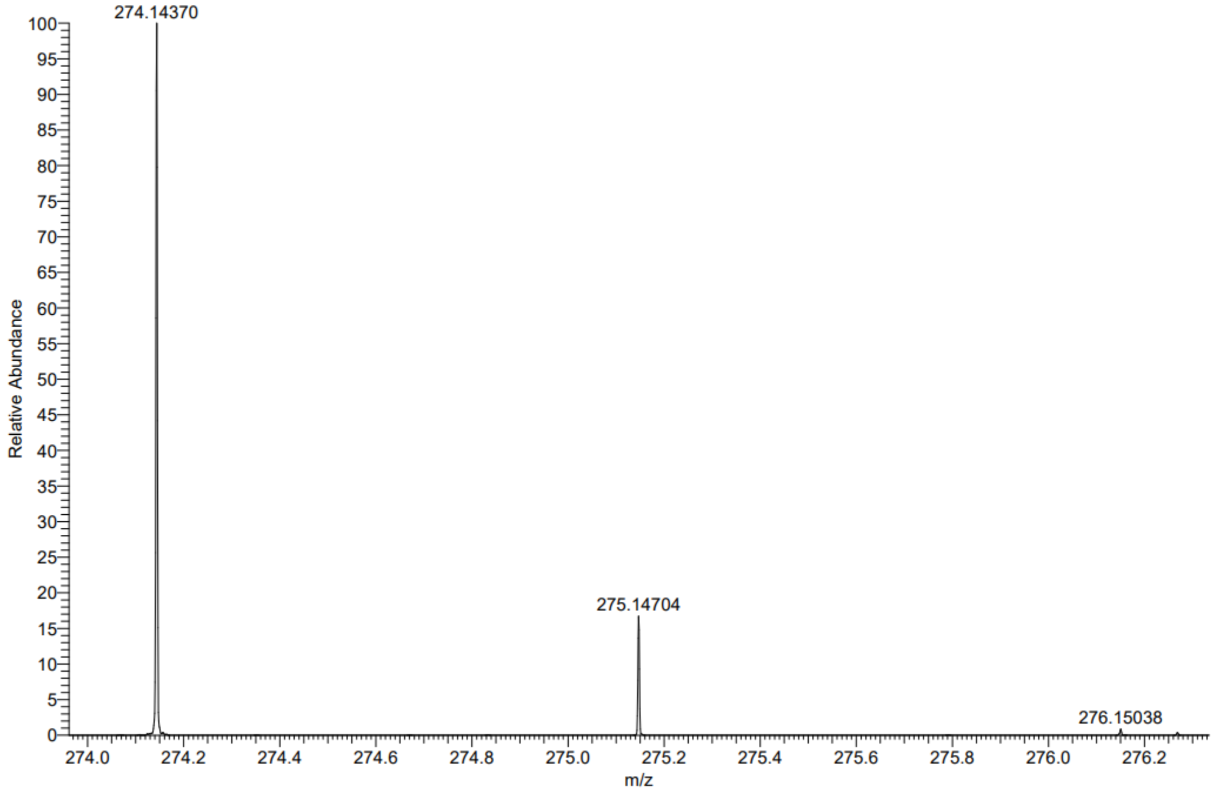

MALDI-HRMS spectrum of compound **7c**. Calculated for $C_{16}H_{19}NO_3$ $[M+H]^+$ 274.14377 (Δm = 0.00007, error 0.26 ppm).

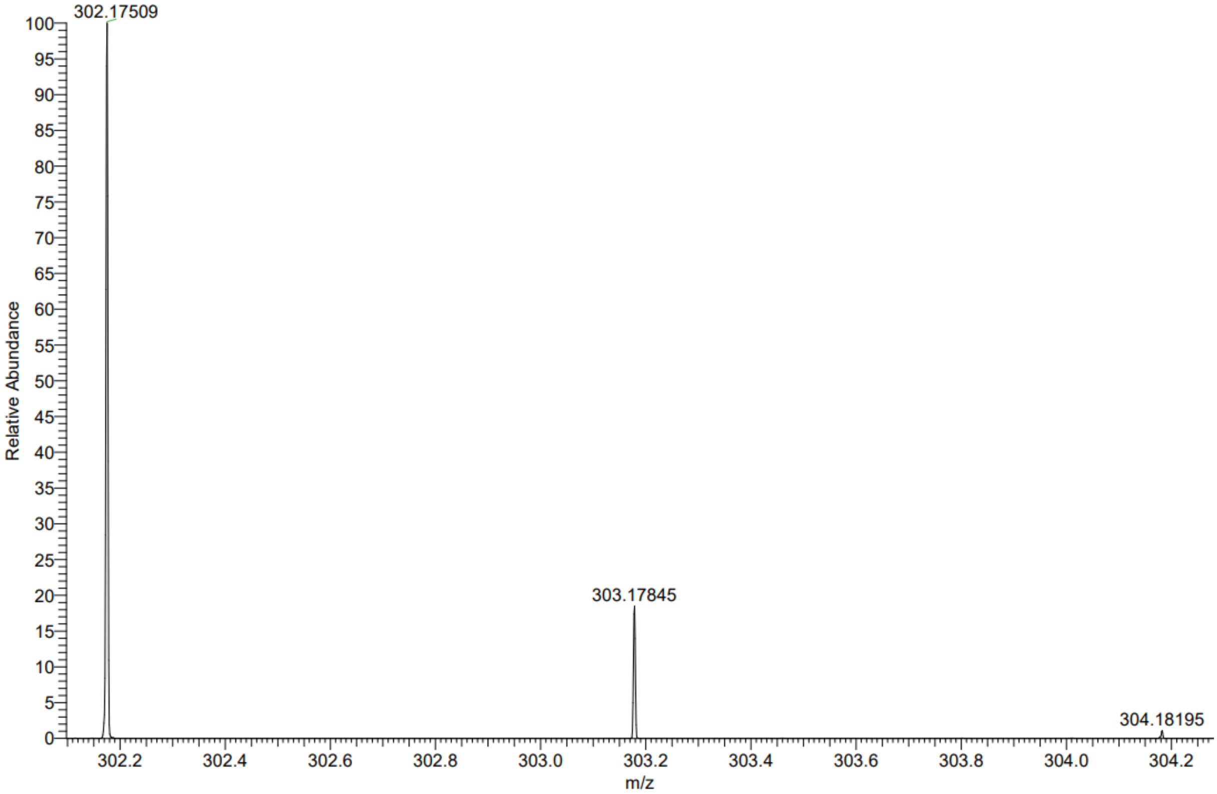

MALDI-HRMS spectrum of compound **7d**. Calculated for $C_{18}H_{23}NO_3$ $[M+H]^+$ 302.17507 (Δm = 0.00002, error 0.07 ppm).

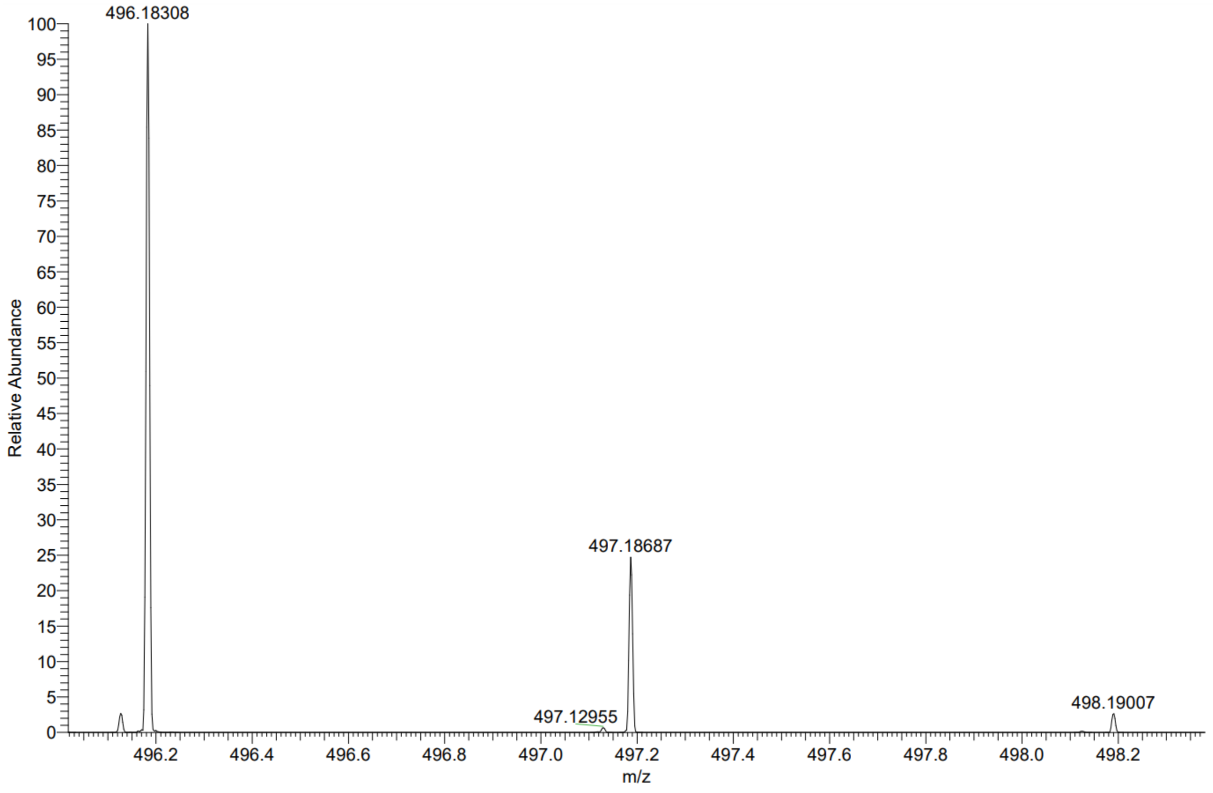

MALDI-HRMS spectrum of compound **9a**. Calculated for $C_{27}H_{27}N_3O_3$ $[M+Na]^+$ 496.18429 (Δm = 0.00121, error 2.44 ppm).

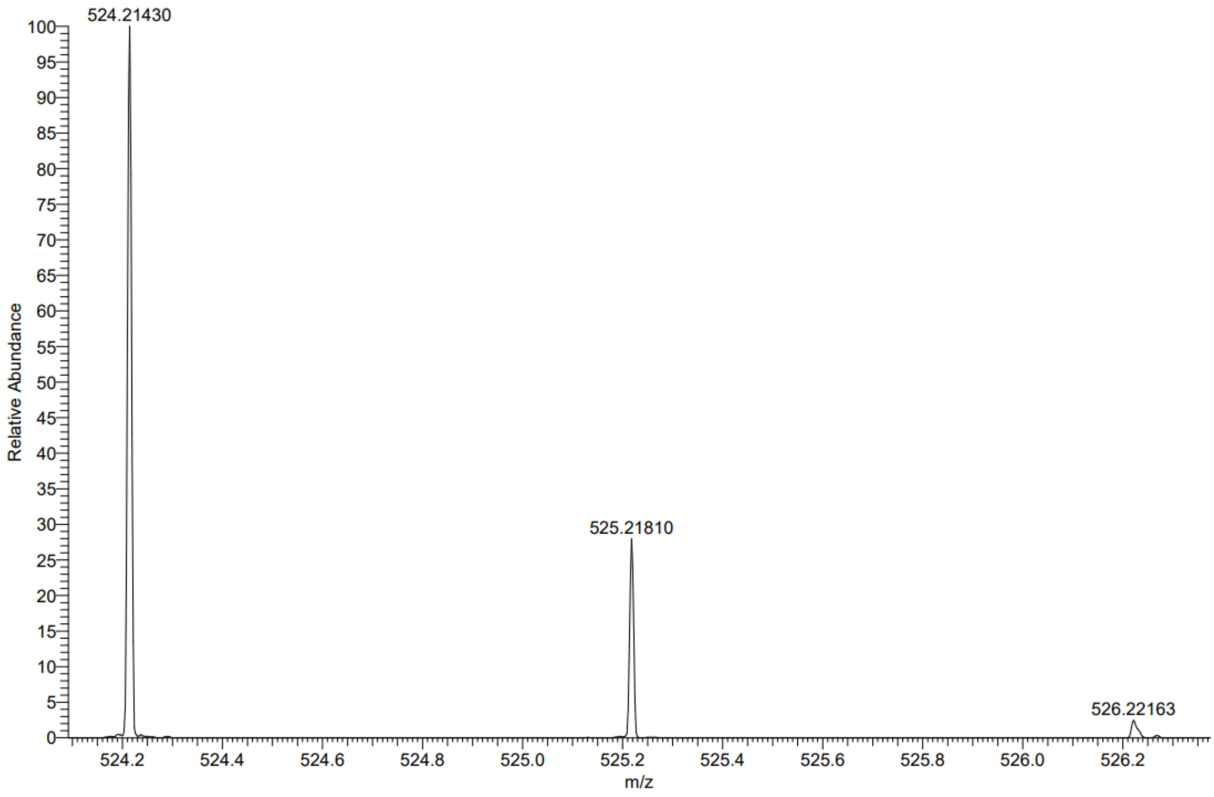

MALDI-HRMS spectrum of compound **9b**. Calculated for $C_{29}H_{31}N_3O_5$ $[M+Na]^+$ 524.21559 (Δm = 0.00129, error 2.46 ppm).

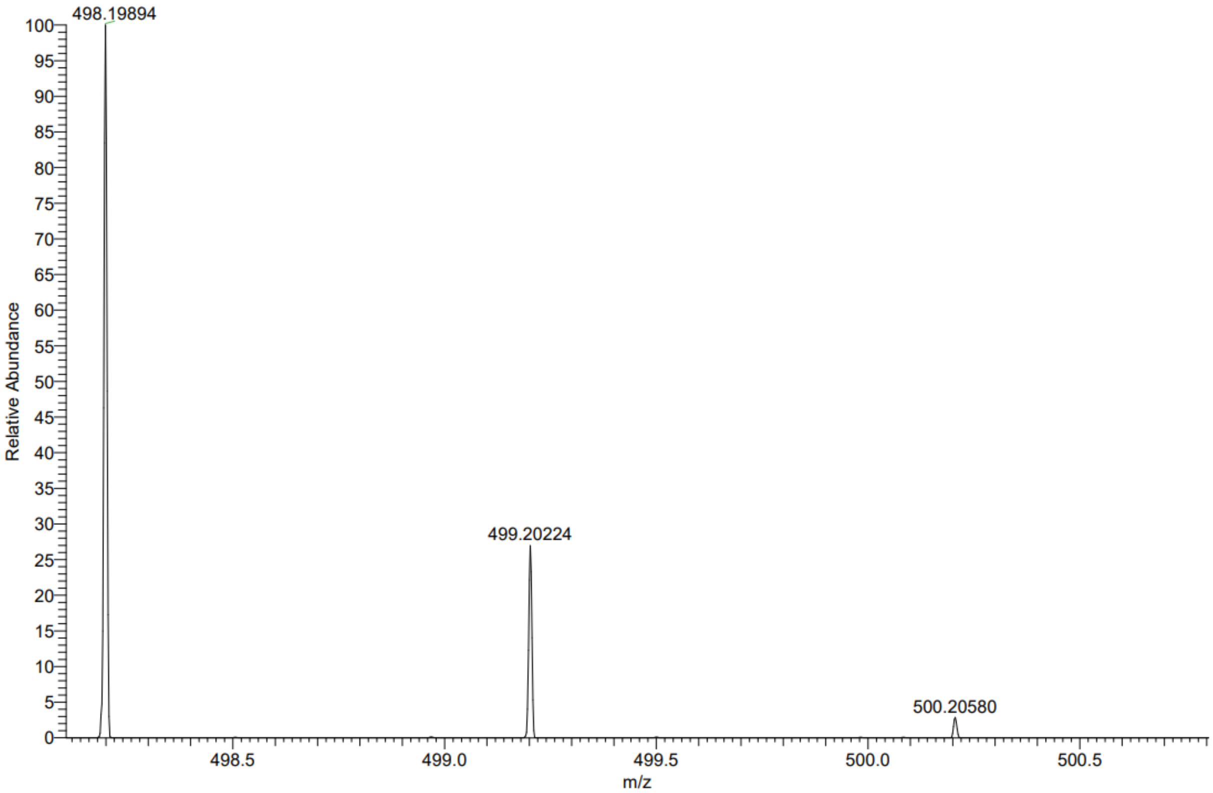

MALDI-HRMS spectrum of compound **9c**. Calculated for $C_{27}H_{29}N_3O_5$ $[M+Na]^+$ 498.19994 (Δm = 0.00100, error 2.01 ppm).

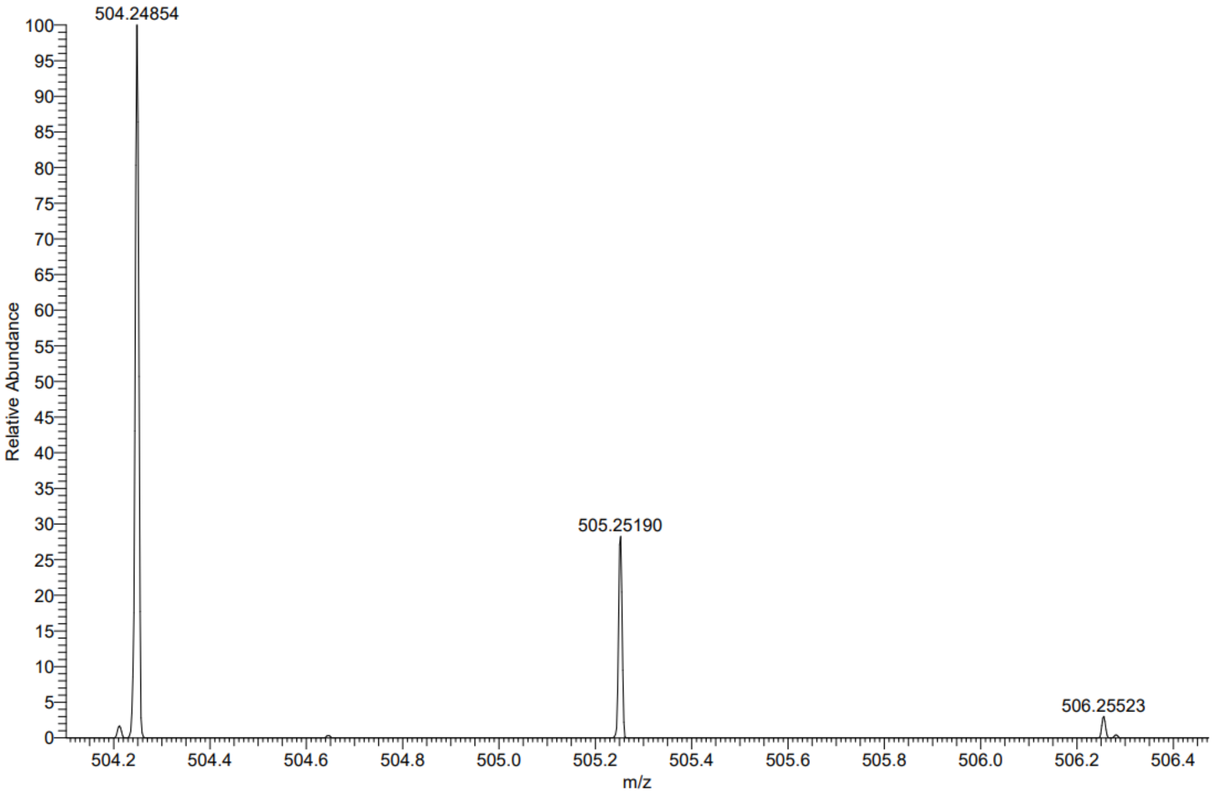

MALDI-HRMS spectrum of compound **9d**. Calculated for $C_{29}H_{33}N_3O_5$ $[M+H]^+$ 504.24930 (Δm = 0.00076, error 1.51 ppm).

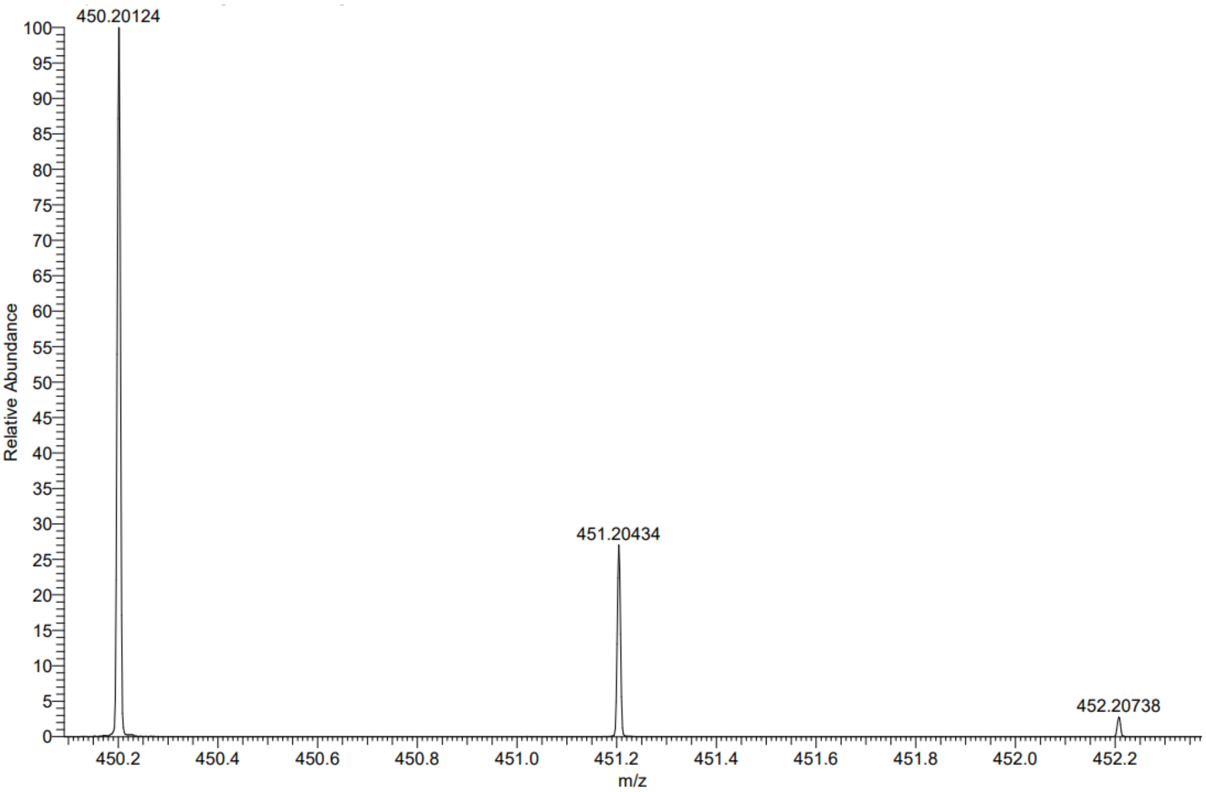

MALDI-HRMS spectrum of compound **11**. Calculated for $C_{25}H_{27}N_3O_5$ $[M+H]^+$ 450.20235 (Δm = 0.00111, error 2.50 ppm).

References

[1] T. Weinrich, M. Gränz, C. Grünewald, T. F. Prisner, M. W. Göbel, *Eur. J. Org. Chem.* **2017**, *2017*, 491–496.

[2] C. Xu, W. W. Webb, *J. Opt. Soc. Am. B* **1996**, *13*, 481.

[3] N. S. Makarov, M. Drobizhev, A. Rebane, *Opt. Express* **2008**, *16*, 4029.

[4] S. de Reguardati, J. Pahapill, A. Mikhailov, Y. Stepanenko, A. Rebane, *Opt. Express* **2016**, *24*, 9053.

[5] M. Rumi, J. W. Perry, *Adv. Opt. Photonics* **2010**, *2*, 451.

[6] J. Enderlein, R. Erdmann, *Opt. Commun.* **1997**, *134*, 371–378.

[7] C. Slavov, H. Hartmann, J. Wachtveitl, *Anal. Chem.* **2015**, *87*, 2328–2336.

[8] J. Mooney, P. Kambhampati, *J. Phys. Chem. Lett.* **2013**, *4*, 3316–3318.

[9] M. Reinfelds, V. Hermanns, T. Halbritter, J. Wachtveitl, M. Braun, T. Slanina, A. Heckel, *ChemPhotoChem* **2019**, *3*, 441–449.

[10] M. Reinfelds, J. von Cosel, K. Falahati, C. Hamerla, T. Slanina, I. Burghardt, A. Heckel, *Chem. - Eur. J.* **2018**, *24*, 13026–13035.
